# Galactokinase 1 Positively Regulates Mitochondrial Respiration by Phosphorylating TIMM13 as a Protein Kinase

**DOI:** 10.1002/advs.77996

**Published:** 2026-09-30

**Authors:** Chang Woo Ko, Dongyoon Shin, Junho Park, June Huh, Youngsoo Kim, Jong‐Wan Park

**Affiliations:** ^1^ Department of Biomedical Sciences College of Medicine Seoul National University Seoul Republic of Korea; ^2^ Department of Medical Science School of Medicine CHA University Seongnam Republic of Korea; ^3^ Department of Medical Science Research Institute for Basic Medical Science School of Medicine CHA University Seongnam Republic of Korea; ^4^ Department of Chemical and Biological Engineering Korea University Seoul Republic of Korea; ^5^ Department of Pharmacology College of Medicine Seoul National University Seoul Republic of Korea; ^6^ Ischemic/Hypoxic Disease Institute Seoul National Medical Research Center Seoul National University Seoul Republic of Korea

**Keywords:** GALK1, mitochondrial respiration, protein kinase, TIMM13

## Abstract

Galactokinase 1 (GALK1) is a metabolic kinase that phosphorylates galactose. Mutations in GALK1 cause type II galactosemia, which manifests as cataracts and extra‐ocular symptoms. The mechanisms underlying these symptoms are not fully understood. In this study, we explored a novel function of GALK1 beyond galactose phosphorylation. GALK1 is ubiquitously expressed across human tissues, regardless of developmental stage. GALK1 phosphorylates TIMM13 at the Y73 residue. The phosphorylated Y73 stabilizes TIMM13 by electrostatically interacting with two cationic residues, thereby enhancing its affinity for Zn^2^^+^. This structural change ensures the mitochondrial translocation of TIMM13 in the correct conformation. Disruption of Y73 phosphorylation impairs mitochondrial bioenergetics and dissipates the mitochondrial membrane potential. Furthermore, galactose competitively inhibits GALK1‐mediated phosphorylation, suggesting that mitochondrial activity is modulated by carbohydrate availability. Galactosemia‐associated GALK1 mutations showed compromised activity in TIMM13 phosphorylation, subsequently repressing mitochondrial respiration. Collectively, the GALK1‐TIMM13 axis appears to provide a potential link between galactose metabolism and mitochondrial respiration.

## Graphical abstract

GALK1 acts as a protein kinase beyond the phosphorylation of galactose. GALK1 phosphorylates TIMM13 at Y73 in the cytoplasm. This phosphorylation prevents premature oxidative folding of TIMM13 and ensures its entrance into the intermembrane space of mitochondrion, positively regulating mitochondrial respiration. TIMM13 may be dephosphorylated by protein tyrosine phosphatase (PTP) or dual specificity protein phosphatase (DSP) before its maturation within mitochondria. The GALK1‐TIMM13 axis provides a potential link between galactose metabolism and mitochondrial respiration.

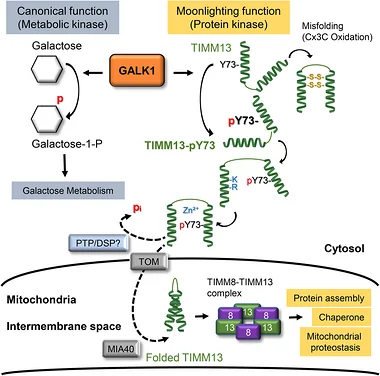

## 1 Introduction

The enzyme galactokinase 1 (GALK1) phosphorylates galactose to produce galactose‐1‐phosphate, constituting the first step in the Leloir pathway [1]. This reaction permits galactose, which is largely derived from dietary milk, to be utilized as an energy source. Because of such a central role of GALK1, a loss‐of‐function mutation of the *GALK1* gene is expected to cause a metabolic disorder in infants or young children. Indeed, some variations in the *GALK1* gene have been identified to cause Type II galactosemia, which is a rare autosomal recessive disorder marked by the accumulation of galactose and its metabolites (galactitol and galactonate). In early infancy, this disease often manifests as bilateral cataracts and occasionally as other symptoms. The clinical progress of this disease can be prevented by early intervention with a galactose‐restricted diet [1].

Clinically, GALK1 deficiency does not result in serious health problems other than cataract. Nevertheless, a subset of patients—particularly those with delayed diagnosis or poor dietary adherence—present with extra‐ocular symptoms such as neurodevelopmental delay, microcephaly, hearing impairment, and metabolic disturbances [2, 3]. Even in the patients maintaining a galactose‐restricted diet, some complications, such as delayed development, dyspraxia, intellectual disability, liver injury, kidney dysfunction, and hypogonadism, have been reported. These reports imply that the disease phenotypes in GALK1 deficiency may occur irrespective of deregulation of galactose metabolism. Thus, we assumed that the GALK1 dysfunction per se could affect cellular processes contributing to the disease phenotypes.

Recently, some kinases, which are originally known to phosphorylate metabolites, have been identified to phosphorylate specific proteins. Such moonlighting functions of metabolic kinases represent a paradigm shift in our understanding of cell physiology—a close relationship between the metabolic state and cell signaling pathway [4]. The examples are the enzymes involved in glucose metabolism, such as PGK1, PKM2, PFKFB4, and PCK1, and their protein substrates are distributed across the nucleus, the cytosol and the mitochondrion [5]. In particular, hexokinase 2 (HK2) and ketohexokinase A (KHK‐A) in charge of the first step of monosaccharide metabolism have been reported to act as protein kinases that regulate specific signaling pathways and gene expression. For instance, HK2 induces the degradation of IκBα by directly phosphorylating it at Thr291, which facilitates the NF‐κB‐dependent PD‐L1 expression and tumor immune evasion [6]. Furthermore, HK2 phosphorylates nSMase1 in hypoxic astrocytes, thereby promoting the biogenesis of intraluminal vesicles and the release of injurious exosomes [7]. KHK‐A represents another prominent example of a metabolic enzyme having protein kinase activity. The *KHK* gene expresses two RNA splicing variants KHK‐A and KHK‐C. KHK‐C is the main metabolic enzyme to convert fructose to fructose‐1‐phosphate. In contrast, KHK‐A, having a lower affinity for fructose, functions as a protein kinase. In the cytoplasm, KHK‐A phosphorylates PRPS1 at Thr225 to stimulate *de novo* nucleic acid synthesis, and also phosphorylates p62 at Ser28 to finally activate the Nrf2 antioxidant response. In the nucleus, KHK‐A phosphorylates YWHAH at Ser25, promoting the SLUG‐driven repression of the *CDH1* gene and inducing cancer metastasis [8, 9, 10]. These findings establish that monosaccharide kinases may share a latent structural capacity for protein kinase activity. Human beings can utilize three types of monosaccharides—glucose, fructose, and galactose. Given that HK and KHK are the first step enzymes to metabolize glucose and fructose, we were interested in the GALK enzyme responsible for the first step metabolism of galactose. In the present study, we checked the possibility that GALK1 acts as a protein kinase beyond its activity as a monosaccharide kinase.

TIMM13 (Translocase of Inner Mitochondrial Membrane 13) forms a chaperone complex with its partner TIMM8A within the intermembranous space of mitochondrion [11, 12]. The complex facilitates the import of hydrophobic precursor proteins into the mitochondrial inner membrane by ferrying them through the intermembranous space to the TIM17, TIM22 and TIM23 [11, 13]. Proper functioning of this import machinery is crucial for mitochondrial protein homeostasis, as disruption of the TIMM complexes can lead to mitochondrial dysfunction [14, 15]. To date, TIMM13 has been studied solely in respect of its actions on mitochondrial metabolism. However, what is the upstream regulator of TIMM13 has not been intensively investigated.

Here, we discovered that GALK1 functions as a protein kinase for TIMM13. The GALK1‐TIMM13 axis is likely to be essential for the maintenance of mitochondrial respiration. The GALK1 mutations clinically manifesting Type II galactosemia failed to phosphorylate TIMM13, leading to mitochondrial dysfunction. The GALK1‐TIMM13 deregulation could be one of causes underlying the disease phenotypes in GALK1 deficiency. Our study may expand the functional repertoire of GALK1 and provide new conceptual and therapeutic avenues for galactosemia and related disorders.

## 2 Results

### 2.1 GALK1 is Ubiquitously Expressed Across Human Tissues Regardless of Developmental Stages

To gain insight into the physiological roles of GALK1 beyond its phosphorylation of galactose, we assessed the tissue‐ and age‐specific expression patterns of GALK1 using publicly available RNA‐sequencing datasets. Analysis of GTEx data revealed that GALK1 is widely expressed across diverse adult tissues, with relatively low inter‐tissue variability (Figure 1A). GALK1 is notably expressed in non‐hepatic tissues with limited demand of galactose metabolism. On the other hand, since galactose is one of main energy sources for infants, GALK1 plays a critical role in life during the milk‐feeding period. After weaning period, the role of GALK1 in energy metabolism becomes inevitably reduced because milk is no more main food. To evaluate whether GALK1 expression is related with milk feeding, we further analyzed its expression in neonatal versus adult human tissues. Consequently, GALK1 levels in brain and skin tissues were not substantially different between infant and adult stages (Figure 1B,C). The constitutive and ubiquitous expression of GALK1 prompted us to hypothesize that GALK1 plays another role beyond galactose metabolism. To investigate this possibility, we explored gene ontology (GO) term associations of GALK1 expression across normal and tumor tissues using transcriptomic datasets from GTEx and TCGA with performing single‐sample Gene Set Enrichment Analysis (ssGSEA) and calculated the Spearman correlation coefficients between *GALK1* mRNA levels and pathway scores. Subsequently, three of the top 10 GO terms were identified to be positively related with mitochondrial biogenesis. The pathways are also related to RNA metabolism, apoptotic signaling, and pyridine‐containing compound metabolism (Figure 1D; Table S1).

**FIGURE 1:**
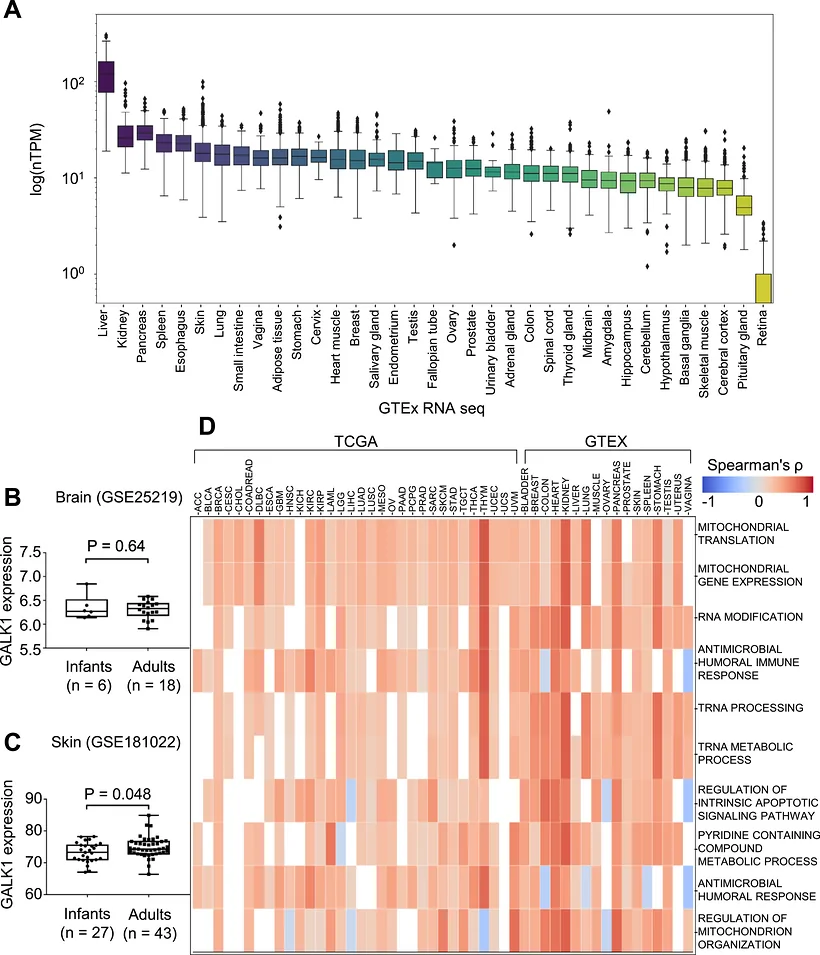
GALK1 expression is maintained across adult human tissues. (A) Broad expression profile of GALK1 across 35 adult human tissues from the GTEx RNA‐seq dataset (total n = 13 084 samples). Tissues are ordered from left to right by decreasing mean expression level, shown as normalized TPM (nTPM). In the boxplots, the central line indicates the median, the box represents the interquartile range (IQR), the whiskers extend to 1.5 times the IQR, and individual points denote outliers. (B) RNA‐seq data from the developmental transcriptome dataset (GSE25219) were analyzed across 16 brain regions, including cerebellar cortex, mediodorsal thalamus, striatum, amygdala, hippocampus, and 11 neocortical areas, in infants (≤ 1 year, n=6) and adults (> 18 years, n=18). (C) RNA‐seq dataset (GSE181022) was analyzed between infant (30.7 – 89.1 weeks, n=27) and adults (20 – 64 years, n=43) skins. Statistical significance was assessed by two‐tailed *t*‐test. (D) Correlation heatmap of GALK1 expression with ssGSEA score of Gene Ontology (GO) biological processes across TCGA and GTEx datasets. The color scale represents the Spearman's rank correlation coefficient (ρ), ranging from red (positive correlation, +1) to blue (negative correlation, ‐1). Non‐significant correlations (*p* > 0.05) are masked in white.

### 2.2 TIMM13 and PROSER2 Bind with GALK1 in Competition With Galactose

To examine a new function of GALK1 as a protein kinase, we searched for protein substrates to be phosphorylated by GALK1. Considering the reciprocal competition between different substrates in an enzymatic reaction, we hypothesized that the galactose substrate could interfere with the interaction between GALK1 and some protein substrates. We performed affinity purification followed by mass spectrometry (AP‐MS) in HEK293T cells transiently expressing FLAG/SBP double‐tagged GALK1 (F/S‐GALK1). To isolate the proteins to be phosphorylated by GALK1, cells were incubated with or without 10 mM galactose for 16 hr. The proteins whose binding with GALK1 were specifically inhibited by galactose were prioritized as candidate substrates for GALK1. The schematic workflow for GALK1 interactome profiling using AP‐MS experiment is shown in Figure 2A and the interacting proteins are listed in Table S2. In the intersection analysis of galactose‐sensitive interactors coprecipitated with either Flag or SBP tag, TIMM13 (mitochondrial import inner membrane translocase subunit Tim13) and PROSER2 (proline and serine‐rich protein 2) were identified as candidate substrates for GALK1. To confirm the protein interactions, we examined whether ectopically expressed proteins were associated with each other. In cross‐checked co‐IP using Ni‐NTA, anti‐Flag, or anti‐Myc beads, F/S‐TIMM13 and Myc‐PROSER2 both were identified to interact with His(6)‐GALK1 (Figure 2B,C). To validate the interactions between endogenous proteins, we performed co‐immunoprecipitation and Western blotting analyses. Either TIMM13 or PROSER2 was found to interact with GALK1 (Figure 2D,E).

**FIGURE 2:**
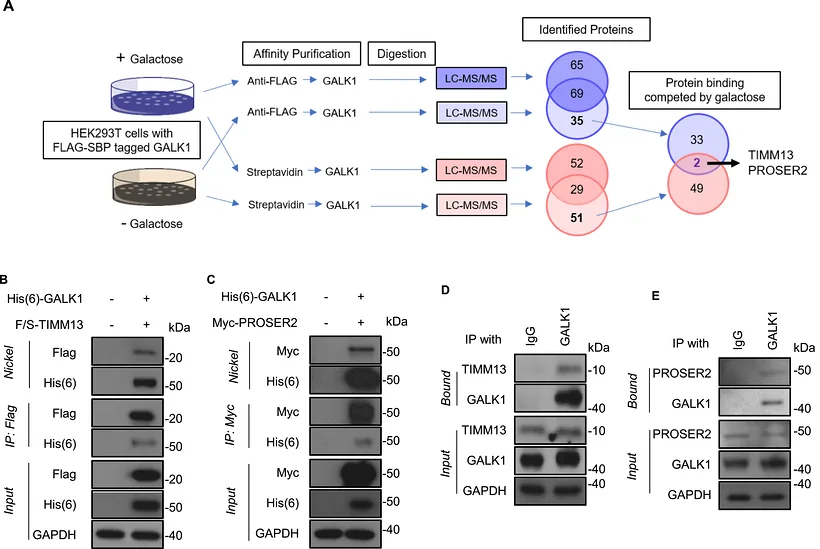
Identification and validation of TIMM13 as a GALK1‐interacting protein. (A) Schematic overview of the proteomic workflow to identify GALK1‐interacting proteins. HEK293T cells expressing F/S‐GALK1 were cultured for 16 h in the presence (+ galactose) or absence (‐ galactose) of 10 mM galactose. GALK1 complexes were purified in parallel using anti‐FLAG immunoprecipitation (blue) or streptavidin pull‐down (red). GALK1‐interacting proteins were identified by LC‐MS/MS. Venn diagrams show the overlap of proteins identified by LC‐MS/MS in each condition and show the overlap of proteins identified exclusively in the absence of galactose from both purification methods. Numbers indicate the count of identified proteins. (B,C) Reciprocal Co‐IP confirming the interaction between ectopically expressed proteins. His(6)‐GALK1 was pulled down to detect associated F/S‐TIMM13 (B) and Myc‐PROSER2 (C), and conversely, F/S‐TIMM13 and Myc‐PROSER2 were pulled down to detect His(6)‐GALK1. (D‐E) Co‐IP for validating the interaction between endogenous proteins. GALK1 was immunoprecipitated from cell lysates, followed by immunoblotting for the co‐precipitated TIMM13 (D) and PROSER2 (E). A non‐specific IgG was used as a negative control. Original blot can be found in Figure S13.

### 2.3 GALK1 Phosphorylates TIMM13, but Not PROSER2

To examine whether GALK1 phosphorylates its binding partners, we analyzed the phosphorylation of F/S‐TIMM13 and Myc‐PROSER2 using an antibody (p‐S/T/Y) detecting phosphorylated serine, threonine and tyrosine. Expressed TIMM13 was identified to be phosphorylated, which was markedly attenuated by GALK1 knockdown (Figure 3A). Furthermore, GALK1 overexpression robustly enhanced the phosphorylation of TIMM13 (Figure 3B). Using phospho‐Ser/Thr‐ and phospho‐Tyr‐specific antibodies, GALK1 was identified to phosphorylate only tyrosine residues (Figure S2A,B). In contrast, the phosphorylation of PROSER2 was not affected by GALK1 knockdown or over‐expression (Figure S1A–D). Of two GALK1‐interacting proteins, TIMM13 alone is likely to be phosphorylated GALK1‐dependently.

**FIGURE 3:**
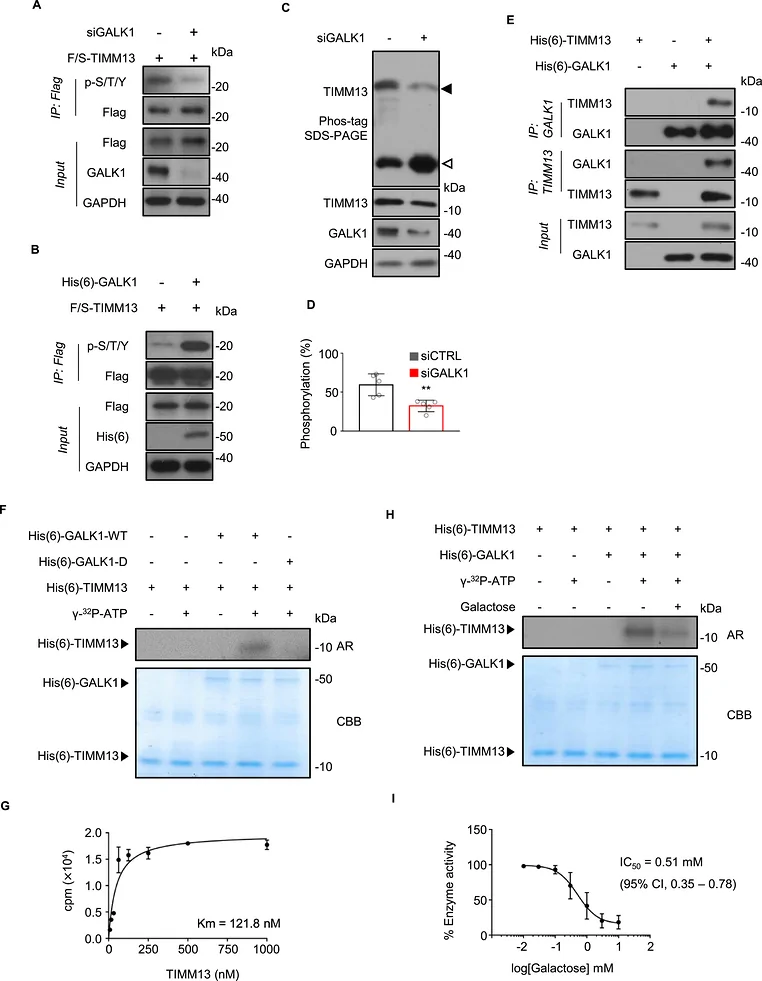
GALK1 is the protein kinase that phosphorylates TIMM13. (A,B) GALK1 regulates TIMM13 phosphorylation in HEK293T cells. Phosphorylation of immunoprecipitated F/S‐TIMM13, detected with a pan‐phospho antibody, was decreased upon GALK1 knockdown (A) and increased by His(6)‐GALK1 overexpression (B). (C,D) The phosphorylated fraction of endogenous TIMM13 is set by GALK1. Lysates of HEK293T cells transfected with control or GALK1‐targeting siRNA were resolved by Mn^2^^+^‐Phos‐tag SDS‐PAGE and immunoblotted with a phospho‐independent anti‐TIMM13 antibody. Closed and open arrowheads indicate the phosphorylated and non‐phosphorylated species, respectively. An identical sample set was resolved by conventional SDS‐PAGE to measure total TIMM13 (C). The phosphorylation ratio was calculated as the intensity of the upshifted band relative to the sum of both bands (D). Data are presented as the means ± SD (n = 5). Statistical significance was determined using unpaired two‐tailed *t* test. ** denotes *p* < 0.01. (E) In vitro interaction between GALK1 and TIMM13. Recombinant His(6)‐GALK1 and His(6)‐TIMM13 proteins were independently expressed and purified. They were put together in a test tube and then subjected to Co‐IP using antibodies against each protein. (F) GALK1 directly phosphorylates TIMM13. An in vitro kinase assay using 10 µCi γ‐[^3^ ^2^P]ATP demonstrates phosphorylation of recombinant His(6)‐TIMM13 by His(6)‐GALK1‐WT, but not a kinase‐dead mutant (His(6)‐GALK1‐D), as visualized by autoradiography (AR). Coomassie brilliant blue (CBB) staining confirms equal protein loading. (G) Quantification of phosphorylation (in counts per minute, CPM) was determined by liquid scintillation counter. Michaelis‐Menten kinetics of the phosphorylation were analyzed, and the Km for TIMM13 was determined by Lineweaver‐Burk plot analysis. Data are presented as the means ± SD (n = 3). (H) Galactose competes with TIMM13 for the kinase activity of GALK1. The in vitro kinase assay was performed with or without 1 mM galactose. Coomassie blue staining confirms equal protein loading. (I) Concentration dependence of galactose inhibition. The in vitro kinase assay was performed with galactose at the indicated concentrations, and the radioactivity incorporated was normalized to that of the galactose‐free reaction. The IC_50_ and its 95% confidence interval were obtained by non‐linear regression. Data are presented as the means ± SD (n = 5). Original blot can be found in Figure S13.

To confirm the phosphorylation of endogenous TIMM13, HEK293T homogenates were electrophoresed on conventional SDS‐PAGE and Mn^2^^+^‐Phos‐tag SDS‐PAGE, which were subjected to Western blotting with anti‐TIMM13 antibody. On the Phos‐tag gel, TIMM13 was shown as two species; the upper one is assigned to phosphorylated TIMM13 and the lower one to unphosphorylated TIMM13 (Figure 3C). A densitometry analysis revealed that approximately 60% of TIMM13 is phosphorylated (Figure 3D). GALK1 knock‐down reduced the upper band with a reciprocal increase in the lower band (Figure 3C,D), suggesting that endogenous TIMM13 is phosphorylated GALK1‐dependently. Under depletion of endogenous GALK1, the phosphorylated TIMM13 was restored by the ectopic expression of wild‐type GALK1, but not by an inactive GALK1 mutant (Figure S2C,D). Mn^2^^+^‐Phos‐tag SDS‐PAGE showed upshifted TIMM13 species in the cell‐free reaction mixture with wild‐type GALK1, but not in the mixture with an inactive mutant (Figure S2E). Collectively, these results further support our notion that GALK1 phosphorylates TIMM13.

To examine whether GALK1 acts as a protein kinase for TIMM13, we first examined the physical interaction between the two proteins in a cellular context. Using Förster resonance energy transfer (FRET) microscopy, we observed a significantly high normalized FRET (nFRET) signal in cells co‐expressing GALK1‐1‐192‐mTurquoise2 and TIMM13‐mCitrine (Figure S3A,B), suggesting that GALK1 interacts with TIMM13 within living cells. Next, we conducted an in vitro kinase assay with purified proteins. The substrate His(6)‐TIMM13 was purified from *E. coli* but the enzyme His(6)‐GALK1 was from HEK293T to ensure appropriate post‐translational folding for its enzymatic functionality. The two proteins were identified to be interacted physically in a cell‐free system (Figure 3E). In an in vitro kinase assay, TIMM13 was found to be directly phosphorylated by GALK1, but not by GALK1‐Dead whose G346, G347 and G349 residues were replaced with serine [16] (Figure 3F). Michaelis–Menten analysis showed that GALK1's Km for TIMM13 is 121.8 nM (Figure 3G). Given that GALK1's Km for galactose is estimated in a range from 0.1 mM to 1 mM, GALK1 seems to phosphorylate TIMM13 with relatively higher affinity [17, 18, 19, 20]. When 1 mM galactose was added to the kinase assay system, the GALK1‐mediated TIMM13 phosphorylation was noticeably inhibited (Figure 3H). IC_50_ of galactose against the GALK1 phosphorylation of TIMM13 was 0.51 mM (95% confidence interval, 0.35 – 0.78 mM) (Figure 3I). Collectively, TIMM13 is likely to be a bona fide substrate for GALK1, which expands the functional repertoire of GALK1 beyond carbohydrate metabolism.

### 2.4 GALK1 Phosphorylates TIMM13 at Tyrosine 73

To identify the phosphopeptides and phosphorylation site of TIMM13, we performed LC–MS/MS analysis under both in vitro and in vivo conditions (Figure 4A). For the in vitro assay, the recombinant TIMM13 protein was incubated with ATP in the presence or absence of purified GALK1 (Figure S4; Tables S3 and S4). In this system, the MS/MS analysis showed that the Y73 residue in TIMM13 was phosphorylated only in the presence of GALK1. For the in vivo assay, F/S–TIMM13 was expressed in HEK293T cells transfected with GALK1‐silencing siRNA or the GALK1‐expressing plasmid (Figure 4B; Tables S5 and S6). The MS/MS analysis identified Y73 phosphorylation under both conditions. The detection rate of the Y73‐phosphorylated peptide was markedly higher in GALK1‐expressing cells compared to GALK1‐knockdown cells (Table S5). The S7 residue, which is previously reported to be phosphorylated [21], was also identified to be phosphorylated. Unlike pY73, however, this site was supported by a single peptide‐spectrum match in each condition, and its signal was below the threshold for reliable quantification in GALK1‐expressing cells (Table S5). These data cannot draw any conclusion as to whether S7 phosphorylation responds to GALK1, so this phosphorylation was not further investigated. To examine whether or not Y73 is the only one site targeted by GALK1, we expressed F/S‐TIMM13‐Y73F lacking Y73 in HEK293T cells and found that no signal for tyrosine phosphorylation was detected in Y73F mutant regardless of GALK1 expression (Figure 4C). The same result was obtained with purified proteins, in which HEK293T‐derived GALK1 incorporated ^3^ ^2^P into recombinant wild‐type TIMM13 but not to Y73F mutant (Figure S5A). This GALK1‐dependent phosphorylation was also detected in the cell‐free kinase reaction products by Mn^2^^+^‐Phos‐tag SDS‐PAGE. After incubated with recombinant GALK1, the wild‐type His(6)‐TIMM13, but not the Y73F mutant, was shifted up on the gel (Figure S5B). To rule out the non‐specific reactions of other kinases co‐purified with GALK1, we performed the in vitro kinase assay using the human GALK1 recombinant protein expressed in *E. coli*. The recombinant protein could phosphorylate wild‐type TIMM13 but not Y73F (Figure S5C). This result indicates that the GALK1‐mediated Tyr phosphorylation occurs only at Y73 residue. To confirm the GALK1‐dependent Y73 phosphorylation of endogenous TIMM13, we generated a specific antibody against Y73‐phosphorylated TIMM13 using a synthetic peptide harboring phosphorylated Y73 as an epitope. Immunoblotting analysis with this antibody clearly showed the Y73 phosphorylation of endogenous TIMM13 in HEK293T cells, and the signal was markedly increased by pervanadate, a tyrosine phosphatase inhibitor (Figure S5D). The antibody gave a signal with wild‐type TIMM13 and active GALK1, but not with the Y73F mutant or with inactive GALK1 (Figure S5E). The Y73 phosphorylation was markedly reduced by GALK1‐silencing shRNA targeting GALK1 3’UTR. Under knock‐down of endogenous GALK1, the ectopic expression of GALK1 fully recovered the Y73 phosphorylation, but that of an inactive GALK1 mutant failed to do (Figure 4D). We next examined whether galactose interferes with this reaction in a cellular context. When HEK293T cells were incubated with galactose (10 and 25 mM) for 24 h, the Y73 phosphorylation of endogenous TIMM13 was noticeably abolished (Figure S5F). Under the same condition, intracellular galactose reached approximately 0.7 mM, while it was undetectable in cells without galactose supplementation (Figure S5G). Consistently, in a cell‐free system with purified proteins, galactose inhibited the interaction between TIMM13 and GALK1, whereas glucose did not (Figure S5H). Galactose is likely to compete with TIMM13 for binding to GALK1 and by doing so it inhibits the GALK1 reaction for TIMM13 phosphorylation.

**FIGURE 4:**
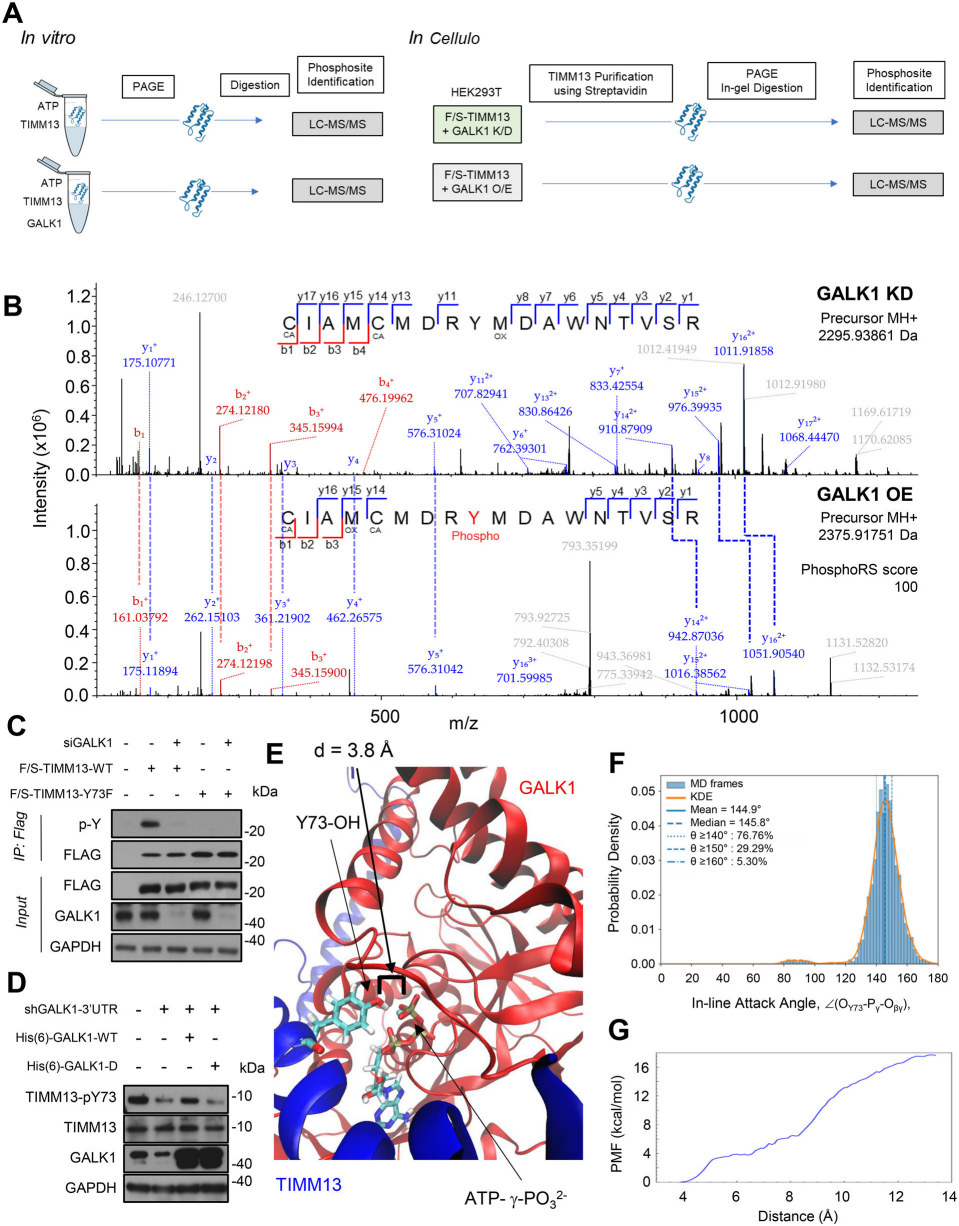
GALK1 phosphorylates TIMM13 at Tyrosine 73. (A) Schematic overview of the phosphoproteomics workflow. In vitro and in vivo experiments were performed under various conditions to identify phosphopeptides and their corresponding phosphosites. For each experimental condition, excised gel bands of TIMM13 were subjected to in‐gel digestion. The resulting peptides were analyzed by high‐resolution LC‐MS/MS using data‐dependent acquisition (DDA). Phosphopeptides and phosphosites in TIMM13 were identified and localized using a sequence database‐based search strategy equipped with the PhosphoRS node in Proteome Discoverer. This figure was created with BioRender.com. Abbreviations: rTIMM13, recombinant TIMM13; F/S, FLAG/streptavidin‐binding peptide; LC‐MS/MS, liquid chromatography‐tandem mass spectrometry. (B) Representative LC‐MS/MS spectra of a TIMM13‐derived peptide (CIAMCMDRYMDAWNTVSR) identified from F/S‐TIMM13‐transfected HEK293T cells. The top panel shows the MS/MS spectrum of the unmodified peptide from siGALK1 co‐transfected cells, while the bottom panel corresponds to the peptide phosphorylated at residue Tyrosine 73 (pY73) from His(6)‐GALK1 co‐transfected cells. All fragment ion matches along the peptide sequence and the corresponding precursor ion information are also presented. The PhosphoRS site‐localization score for the phosphorylated peptide is also provided in the bottom panel. Matched *b* ions are shown in red and *y* ions in blue. Unshifted fragments are indicated by dotted lines (light red for b ions and light blue for y ions), while shifted m/z values are specifically highlighted with dotted blue lines for y ions. The peptide contains a missed cleavage at Arg72, which lies in an Asp‐Arg motif that is cleaved inefficiently by trypsin irrespective of phosphorylation status. Abbreviation: LC‐MS, liquid chromatography‐mass spectrometry; CA, carbamidomethylation; OX, oxidation; Phospho, phosphorylation. (C) Immunoblot analysis of anti‐FLAG precipitates from HEK293T cells transfected with wild‐type (WT) or Y73F‐mutated F/S‐TIMM13 with or without siGALK1. Phosphorylation was detected using a pan‐phosphotyrosine (p‐Y) antibody. (D) Immunoblotting analysis of lysates from HEK293T cells co‐transfected with an shRNA targeting the 3'UTR of *GALK1* mRNA and a plasmid for shRNA‐resistant His(6)‐GALK1‐WT or kinase‐dead mutant (His(6)‐GALK1‐D). Endogenous TIMM13 phosphorylation at Y73 was detected with specific anti‐phospho‐TIMM13 Y73 antibody. (E) 3D structure of human TIMM13 bound to human GALK1 in the presence of ATP and Mg. TIMM13 and GALK1 are shown as a ribbon, with the exception of TIMM13‐Y73. TIMM13‐Y73 and ATP are shown as sticks and Mg is shown as a ball (cyan). The hydroxyl group of Y73 and the γPO3 of ATP are indicated. Original blot can be found in Figure S13. (F) In‐line attack‐angle distribution in the distance‐conditioned GALK1–TIMM13–MgATP complex. The in‐line attack angle was defined as ∠(O_Y73_‐P_γ_‐O_βγ_), where O_Y73_ is the phenolic oxygen of TIMM13 Y73 and O_βγ_ is the β‐γ bridging oxygen of ATP. During the 20‐ns production simulation, a harmonic restraint was applied only to the O_Y73_‐P_γ_ distance, centered at 3.8 Å, and no angular restraint was imposed. Bars show the normalized histogram of MD frames, and the solid curve represents a kernel‐density estimate (KDE). The mean and median angles were 144.9° and 145.8°, respectively. Fractions of frames with angles ≥140°, ≥150°, and ≥160° were 76.8%, 29.3%, and 5.3%, respectively. The indicated angular thresholds are descriptive bins used to characterize the distribution and are not treated as strict catalytic cutoffs. (G) Potential of mean force (PMF) profiles, derived from the Jarzynski equality, as a function of the distance between the oxygen of Y73‐OH and the phosphorus of ATP‐γPO3 for TIMM13/GALK1/ATP.

### 2.5 TIMM13 Engages the Catalytic Site of GALK1 in Molecular Modeling

To understand the molecular structure for GALK1‐TIMM13 interaction, we performed in silico protein‐protein docking simulations using the ZDOCK server. TIMM13 was modeled in its reduced state because TIMM13 does not form disulfide bonds in the cytoplasm where GALK1 is present [22, 23]. The resulting model predicts a plausible binding interface between the two proteins (Figure 4E). Notably, the model shows that TIMM13 docks the catalytic pocket of GALK1 in presence of ATP and Mg^2+^. Predicted distance between phosphorus in γ‐phosphate of ATP and oxygen in the hydroxyl group of TIMM13 Y73 was 3.8 Å, which was comparable with the distance (3.3 Å – 3.8 Å) between phosphorus in the gamma phosphate of ATP and oxygen in the hydroxyl group of galactose C1 in crystal structure of human galactokinase complex (Figure S6) [24]. Because donor–acceptor distance alone does not define a productive phosphotransfer geometry, we further checked the in‐line attack angle, ∠(O_Y73_–P_γ_–O_βγ_), where O_βγ_ is the β‐γ bridging oxygen of ATP. The angle was analyzed in a distance‐conditioned MD ensemble in which only the O_Y73_–P_γ_ distance was harmonically restrained near 3.8 Å, with no angular restraint imposed. The angular distribution showed that the median angle was 145.8° and angles of ≥ 160° accounted for 5.3% of the sampled frames (Figure 4F). Given that the 161° near‐attack geometry was reported for a PKA reactant‐state mimic containing intact ATP and a Cys‐substituted pseudo‐substrate [25, 26], 5.3% population having ≥160° attack angle could participate in the kinase reaction. The kinase reference geometry supports the structural accessibility of a plausible pre‐reactive configuration of the GALK1‐TIMM13 complex. To quantitatively assess the thermodynamic stability of the predicted GALK1‐TIMM13 binding model, we calculated the potential of mean force (PMF) using steered molecular dynamics (SMD) simulations in conjunction with the Jarzynski equality (Figure 4G). The PMF profile was generated along a defined reaction coordinate: the distance between phosphorus in γ‐phosphate of ATP and oxygen in the hydroxyl group of TIMM13 Y73. This distance was progressively increased from an initial bound state of ∼4 Å to a dissociated state at 13 Å. The resulting PMF profile illustrates the unbinding process. The snapshot at ∼4 Å, corresponding to the free energy minimum, represents the stable, pre‐phosphorylation complex. Along with increasing distance, the free energy rose until it plateaus, indicating the fully dissociated state around 13 Å. From this profile, the binding free energy difference (ΔGbind​) between the bound and dissociated states, which is defined as the debinding energy, is calculated to be approximately 18 kcal/mol (Figure 4G). This substantial energy barrier suggests a thermodynamically stable interaction between TIMM13 Y73 and the catalytic site of GALK1, a prerequisite for an efficient phosphorylation event.

### 2.6 GALK1‐Mediated TIMM13 Phosphorylation Occurs in the Cytoplasm and This Is Critical for Mitochondrial Respiration

Consistent with previous reports identifying TIMM13 as an essential mitochondrial chaperone, our single‐sample gene set enrichment analysis (ssGSEA) of TCGA and GTEX datasets revealed a robust correlation between TIMM13 expression and gene sets governing mitochondrial functions (Figure S7; Table S7). To investigate the physiological consequences of the GALK1‐TIMM13 interaction, we assessed cellular bioenergetics using Seahorse XF Mito Stress Assays [14]. Consequently, TIMM13 knock‐down suppressed the mitochondrial respiratory function, which was estimated based on basal respiration, ATP production, and maximal respiratory capacity. The suppressed respiratory function was almost recovered by the restoration of wild‐type TIMM13. However, the respiratory recovery by TIMM13 was abolished by GALK1 knock‐down (Figure 5A). This respiratory defect was accompanied by a marked decrease in mitochondrial membrane potential, which was rescued by wild‐type TIMM13 re‐expression, whereas mitochondrial mass remained largely unaltered (Figure S8A,B). This result suggests that TIMM13 functions to regulate mitochondrial respiration strictly depending on GALK1. To further examine the role of GALK1 in mitochondrial respiration, we evaluated the mitochondrial function in GALK1‐knocked‐down cells. The GALK1 knock‐down reduced all respiratory parameters, but the restoration of GALK1 recovered the mitochondrial respiration almost completely. However, GALK1 restoration failed to rescue mitochondrial respiration under TIMM13 knock‐down (Figure 5B). Consistently, GALK1 depletion caused a reduction in mitochondrial membrane potential that was rescued by GALK1 only in the presence of TIMM13, while mitochondrial mass remained constant (Figure S8C,D). In this reciprocal design, TIMM13 or GALK1 was depleted and then restored with an shRNA‐resistant construct, which was challenged by depletion of its partner. Consequently, GALK1 and TIMM13 act sequentially in a signaling pathway rather than in parallel, and play a crucial role in the mitochondrial respiration.

**FIGURE 5:**
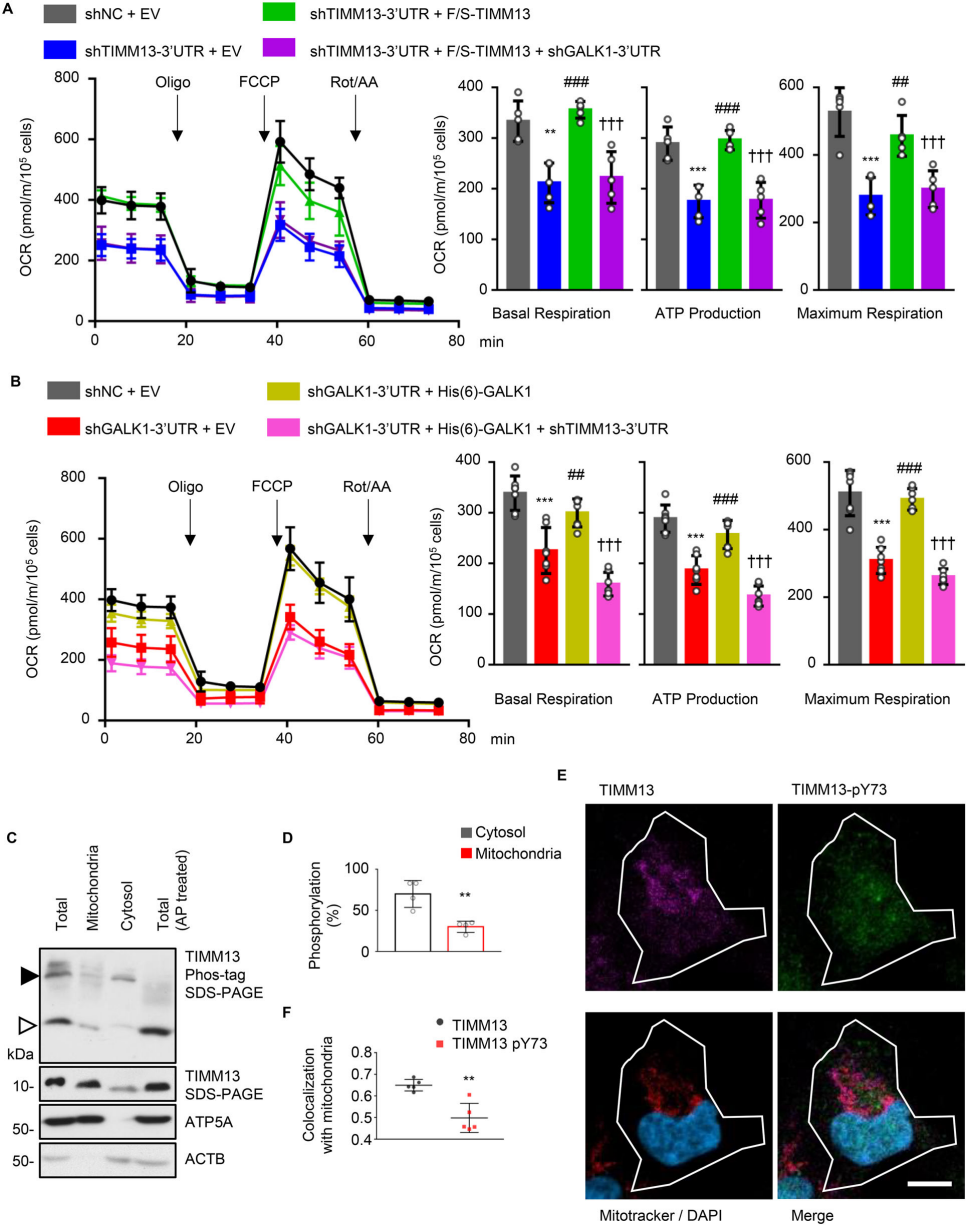
GALK1‐mediated TIMM13 phosphorylation is essential for mitochondrial respiration and occurs on the cytosolic pool of TIMM13. The mitochondrial respiration was assessed based on the oxygen consumption rate (OCR) using a Seahorse XF Analyzer. The OCR profiles (top panels) illustrate the cellular response to a mitochondrial stress test, involving sequential injections of oligomycin (1.5 µM), the uncoupling agent FCCP (1.0 µM), and a combination of rotenone and antimycin A (0.5 µM each). Bar graphs (bottom panels) quantify basal respiration, ATP production, and maximum respiration from the profiles. (A) HEK293A cells were transfected with control (shNC + EV), shTIMM13‐3'UTR + EV, shTIMM13‐3'UTR + F/S‐TIMM13 (rescue), or shTIMM13‐3'UTR + F/S‐TIMM13 + shGALK1‐3'UTR. Asterisks(*) indicate the comparison versus the shNC + EV control group, hash symbols(#) versus the shTIMM13‐3'UTR + EV knockdown group, and daggers(†) versus the shTIMM13‐3'UTR + F/S‐TIMM13 rescue group (n = 5 per group). (B) HEK293A cells were transfected with control (shNC + EV), shGALK1‐3'UTR + EV, shGALK1‐3'UTR + His(6)‐GALK1 (rescue), or shGALK1‐3'UTR + His(6)‐GALK1 + shTIMM13‐3'UTR. Asterisks(*) indicate the comparison versus the shNC + EV control group, hash symbols(#) versus the shGALK1‐3'UTR + EV knockdown group, and daggers(†) versus the shGALK1‐3'UTR + His(6)‐GALK1 rescue group (n = 7 per group). All data are presented as mean ± SD from a minimum of five biologically independent replicates with three to six technical replicates. Statistical significance for comparisons was determined using unpaired two‐tailed t test. Double symbols denote *p* < 0.01 and triple symbols *p* < 0.001. (C‐D) Y73‐phosphorylated TIMM13 is enriched in the cytosol. Mitochondrial and cytosolic fractions were prepared from HEK293T cells and resolved by Mn^2^^+^‐Phos‐tag SDS‐PAGE followed by immunoblotting with a phospho‐independent anti‐TIMM13 antibody. Closed and open arrowheads indicate the phosphorylated and non‐phosphorylated species, respectively. A total lysate treated with alkaline phosphatase is included to confirm the assignment, an identical sample set was resolved by conventional SDS‐PAGE to measure total TIMM13, and fraction purity was verified with ATP5A and ACTB (C). The phosphorylation ratio in each fraction was calculated as the intensity of the upshifted band relative to the sum of both bands (D). Original blot can be found in Figure S13. (E,F) Representative confocal immunofluorescence images of HEK293T cells co‐stained for total TIMM13 (magenta) and Y73‐phosphorylated TIMM13 (green), with mitochondria labeled by MitoTracker (red) and nuclei by DAPI (blue). The outline denotes the cell boundary. Scale bar, 10 µm (E). Colocalization with mitochondria was quantified as Manders' coefficient M1, calculated separately for each signal (F). Data in (D) and (F) are presented as the means ± SD (n = 5). Statistical significance was determined using unpaired two‐tailed *t* test. ** denotes *p* < 0.01.

GALK1 is a cytosolic enzyme, whereas mature TIMM13 resides in the mitochondrial intermembrane space. For the differences in subcellular localization, we had a question about the location for TIMM13 phosphorylation. Mitochondrial and cytosolic fractions were prepared from HEK293T cells and resolved by Mn^2^^+^‐Phos‐tag SDS‐PAGE. The upshifted species was abolished by pre‐treatment of the lysate with alkaline phosphatase, and the purity of the fractions was confirmed with ATP5A and ACTB (Figure 5C). The phosphorylated form of TIMM13 was enriched in the cytosol, accounting for approximately 70% of TIMM13 in the cytosolic fraction against approximately 30% in the mitochondrial fraction (Figure 5D). We next examined the distribution of the modified protein in situ. Fixed cells were co‐stained with anti‐pY73 antibody, anti‐TIMM13 antibody, and MitoTracker. As expected, total TIMM13 showed a predominantly mitochondrial distribution. However, the pY73 signal was significantly less colocalized with mitochondria (Figure 5E,F). These results indicate that Y73‐phosphorylated TIMM13 is mainly present in the cytoplasm, whereas mature TIMM13 in the dephosphorylated status is enriched in the mitochondria.

### 2.7 Y73 Phosphorylation Sets the Structure of TIMM13 and Ensures Zn^2+^ Binding to Prevent Premature Protein Oxidation

Based on a computational analysis of TIMM13 structure, Y73 appears to be located in proximity to positively charged residues R41 and K45 (Figure 6A) [27, 28]. When Y73 is phosphorylated, the PO_3_^2^^−^ group could electrostatically interact with the NH_3_^+^ groups at R41 and K45, probably setting the conformation of TIMM13 (Figure 6B). To estimate the stability of TIMM13 conformation, we analyzed molecular dynamics simulations to calculate the probability of the distances between Y73 and R41/K45. In case of unphosphorylated Y73, the distances are widely distributed without structural convergence (Figure 6C,D, blue dots), suggesting that TIMM13 is hardly set as a stable conformation. In contrast, when Y73 is phosphorylated, Y73‐R41 and the Y73‐K45 distances both are presented as two big peaks at less than 1 nm (Figure 6C,D, red dots). These results indicate that phosphorylated Y73 could set TIMM13 as relatively stable conformation. Therefore, Y73 phosphorylation is likely to contribute to a stable conformation of TIMM13.

**FIGURE 6:**
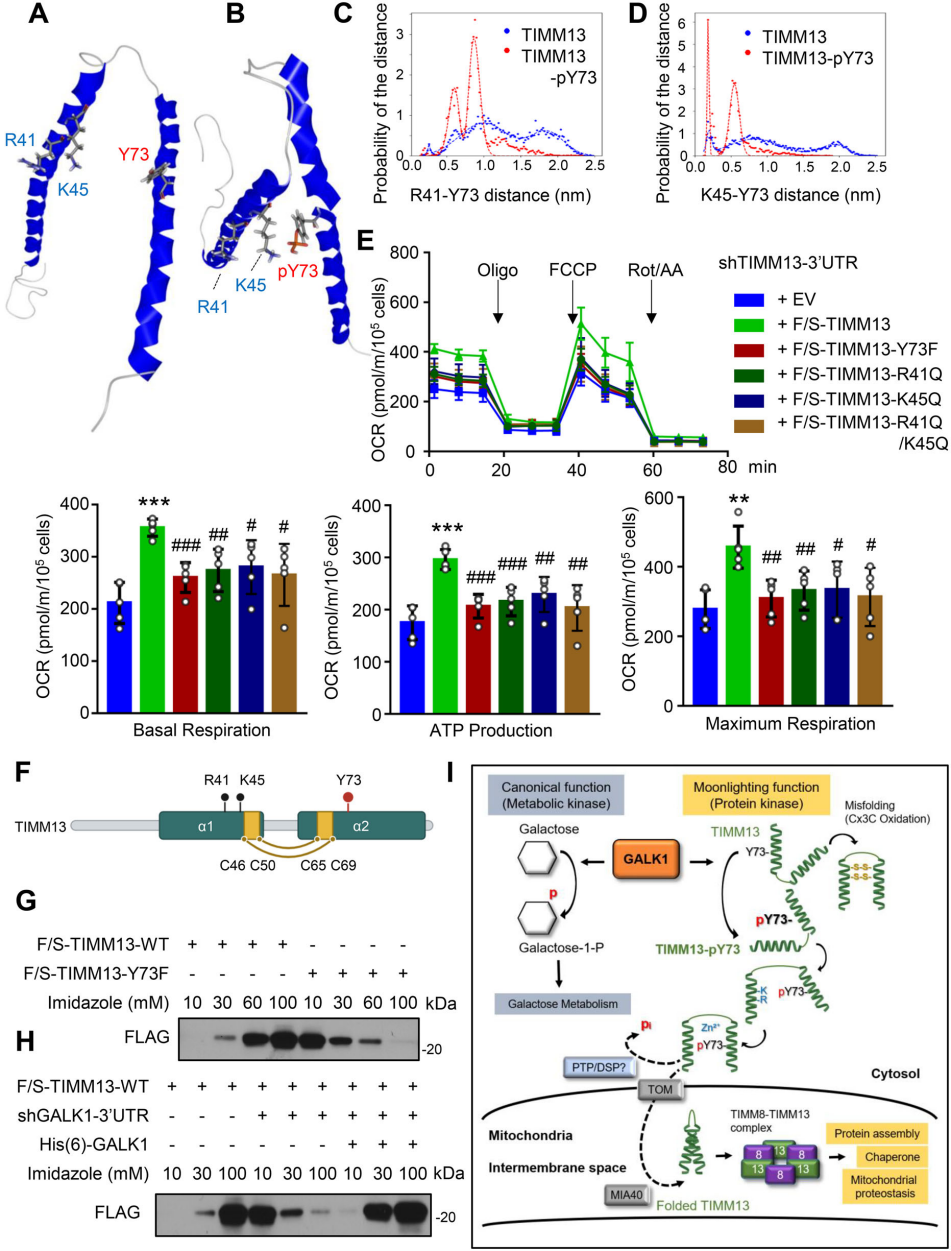
Y73 phosphorylation of TIMM13 sets the protein structure and ensures the binding of zinc ion. (A,B) Estimated 3D conformations of human TIMM13. MD simulations were performed under reducing conditions without disulfide bonds. The models depict the non‐phosphorylated (Y73) state (A) and the phosphorylated (pY73) state (B). Conserved Arg41 (R41) and Lys45 (K45) residues electrostatically interact with pY73 (dashed lines). (C‐D) Probability distribution plots of the distance between Y73 and R41 (C) or K45 (D) by MD simulations. The distance was measured between the hydroxyl oxygen at Y73 (or a phosphate oxygen at pY73) and the terminal nitrogen atoms at R41 and K45 side chains. Scatter points correspond to the raw data from the simulation trajectories, while dashed lines represent multi‐component Gaussian mixture model fits. (E) OCR was measured in TIMM13 knockdown cells co‐expressing an empty vector (EV), shRNA‐resistant F/S‐TIMM13, or the indicated shRNA‐resistant F/S‐TIMM13 point mutants (Y73F, R41Q, K45Q, R41Q/K45Q) (n=5 per group). Asterisks(*) and hash symbols(#) indicate the comparison to the shTIMM13‐3'UTR + EV knockdown group and to the shTIMM13‐3'UTR + F/S‐TIMM13 rescue group, respectively. * and ^#^, *p* < 0.05; ** and ^##^, *p* < 0.01; *** and ^###^, *p* < 0.001. (F) Schematic representation of the TIMM13 protein, showing the two α‐helices, the twin CX_3_C motifs, and the positions of R41, K45, and Y73. (G‐H) Zinc‐binding affinity of TIMM13 was assessed using Zn^2^^+^ affinity beads. HEK293T cells were subjected to expression of F/S‐TIMM13 or Y73F (G), or to GALK1 knockdown or overexpression (H). Resin‐bound proteins were sequentially eluted with increasing concentrations (10 to 100 mM) of imidazole. Original blot can be found in Figure S13. (I) Proposed role of GALK1‐mediated Y73 phosphorylation in the mitochondrial translocation of TIMM13.

We next examined whether the electrostatic interaction is essential for the TIMM13 function. Under depletion of endogenous TIMM13, wild‐type or mutated TIMM13 was ectopically expressed in HEK293T cells. The suppressed mitochondrial respiration was recovered by wild‐type TIMM13, but not by TIMM13 Y73F which is not phosphorylated by GALK1. Similarly, the TIMM13 mutants R41Q, K45Q, and R41Q/K45Q, which were designed to disrupt the electrostatic interaction with phosphorylated Y73, also failed to recover mitochondrial respiration (Figure 6E). In line with these respiratory defects, all the tested mutants failed to rescue the mitochondrial membrane potential, whereas mitochondrial mass remained unaffected (Figure S8E,F). The efficiency of TIMM13 knockdown and expression was verified by immunoblotting (Figure S8G). These findings support a role for Y73 and R41/K45 in TIMM13‐dependent mitochondrial respiration, consistent with the predicted pY73–R41/K45 interaction. These data uncover a novel GALK1‐TIMM13 axis to determine the rate of mitochondrial respiration.

It has been well known that Zn^2^^+^ binding to the twin “CX_3_C” zinc finger motif in small TIM proteins prevents premature oxidative folding the proteins in the cytoplasm [29, 30]. The structure of TIMM13 is illustrated in Figure 6F. Moreover, the phosphorylation‐dependent enhancement of metal binding affinity is an emerging regulatory mechanism in protein homeostasis [31, 32]. We here hypothesized that the phosphorylation at Y73 facilitates the Zn^2^^+^ coordination to TIMM13, and performed an immobilized Zn^2^^+^ affinity chromatography with step‐wise imidazole elution. Phosphorylatable TIMM13‐WT was eluted by 100 mM imidazole, but unphosphorylatable TIMM13‐Y73F was done by 10 mM imidazole (Figure 6G and Figure S9A). Furthermore, the Zn^2^^+^‐binding capacity of TIMM13 was diminished by GALK1 knock‐down, which was reversed by GALK1 restoration (Figure 6H and Figure S9B). These data strongly indicate that GALK1‐dependent Y73 phosphorylation is critical for Zn^2^^+^ binding to TIMM13. Given that the Zn^2^^+^ binding inhibits the oxidative folding of TIMM proteins [28, 29], we tested the possibility that Y73 phosphorylation prevents the oxidative change in TIMM13. The oxidative folding of small TIM proteins is monitored by intrinsic tryptophan fluorescence, and human TIMM13 contains a single native tryptophan, W77, which is close to Y73 [28, 30]. In both WT and Y73F TIMM13, the peak of fluorescence emission spectrum was shifted to ∼350 nm under full oxidation (Figure S9D,E). However, W77A TIMM13 showed no difference in the fluorescence spectrum between oxidized and reduced forms (Figure S9C), verifying that the W77 residue contributes to the redox spectrum shift. According to the spectral changes, we evaluated the degree of oxidative folding in TIMM13 by analyzing the fluorescence intensity at 350 nm. Consequently, Zn^2^^+^ strictly suppresses the oxidative folding of TIMM13. Conversely, the disruption of Y73 phosphorylation—either via Y73F mutation (Figure S9F) or GALK1 knockdown (Figure S9G)—abrogated this Zn^2^^+^‐mediated inhibition of premature oxidative folding. Since TIMM13 with oxidative folding cannot enter into the mitochondrion, we examined whether the pY73‐mediated electrostatic interaction is a prerequisite for its mitochondrial translocation. Immunofluorescence analyses revealed that the mitochondrial localization of TIMM13 was significantly reduced by GALK1 knock‐down. Even in the presence of GALK1, the Y73F mutant and the electrostatic interaction‐defective mutants (R41Q, K45Q) exhibited compromised mitochondrial localization (Figure S10A,B). Since TIMM13 and TIMM8A form the chaperone complex in the intermembrane space, we examined whether Y73 phosphorylation affects their association. When His(6)‐TIMM8A was pulled down from HEK293T cells, F/S‐TIMM13‐WT was efficiently co‐precipitated but Y73F mutant was recovered a little (Figure S10C). Consistently, GALK1 knock‐down reduced the interaction between Flag‐TIMM8A and F/S‐TIMM13 (Figure S10D). Together, these data suggest that Zn^2^^+^ binding following Y73 phosphorylation may precede the mitochondrial localization and TIMM8A association of TIMM13. They support a model in which Y73 phosphorylation limits premature oxidative folding before mitochondrial import, which is summarized in Figure 6I.

### 2.8 Deregulation of Y73 Phosphorylation and Mitochondrial Respiration in Galactosemia‐Associated GALK1 Mutations

Loss‐of‐function mutations in *GALK1* cause Type II galactosemia, an autosomal recessive disorder characterized by the accumulation of galactose and galactitol. Given the putative role of the GALK1‐TIMM13 axis in mitochondrial respiration, we hypothesized that pathogenic *GALK1* mutations might disrupt this regulatory axis, potentially contributing to the complicated symptoms beyond canonical metabolic defects. To test this, we selected three representative pathogenic variants—P28T, L356P and L139P—from the ClinVar database based on their unambiguous classification. We first examined whether these mutations compromise the GALK1‐TIMM13 axis. In HEK293T cells depleted of endogenous GALK1, reconstitution with P28T, L356P mutants failed to restore TIMM13 phosphorylation to levels observed with wild‐type GALK1 (Figure 7A). We next investigated the mechanisms underlying these signaling failures. Co‐immunoprecipitation assays revealed that the P28T and L356P mutants display significantly attenuated binding affinities for TIMM13 compared to wild‐type GALK1 (Figure 7B). Given a previous reports demonstration that some pathogenic mutations impair the solubility of GALK1, we checked this possibility in the expressed GALK1 mutants. Consequently, both P28T and L356P existed predominantly in the insoluble fraction (Figure 7C) and also could not pass through the 200 kDa cut‐off membrane (Figure 7D). These results clearly showed that P28T and L356P become insoluble due to self‐aggregation [16, 33]. Confocal microscopy further corroborated this, showing distinct intracellular puncta for P28T and L356P, in contrast to the diffuse cytoplasmic distribution of WT GALK1 (Figure S11A). In contrast, the L139P variant did not aggregate but displayed a catalytic defect; in vitro kinase assays demonstrated a marked reduction in its ability to phosphorylate TIMM13 at Y73 (Figure S11A–C). Collectively, these results indicate that P28T, L139P, and L356P mutations deregulate the GALK1‐TIMM13 axis through either protein aggregation or catalytic inactivation. Consequently, we assessed the physiological impact of these defects on mitochondrial function. Interestingly, A198V, which is the most frequent *GALK1* variant in Korean and Japanese populations [18, 34, 35, 36], showed a diffuse cytoplasmic distribution without puncta (Figure S11D), and catalyzed the Y73 phosphorylation of TIMM13 as efficiently as wild‐type GALK1 (Figure S11E). Although A198V induces galactosemia due to its limited action on galactose metabolism, it is expected to have a lesser impact on mitochondrial respiration [16]. We utilized the Hep3B hepatic model because the liver, the predominant tissue of GALK1 and TIMM13 expression, is the primary site of galactose metabolism and the core pathology of galactosemia, including liver damage and hepatosplenomegaly. Compared with wild‐type GALK1, none of the pathogenic GALK1 variants was able to restore the mitochondrial respiration in Hep3B cells (Figure 7E and Figure S12). This suggests that mitochondrial dysfunction resulting from a disrupted GALK1‐TIMM13 axis may be a critical, yet previously overlooked, molecular feature of Type II galactosemia.

**FIGURE 7:**
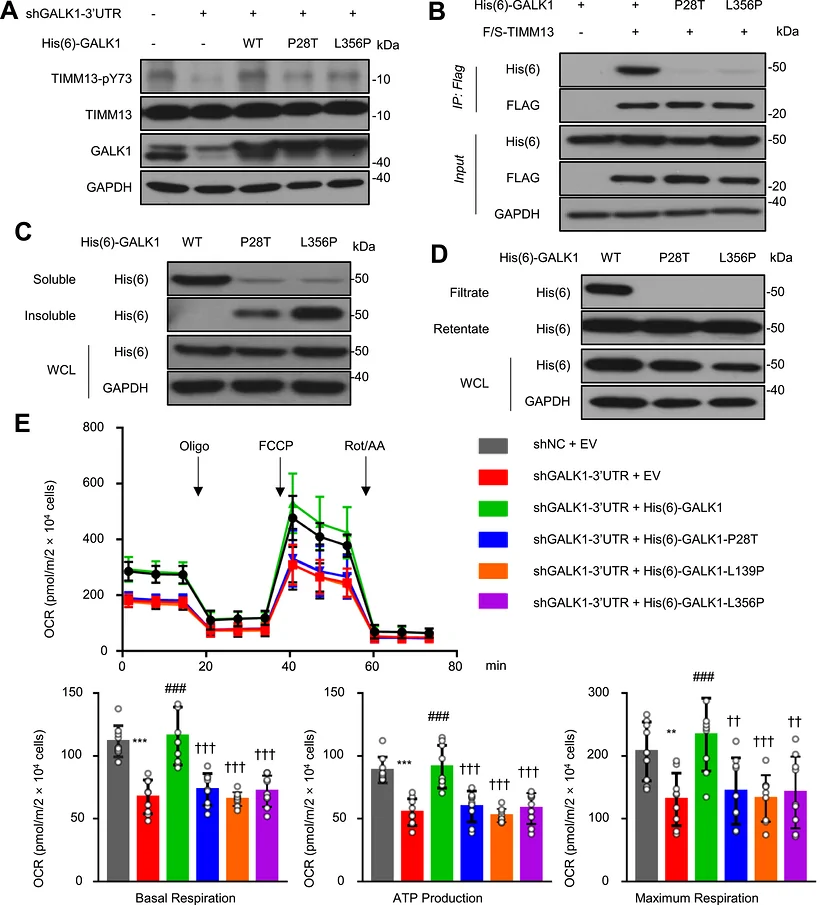
Galactosemia‐associated GALK1 mutants poorly phosphorylate TIMM13 at Y73 and lower the rate of mitochondrial respiration. (A) HEK293T cells were co‐transfected with an shRNA targeting the 3'UTR of *GALK1* mRNA and a plasmid expressing wild type His(6)‐GALK1 or galactosemia‐associated His(6)‐GALK1 (P28T and L356P). Y73 phosphorylation of endogenous TIMM13 was detected with anti‐Y73 phosphoryated TIMM13 antibody. (B) The interaction between F/S‐TIMM13 and wild type or mutated GALK1. In HEK293T cell lysates, F/S‐TIMM13 was pulled down using anti‐FLAG beads, and the TIMM13‐bound GALK1 was detected by immunoblotting with an anti‐His(6) antibody. (C) The solubilities of wild‐type, P28T, and L356P GALK1 proteins in HEK293T cells. Cell lysates were fractionated into soluble and insoluble fractions through sequential lysis. The soluble fraction was extracted with a buffer containing Triton X‐100, and the remaining pellet was subsequently lysed with an SDS‐containing buffer to yield the insoluble fraction. Proteins from each fraction, along with whole cell lysates (WCL), were subjected to SDS‐PAGE and immunoblotted with an anti‐His(6) antibody to detect GALK1. GAPDH was used as a loading control for the WCL samples. (D) Identification of protein aggregation using a filtration assay. Lysates from HEK293T cells expressing His(6)‐tagged WT, P28T, or L356P GALK1 were passed through a 200 kDa cutoff filter. The flow‐through (filtrate) and the filter‐retained (retentate) fractions were analyzed by immunoblotting with an anti‐His(6) antibody. GAPDH in the whole cell lysate (WCL) served as a loading control. Original blot can be found in Figure S13. (E) The mitochondrial respiration in Hep3B was assessed based on OCR. OCR profile (top) and quantification (bottom) of basal respiration, ATP production, and maximum respiration in cells transfected with control (shNC + EV), shGALK1‐3'UTR + EV, shGALK1‐3'UTR + His(6)‐GALK1 (rescue), shGALK1‐3'UTR + His(6)‐GALK1‐P28T, shGALK1‐3'UTR + His(6)‐GALK1‐L139P or shGALK1‐3'UTR + His(6)‐GALK1‐L356P. Asterisks(*) indicate the comparison versus the shNC + EV control group, hash symbols(#) versus the shGALK1‐3'UTR + EV knockdown group, and daggers(†) versus the shGALK1‐3'UTR + His(6)‐GALK1 rescue group (n = 9 per group). All data are presented as mean ± SD. Double symbols denote *p* < 0.01 and triple symbols *p* < 0.001.

## 3 Discussion

This study was originally designed to test the possibility that GALK1 plays a moonlighting job as a protein kinase beyond the phosphorylation of galactose. TIMM13, which plays essential roles in the mitochondrial respiration, was identified as a substrate of GALK1. GALK1 phosphorylates TIMM13 at Y73 in the cytoplasm, and the phosphorylated TIMM13 is held in the reduced state by electrostatic bridges with two cationic residues and Zn^2^^+^ binding. The TIMM13 phosphorylation was shown to positively regulate mitochondrial respiration. Notably, the phosphorylated site is highly conserved across species, underscoring its functional importance. This discovery delineates a novel signaling pathway ‘GALK1‐TIMM13 axis’ that provides a potential link between galactose metabolism and mitochondrial respiration.

From a clinical perspective, this discovery provides new insight into the symptoms manifesting in patients with the type II galactosemia (GALK1 deficiency). The main symptoms of this disease are juvenile cataract and pseudotumor cerebri, both of which are largely attributed to galactitol accumulation and subsequently increased osmotic pressure in the eye lens and the cerebrospinal fluid, respectively. In addition, this disease is accompanied with other symptoms, such as delayed neurodevelopment, hearing loss, disturbances in glucose and cholesterol metabolism, hepatomegaly, and ovarian failure. It's still unclear whether the latter symptoms are directly linked to impaired galactose metabolism [3, 37]. This study may provide a clue to understanding of relationship between GALK1 deficiency and the unexplainable symptoms. We propose that the clinical manifestations of GALK1 deficiency are partly attributed to deregulated mitochondrial respiration due to insufficient phosphorylation of TIMM13.

The Y73 phosphorylation‐dependent stabilization of TIMM13 can be contextualized in the biogenesis pathways of small Tim proteins. TIMM13 maturation is basically dependent on the redox state. TIMM13 lacking mitochondrial targeting signal is imported through the translocase of the outer membrane (TOM) complex in a reduced state, and in turn undergoes oxidative folding in the intermembranous space via the Erv1–Mia40 disulfide relay system. This protein modification culminates in the formation of two intramolecular disulfide bonds within the ‘CX_3_C’ motif, which also constitute the targeting determinant retaining the protein in the intermembranous space [22, 23]. These bonds determine the alpha‐helical ‘Tim fold’, which is essential for the assembly of the TIMM13‐TIMM8 hexamer [28]. We propose that the GALK1‐mediated Y73 phosphorylation is a preceding step to ensure the intramolecular S‐S bond formation in a right orientation. Consistent with this, the upper band of TIMM13 on Phos‐tag gel was enriched in the cytosolic fraction, consistent with phosphorylation in the pre‐import pool of TIMM13. The pY73‐R41 and ‐K45 ionic bonds may stabilize a monomeric TIMM13 in the cytoplasm before its entry into the intermembranous space. Moreover, in our experimental settings, Y73 phosphorylation was associated with enhanced Zn^2^^+^ binding and reduced premature oxidative folding of TIMM13. Together, these findings support a model in which GALK1‐mediated Y73 phosphorylation acts as a cytosolic pre‐import event that may maintain TIMM13 in a conformation competent for mitochondrial import. Whether this mechanism directly determines the import competence of newly synthesized TIMM13 remains to be examined. This may be the very case that the cytosolic event of post‐translational modification determines the mitochondrial import of a precursor protein. More broadly, post‐translational modifications can regulate the intracellular localization and function of nuclear‐encoded mitochondrial proteins. In mitochondrial ribosomal proteins, phosphorylation and acetylation have been associated with altered intracellular localization and changes in mitochondrial respiration and oxidative phosphorylation [38]. Our findings extend this framework by identifying GALK1‐mediated Y73 phosphorylation of TIMM13 as a regulatory link between a cytosolic phosphorylation event, mitochondrial protein import, and respiratory function.

Protein phosphorylation is generally counterbalanced by phosphatases. In the present study, pervanadate increased the pY73‐TIMM13 signal, consistent with dynamic turnover of this modification in cells. Because pervanadate inhibits phosphatases that depend on an active‐site cysteine, this response is compatible with the involvement of an as‐yet‐unidentified PTP/DSP‐family phosphatase in pY73‐TIMM13 dephosphorylation. However, the pervanadate experiment does not identify the relevant enzyme or establish its site of action. Although pY73‐TIMM13 is enriched in the cytosolic fraction, the present data do not resolve whether dephosphorylation occurs before mitochondrial import, during import, or after mitochondrial entry. Identifying the responsible phosphatase and defining its compartment will be necessary to establish the spatiotemporal regulation of TIMM13 Y73 phosphorylation.

Our data show that galactose inhibits GALK1‐mediated TIMM13 Y73 phosphorylation under in vitro and cell‐culture conditions. This role is supported by comparing the IC_50_ (0.51 mM) of galactose for TIMM13 phosphorylation with its intracellular concentration (0.7 mM) following a 10 mM galactose treatment. A galactose concentration of 10 mM appears sufficient to inhibit TIMM13 phosphorylation under cell culture conditions. Indeed, blood galactose concentrations have been reported to reach approximately 9 mM in untreated patients with GALK1 deficiency. However, this finding does not necessarily indicate that TIMM13 phosphorylation is attenuated in patient tissues, as intracellular galactose concentrations in these patients have not yet been determined [3, 39, 40].

Terminal differentiation entails a shift in mitochondrial function toward effective energy generation. In stem and progenitor cells, however, mitochondria undergo a metabolic reprogramming to cope with extreme proliferation [41, 42, 43, 44, 45]. An aerobic glycolysis, which is characterized as increased glycolysis but mitochondrial suppression, can provide intermediate metabolites to be used as building blocks for *de novo* syntheses of nucleic acids, non‐essential amino acids, and lipids [46, 47]. Whether dietary galactose modulates the GALK1–TIMM13 axis during development remains to be determined.

In many clinical studies, diverse missense mutations of the *GALK1* gene have been identified. In the present study, we examined the impacts of P28T and L356P mutations, which manifest more severe symptoms than other types of type II galactosemia, on the GALK1‐TIMM13 axis. Consequently, P28T and L356P mutations were shown to deregulate the GALK1‐TIMM13 axis. These GALK1 mutants could not sufficiently phosphorylate TIMM13 and the ectopic expression of them failed to rescue the mitochondrial respiration suppressed by knock‐down of endogenous GALK1. To understand why these mutants have lower activity on TIMM13 phosphorylation, we first assumed the possibility that the mutations distort the structure of the catalytic sites for ATP and TIMM13 binding. According to our molecular dynamics analysis, however, these mutations were unlikely to directly affect the catalytic reaction because the positions of the mutated amino acids are far away from the catalytic sites. Unexpectedly, we observed that these mutants had a high tendency to be aggregated in cells. The aggregation of the enzyme might lower the catalytic activity because it hinders the physical contact between enzyme and substrate. The aggregation of mutated GALK1s is not surprising because there are many cases supporting that some proteins get aggregated by a single missense mutation. For examples, α‐synuclein (A53T), DJ‐1 (L166P), and SOD1 (A4V or G93A) mutations have been reported to induce protein aggregation by altering the thermodynamic stability and native conformation of the proteins [48, 49, 50]. However, the molecular dynamics analysis regarding the mutation‐induced protein aggregation is not investigated in the present study.

The phenotypes and frequencies of galactosemia‐associated GALK1 mutations are diverse: GALK1 P28T variant is highly enriched in the Romani population (carrier frequency ∼1:47) [51]. Its incidence is about 1:40 000 in regional newborn screening and 1:30 000 in global allele frequencies (ExAC) [3, 52]. It drives a severe, early‐onset syndromic phenotype encompassing bilateral cataracts, microcephaly, and intellectual disability. GALK1 L139P and L356P variants exhibit lower global frequencies: 1:150 000 ∼ 300 000 in GnomAD for L139P and 1:250 000 in TOPMed for L356P [53, 54]. They manifest autosomal recessive congenital cataracts but significant extraocular symptoms have not been reported yet. For a better understanding of the pathogenic impacts mediated by GALK1‐TIMM13 interactions, future investigations remain to be done to identify separation‐of‐function variants among the other reported *GALK1* mutants [18, 35, 36, 52, 53, 54, 55].

In conclusion, GALK1 could be characterized as a multifaceted enzyme that participates in carbohydrate metabolism via galactose phosphorylation and also in regulation of mitochondrial respiration by phosphorylating TIMM13. GALK1 is likely to act as a galactose sensor to regulate mitochondrial function, and TIMM13 seems to be a signaling mediator in the GALK1's action. From a clinical perspective, the discovery of the GALK1‐TIMM13 regulatory axis may provide deeper insight into the etiology of diet‐refractory morbidities in Type II galactosemia.

### 3.1 Limitations of the Study

Although PROSER2 interacted with GALK1 galactose‐dependently, this protein was not shown to be phosphorylated by GALK1 in our experimental settings. Nonetheless, this result alone cannot exclude the possibility that PROSER2 is one of GALK1's substrates because the phosphorylation could occur depending on cell context or require some unique co‐factors [56]. Alternatively, even if PROSER2 is not a catalytic substrate, it could regulate the GALK1‐TIMM13 reaction. In this assumption, PROSER2 may act as a regulatory subunit of protein complexes or an allosteric modulator, thereby influencing GALK1 activity toward other downstream targets. However, we did not investigate the functional consequence of PROSER2 interaction, which is a limitation of the present study.

## 4 Methods

### 4.1 Cell Lines and Cell Culture Conditions

HEK293T was obtained from American Type Culture Collection (ATCC; Manassas, VA); Hep3B from Korean Cell Line Bank (KCLB; Seoul, Korea). HEK293A was kindly provided by Dr. Jihye Seong (Seoul National University College of Medicine). These cell lines were cultured in Dulbecco's Modified Eagle Medium (WelGENE LM001‐05: Daegu, Korea) supplemented with 10% heat‐inactivated fetal bovine serum (GenDEPOT F0900‐050: Barker, TX, USA) and 100 units of penicillin and 100 µg/mL streptomycin (Gibco 15140122: Waltham, MA, USA) under 5% CO_2_ at 37°C. Mycoplasma contamination was routinely checked when cell growth or shape was altered.

### 4.2 Antibodies and Reagents

Flag‐tag (F7425; 1:5000 dilution) and phospho‐tyrosine antibodies (05‐321; 1:1000) were purchased from Sigma‐Aldrich (St. Louis, MO, USA); GAPDH (2118S; 1:50 000) and Myc‐tag (2276S; 1:2000) antibodies from Cell Signaling Technology (Danvers, MA, USA); PROSER2 (sc‐398975; 1:1000), β‐tubulin (sc‐5274; 1:1000), ATP5A (sc‐136178; 1:1000), and β‐actin (sc‐47778; 1:1000) antibodies from Santa Cruz Biotechnology (Santa Cruz, CA, USA); GALK1 (NBP2‐16591; 1:2000) antibody from Novus Biologicals (Littleton, CO, USA); TIMM13 (11973‐1‐AP; 1:2000) antibody from Proteintech (Wuhan, China); His‐tag (D291‐3; 1:5000) antibody from MBL (Nagoya, Japan). Pan‐phospho antibody (61‐8300; 1:1000), phospho‐Ser/Thr antibody (PA5‐121314; 1:1000), HRP‐conjugated goat anti‐rabbit IgG (G21234; 1:5000), HRP‐conjugated goat anti‐mouse IgG (G21040; 1:5000), and HRP‐conjugated goat anti‐human IgG (31412; 1:5000) were purchased from Invitrogen (Carlsbad, CA, USA). A monoclonal antibody against Y73‐phosphorylated TIMM13 (1:1000) was raised using a phage display technology provided by Bioneer (Daejeon, South Korea). The synthetic peptides IAMCMDRYMDAWNTV and IAMCMDR(p)YMDAWNTV were obtained from Bioneer and conjugated with bovine serum albumin to be used as antigens. VeriBlot for IP Detection Reagent (ab131366 and ab131368) were purchased from Abcam (Cambridge, UK). Other chemicals were obtained from Sigma‐Aldrich.

### 4.3 DNA Construction and Mutagenesis

Human GALK1 (NM_000154), TIMM13 (NM_012458), TIMM8A (NM_004085) and PROSER2 (NM_153256) cDNAs were cloned using RT‐PCR with Pfu DNA polymerase and inserted into His(6)‐tagged pcDNA, Flag‐SBP (Streptavidin‐Binding Peptide)‐tagged pcDNA, myc‐tagged pcDNA or pET28 plasmid. Arg41, Lys45, Tyr73, and Trp77 in TIMM13 were substituted with Phe, Gln, or Ala using PCR‐based mutagenesis, which were designated as TIMM13‐R41Q, K45Q, R41Q/K45Q, Y73F, and W77A. Pro28, Leu139, Ala198 or Leu356 in GALK1 were substituted with Thr (GALK1‐P28T) [16, 24, 57, 58, 59, 60], Val (GALK1‐A198V) or Pro (GALK1‐L139P and GALK1‐L356P) [59, 61, 62], respectively. To inactivate GALK1, the conserved glycine residues at Gly346, Gly347, and Gly349 were substituted with serine, which was designated as GALK1‐Dead [16, 24, 34, 58]. To analyze FRET between GALK1 and TIMM13, GALK1 (amino acids 1–192) and TIMM13 cDNAs were inserted into the mTurquoise2‐containing vector MB‐115 and the mCitrine‐containing vector MB‐118. The plasmids for FRET were kindly given by Dr. Jihye Seong. All DNA constructs were verified by DNA sequencing.

### 4.4 Transfection of DNAs and RNAs

For transient expression or knockdown, cells at ∼70% confluence were transfected using jetPRIME (for plasmid) or Lipofectamine RNAiMAX (for siRNA). Transfected cells were stabilized for 24 or 48 h, or for other durations as indicated, before being used in experiments. To enable rescue experiments, the shRNA was designed to target the 3' untranslated region (3'UTR). To rescue the knockdown phenotype, cells were co‐transfected with this 3'UTR‐targeting shRNA plasmid and an shRNA‐resistant expression vector. Oligonucleotides were annealed and inserted into the shRNA expression vectors pGPU6/Neo and pGPU6/RFP/Neo (GenePharma: Shanghai, China). The shRNA sequences are as follows: Negative control (shNC), TTCTCCGAACGTGTCACGT; shGALK1, GTGCTCATCACCAACTCTAAT; shGALK1‐3’UTR, TGTGAGGCACCCCCAGGACAG; shTIMM13‐3’UTR: CTCCTGCGGATGGGCAAATAA. The GALK1‐targeting siRNA (5’‐CUCACUCAGAGACGACUAU‐3’) and the Control siRNA were purchased from Bioneer.

### 4.5 Immunoblotting and Immunoprecipitation

Cell lysates were prepared in a lysis buffer (150 mM Tris‐HCl, pH 6.8, 20% Glycerol, 5% BME, 4% SDS). Cocktails of protease inhibitors (Sigma‐Aldrich: P2714) or phosphatase inhibitors (GeneDepot: P3200) were added to the lysis buffer when needed. Proteins were separated on SDS/PAGE and transferred to Immobilon‐P membranes (Millipore: IPVH08100, Burlington, MA, USA). The membranes were blocked with 5% skim milk or 5% bovine serum albumin (Sigma‐Aldrich: A3059 for anti‐phosphorylation antibody), incubated overnight at 4°C with a primary antibody, incubated with a horseradish peroxidase (HRP)‐conjugated secondary antibody for 1 h, and visualized using the Luminata Crescendo Western HRP substrate (Millipore: WBLUR0500) or ECL Select Western Blotting Detection Reagent (Amersham Biosciences: RPN2235, Piscataway, NJ). To inhibit cellular protein tyrosine phosphatases, cells were treated with 100 µM pervanadate, freshly prepared by mixing sodium orthovanadate with hydrogen peroxide, or with vehicle for 15 min before harvest. To analyze protein interactions, cell lysates were prepared in an immunoprecipitation buffer (1% NP‐40, 10% Glycerol, 50 mM Tris, 0.15 M NaCl, 2.5 mM EDTA) containing a protease inhibitor cocktail or in a Ni‐affinity pulldown buffer (1% NP‐40, 10% Glycerol, 50 mM Tris, 0.15 M NaCl, 10 mM Imidazole) containing an EDTA‐free protease inhibitor cocktail (Thermo Fisher Scientific: 87785, Waltham, MA, USA) and a phosphatase inhibitor cocktail. Cell lysates were incubated with a primary antibody or IgG overnight at 4°C, and the immune complexes were pulled down with protein A/G beads. Otherwise, cell lysates were incubated with Nickel affinity beads (Qiagen: 1018244, Hilden, Germany), anti‐FLAG beads (Millipore: A2220), or streptavidin beads (Millipore: S1638) at 4°C for 4 h. The bound proteins were eluted in a denaturing SDS sample buffer or with Flag‐tag peptide, and subjected to SDS‐PAGE. All experiments were performed three or more times.

### 4.6 Phos‐tag SDS‐PAGE

Cell lysates were resolved on 15% polyacrylamide gels containing 50 µM Phos‐tag acrylamide (FUJIFILM Wako: AAL‐107, Osaka, Japan) and 100 µM MnCl_2_, which were prepared on the day of use. The gels were washed in a transfer buffer containing 1 mM EDTA and then in the EDTA‐free transfer buffer, and proteins were transferred onto PVDF membranes and immunoblotted with an anti‐TIMM13 antibody [63]. An identical set of samples was resolved by conventional SDS‐PAGE in parallel to measure total TIMM13. For dephosphorylation, lysates were prepared in the immunoprecipitation buffer lacking phosphatase inhibitors, incubated with calf intestinal alkaline phosphatase (New England Biolabs: M0290, Ipswich, MA, USA) at 1 unit per 5 µg protein for 60 min at 37°C, and then mixed with the SDS sample buffer. The phosphorylation ratio was calculated using ImageJ as the intensity of the upshifted band relative to the sum of both bands.

### 4.7 Subcellular Fractionation

Mitochondrial and cytosolic fractions were prepared from HEK293T cells using the Mitochondria Isolation Kit for Cultured Cells (Thermo Fisher Scientific: 89874) with Dounce homogenization according to the manufacturer's instructions, and the post‐mitochondrial supernatant was clarified at 12 000 × g for 10 min at 4°C to obtain the cytosolic fraction. Fraction purity was verified by immunoblotting for ATP5A (a mitochondrial marker) and ACTB (a cytosolic marker).

### 4.8 LC‐MS Analysis to Identify Interacting Proteins

Bound proteins obtained from affinity purification were eluted using a protein extraction buffer containing 4% SDS, 0.1 M Tris (pH 7.5), and 2 mM Tris(2‐carboxyethyl)phosphine (TCEP; Thermo Fisher Scientific, #77720). Protein concentrations were determined using BCA (Thermo Fisher Scientific: 23225). Protein digestion was carried out according to a Filter‐Aided Sample Preparation protocol. Peptides were analyzed on the NanoLC system (Thermo Fisher Scientific: Easy‐nLC 1000) equipped with a trap column (5 µm C18, 100 µm i.d., 2 cm length) and an analytical column (2 µm C18, 50 µm i.d., 15 cm length). The chromatographic separation was performed using a linear gradient from 10% to 32% acetonitrile containing 0.1% formic acid at a flow rate of 300 nL/min for 85 min, followed by a gradient ramp from 32% to 40% over the next 5 min. Ionized peptides were analyzed on a quadrupole‐orbitrap mass spectrometer (Thermo Fisher Scientific: Q‐Exactive). Data‐dependent acquisition mode was used to select the top 20 most intense precursor ions from each MS1 scan, acquired at a resolution of 70 000 and with an automatic gain control (AGC) target of 3 × 10^6^, over an m/z scan range of 300–1800. Fragmentation was performed using stepped higher‐energy collisional dissociation with normalized collision energies of 27, 30, and 33. MS2 spectra were collected at a resolution of 17 500 with an AGC target of 2 × 10^5^. Raw LC‐MS data were processed in Proteome Discoverer (v2.5, Thermo Fisher Scientific) using the SEQUEST search engine to match spectra against the UniProtKB human protein database (74 540 entries, June 2020 release) [64, 65]. The sequence database search was conducted using the following parameters: tryptic peptides allowing up to two missed cleavages, peptides with a minimum length of six amino acids, a precursor mass tolerance of 20 ppm, and a fragment mass tolerance of 0.02 Da. Carbamidomethylation of cysteine was set as a static modification, and variable modifications included N‐terminal acetylation and methionine oxidation. Search results were filtered at the peptide‐spectrum match and protein levels using a 1% false discovery rate. Chromatographic alignment was refined using the “Feature Mapper” node with a maximum retention time shift of 20 min and a mass tolerance of 10 ppm. Protein quantification was based on precursor ion intensities derived from peptide spectrum matches, peptides, and protein groups, and normalization was performed using the “total peptide amount” option in the “Precursor Ions Quantifier” node.

### 4.9 LC‐MS Analysis to Identify Post‐Translational Modifications

The proteins in a gel slice on SDS‐PAGE were digested with trypsin. Peptides were analyzed using a Vanquish Neo UHPLC system (Thermo Fisher Scientific: VN‐S10‐A‐01) equipped with the same trap and analytical columns described above. The LC gradient began with 0% to 4.6% acetonitrile with 0.1% formic acid over 14.5 min at a flow rate of 300 nL/min, which was then decreased to 100 nL/min over 1 min. This was followed by a linear increase from 4.6% to 28% acetonitrile over 127.5 min, and from 28% to 35% over an additional 15 min. MS analysis was performed on an Orbitrap Exploris 480 instrument (Thermo Fisher Scientific: BRE725539). MS1 scans were acquired in DDA mode with a resolution of 120 000, AGC target of 3 × 10^6^, and an m/z range of 300–1800. For MS/MS acquisition, precursor ions were fragmented using stepped HCD with normalized collision energies of 24, 27, and 30, and analyzed at a resolution of 30 000 with an AGC target of 3 × 10^5^. LC‐MS raw data were processed using Proteome Discoverer with the SEQUEST search engine. Search parameters included tryptic peptides with at least six amino acids, up to two missed cleavages, a precursor mass tolerance of 20 ppm, and a fragment mass tolerance of 0.02 Da. Carbamidomethylation of cysteine was set as a static modification, while dynamic modifications included N‐terminal acetylation, methionine oxidation, and phosphorylation (+79.966 Da). Peptide and protein identifications were filtered using a 1% FDR threshold. Phosphorylation site localization was assessed by using the “PhosphoRS Mode” in the IMP‐ptmRS node in the processing step and activating the “Modification Sites” node in the consensus step.

### 4.10 Preparation of Recombinant Proteins

Recombinant His(6)‐TIMM13 protein was expressed in *Escherichia coli* BL21. BL21 cells were lysed using a buffer containing 50 mM NaH_2_PO_4_, 300 mM NaCl, 10 mM imidazole, 0.1% Triton X‐100, 0.5% glycerol, 400 µM PMSF, and 1 mM DTT. His(6)‐GALK1 protein was expressed in HEK293T and extracted in NP‐40 lysis buffer containing 1% NP‐40, 10% glycerol, 50 mM Tris‐HCl pH 7.5, 150 mM NaCl, 10 mM imidazole, and EDTA‐free protease inhibitor cocktail, phosphatase inhibitor cocktail, followed by sonication to achieve homogenization. Proteins from both lysates were pulled down using nickel‐NTA affinity beads and eluted with 250 mM imidazole. The amounts and purities of the eluted proteins were assessed by SDS‐PAGE followed by staining with EZ‐Gel Staining Solution (DoGen Bio, Seoul, Korea). Recombinant human GALK1 (M1–L392) expressed in *Escherichia coli* was purchased from MedChemExpress (HY‐P70365: Monmouth Junction, NJ, USA).

### 4.11 In Vitro Binding Assay

Purified His(6)‐TIMM13 (1 µg) and His(6)‐GALK1 (1 µg) were co‐incubated in 200 µL of a binding buffer (25 mM HEPES/pH 7.5, 150 mM KCl, 12.5 mM MgCl_2_, 0.5 mM DTT, 0.1% NP‐40, and 10% glycerol) at 4°C for 1 h and further incubated with anti‐GALK1 or anti‐TIMM13 antibody at 4°C for 1 h. For competition experiments, His(6)‐GALK1 was preincubated with galactose (0.1, 1, or 10 mM) or glucose (10 mM) in the binding buffer at 4°C for 30 min before the addition of His(6)‐TIMM13, and the sugar was maintained during the subsequent co‐incubation and antibody incubation. After the beads were washed with the binding buffer, bound proteins were eluted in the denaturing SDS sample buffer and subjected to Western blotting.

### 4.12 Measurement of Intracellular Galactose

HEK293T cells were incubated with or without 10 mM galactose for 24 h and washed with ice‐cold PBS to remove extracellular galactose. Cells (1 × 10^6^) were lysed and deproteinized using a 3‐kDa cut‐off spin filter, and galactose in the filtrate was quantified using a fluorometric galactose assay kit (Abcam: ab83382) according to the manufacturer's instructions. Intracellular concentrations were calculated by dividing the measured amount by the packed cell volume determined in parallel from an equal number of cells. Values below the lowest standard were regarded as not detected.

### 4.13 In Vitro Kinase Assay and Kinetic Parameters for the Phosphorylation

Ten ng of His(6)‐GALK1 (or‐GALK1‐Dead) and 500 ng of His(6)‐TIMM13 were co‐incubated in 30 µL of a kinase buffer containing 20 mM HEPES (pH 7.5), 60 mM NaCl, 50 mM MgCl_2_, 1 mM DTT, 200 µM ATP, and a phosphatase inhibitor cocktail in the presence or absence of galactose. The reactions were carried out at room temperature for 1 h and terminated by heating the samples in SDS sample buffer. Protein phosphorylation was assessed by Western blotting. Where indicated, His(6)‐GALK1 purified from HEK293T cells was replaced with 10 ng of recombinant human GALK1 expressed in *E. coli*, and His(6)‐TIMM13‐Y73F was used in place of the wild‐type substrate. These reactions were supplemented with 10 µCi of [γ‐^3^ ^2^P]‐ATP, and ^3^ ^2^P incorporation was visualized by autoradiography after SDS‐PAGE, with equal protein loading confirmed by Coomassie brilliant blue staining. To analyze the kinetic parameters for TIMM13 phosphorylation, 10 ng of His(6)‐GALK1 and various amounts of His(6)‐TIMM13 were incubated in 51 µL of a kinase buffer containing phosphatase inhibitor cocktail, 200 µM ATP, and 10 µCi of [γ‐^32^P]‐ATP. The reactions were incubated at 25°C for 10 min, and terminated by adding ice‐cold trichloroacetic acid (finally 10%). Precipitated proteins were spun down at 15 000 × g, and washed three times in ice‐cold acetone. The radioactivity in the pellet was measured using MicroBeta2 scintillation counter (Revvity: Waltham, MA, USA) with Ultima Gold LSC Cocktail (Sigma: L8286). The *K*_m_ of GALK1 for TIMM13 was calculated from a plot of 1/c.p.m. versus 1/[TIMM13] according to the Lineweaver–Burk equation. Data represented the means from three independent experiments. To determine the IC_50_ of galactose, reactions were performed as described for the kinetic analysis with 500 ng of His(6)‐TIMM13 and galactose at concentrations ranging from 0.01 to 100 mM. The radioactivity incorporated at each concentration was normalized to that of the galactose‐free reaction, and the IC_50_ and its 95% confidence interval were obtained by non‐linear regression of log(inhibitor) versus normalized response using GraphPad Prism.

### 4.14 Data Preparation (Tcga, Ssgsea, Geo)

To identify key pathways in association with GALK1, we analyzed GTEx mRNA expression data (https://www.gtexportal.org/) from 16 major normal tissues [66]. The GTEx data used in this study were obtained from version 10 dataset released on 2022‐06‐06. TCGA mRNA expression data (cancer data) were obtained from cBioPortal (https://www.cbioportal.org/) [67, 68]. In this study, colon adenocarcinoma and rectum adenocarcinoma data were combined into the colorectal adenocarcinoma data (COADREAD). Gene signature data were retrieved from the Molecular Signatures Database (MSigDB; https://www.gsea‐msigdb.org/), including 7608 gene sets from the GO Biological Process ontology [69, 70]. GO Biological Process gene sets provide functional annotations for molecular activities and biological pathways. To investigate the developmental regulation of GALK1, age‐dependent transcriptomic data were sourced from the NCBI Gene Expression Omnibus (GEO; https://www.ncbi.nlm.nih.gov/geo/). For the analysis of postnatal brain development, data were utilized from GSE25219 which contains data from 16 brain regions [71]. To compare the data between infants (birth to 1 year, n = 6) and adults (over 18 years, n = 18), the mean value of GALK1 levels in 16 brain regions of each individual was evaluated as the representative GALK1 level in the entire brain. For the analysis of skin, data were obtained from GSE181022, which analyzed the transcriptomes in infant (30.7 – 89.1 weeks, n = 27) and adult (25 of 20 twenties and 18 of sixties) skins [72].

### 4.15 Single‐Sample Gene Set Enrichment Analysis

RNA expression data were normalized using the TPM (Transcripts Per Million) method. Single‐sample Gene Set Enrichment Analysis was performed using the ssGSEA module (version 10.1.0) in GenePattern 2.0 (https://genepattern.org/) with default parameters, except for the "combine mode," which was set to separate _UP and _DN gene sets [73]. ssGSEA computes an enrichment score for each gene set within a single sample by ranking all genes. Unlike traditional GSEA, ssGSEA allows for pathway activity quantification at the single‐sample level, enabling the identification of sample‐specific patterns in pathway activation associated with GALK1 expression [74]. Gene signature data were retrieved from MSigDB v2024.1. The relationship between ssGSEA enrichment scores and GALK1 mRNA expression was evaluated by computing Spearman rank correlation coefficients (ρ) across multiple human tissue types, using transcriptomic data from the GTEx and TCGA cohorts. To integrate correlation signals across tissues, we employed a meta‐analytical framework based on Fisher's Z‐transformation and sample‐size‐based weighting [75, 76]. This approach allowed us to compute a weighted meta‐correlation score (Z meta) for each gene set, reflecting both the strength and the reliability of correlation signals across tissues. Each Spearman correlation coefficient *ρi ​* for tissue i was first transformed using Fisher's Z‐transformation:

$$Z_{i}=\frac{1}{2}\ln\left(\frac{1+\rho_{i}}{1-\rho_{i}}\right)$$

Weights were defined as *wi* = *Ni* ‐ 3, where *Ni* ​ is the number of samples in tissue *i*. This weighting reflects the inverse variance of the Fisher's Z distribution, ensuring that tissues with larger sample sizes contribute more robustly to the final summary measure. The weighted meta‐correlation score was then computed as:

$$Z_{meta}=\left(\frac{\sum_{i=1}^{k}w_{i}Z_{i}}{\sum_{i=1}^{k}w_{i}}\right)$$

where *k* is the number of tissues analyzed. This formulation estimates the average correlation strength across tissues, prioritizing gene sets with consistent and tissue‐independent associations over those with localized or variable effects. Heatmaps were used to display the top 10 gene sets ranked by their *Z*meta ​ values, highlighting those with the strongest positive associations with GALK1 or TIMM 13 expression and illustrate tissue‐specific Spearman correlation profiles across all gene sets. Gene sets were ordered by their *Z*meta values to facilitate pattern recognition across tissues. All correlation analyses were performed using custom Python scripts with the pandas, scipy, and numpy libraries.

### 4.16 FRET‐Based Protein Interaction Assay

HEK293A cells were transfected with plasmids encoding mTurquoise2–GALK1(N‐terminus) and mCitrine–TIMM13. Cells were seeded onto dishes with a coverslip bottom coated with collagen I (Corning 354236: Steuben County, NY, USA) at low confluency 1 day prior to FRET imaging. Live‐cell imaging was performed using a Nikon Eclipse Ti2 inverted fluorescence microscope (Nikon: Tokyo, Japan) equipped with a sCMOS camera and filter sets. The following filter sets were used: excitation at 430 nm and emission at 470 nm for mTurquoise2 (donor), excitation at 485 nm and emission at 535 nm for mCitrine (acceptor), and excitation at 430 nm with emission at 535 nm for FRET signal. Background‐subtracted images were processed using the sensitized emission method. Pixel‐by‐pixel FRET quantification and visualization were conducted using the PixFRET and the FRET and Colocalization Analyzer plug‐ins for ImageJ [77, 78]. To correct for spectral bleed‐through (SBT) from the donor and acceptor fluorophores, SBT parameters were determined using control cells expressing either mTurquoise2 or mCitrine alone. We applied a linear regression to these SBT values as a function of donor and acceptor intensities to account for potential variations in crosstalk, as implemented in the PixFRET algorithm. To normalize for varying protein expression levels, the normalized FRET (nFRET) was calculated as follows:

$$\mathrm{nFRET}=\frac{I_{FRET}-\left(\alpha \times I_{D}\right)-\left(\beta \times I_{A}\right)}{\sqrt{I_{D}\times I_{A}}}\times 100$$

where donor SBT (α) and acceptor SBT (β) and *I_FRET_*, *I_D_* and *I_A_* represent the intensities in the FRET, donor and acceptor channels, respectively. The nFRET values were extracted from manually defined regions of interest (ROIs) corresponding to individual cells.

### 4.17 Assessment of Mitochondrial Membrane Potential and Mitochondrial Contents

Mitochondrial membrane potential (ΔΨm) was evaluated using the MitoProbe JC‐1 assay kit (Thermo Fisher Scientific: M34152), following the manufacturer's protocol with minor adaptations for fluorescence plate reader–based detection. Cells were seeded in black‐walled, clear‐bottom 96‐well plates at 5.0 × 10^4^ cells/well for HEK293A or at 2.0 × 10^4^ cells/well for Hep3B. After incubated overnight, cells were treated as indicated and subsequently incubated with the JC‐1 working solution (finally 2 µM) at 37 °C for 20 min in a 5% CO_2_ incubator in the dark. As a positive control for mitochondrial depolarization, cells were pre‐treated with 50 µM carbonyl cyanide 3‐chlorophenylhydrazone (CCCP) for 5 min prior to JC‐1 staining. Following staining, cells were gently washed once with warm phosphate‐buffered saline, and fluorescence was measured using Spark Mannedorf microplate reader (TECAN: Zürich, Switzerland). JC‐1 monomeric (green) fluorescence was detected at 485 nm excitation and 535 nm emission, and JC‐1 aggregate (red) fluorescence was measured at 535 nm excitation and 595 nm emission. The ratio of red‐to‐green fluorescence intensities was calculated to quantify mitochondrial membrane potential. Mitochondrial content was assessed using MitoTracker Green FM (Thermo Fisher Scientific: M7514) to stain mitochondria and Hoechst 33342 (Thermo Fisher Scientific: B2261) to stain nuclei for normalization. Fluorescence intensities were measured using a microplate reader (excitation/emission: 450/500 nm for MitoTracker, 350/460 nm for Hoechst), and mitochondrial content was calculated by normalizing mitochondrial signal to nuclear fluorescence.

### 4.18 Mitochondrial Function Test

HEK293A (1.0 × 10^5^ cells/well) or Hep3B (2.0 × 10^4^ cells/well) cells were seeded in a Corning Seahorse XF 96‐well microplate on the day of the experiment. Cells were allowed to attach for 1 h at 37°C in a CO_2_‐free incubator using Seahorse XF DMEM medium supplemented with 25 mM glucose, 1 mM sodium pyruvate, and 2 mM glutamine. Oxygen consumption rate (OCR) was measured using a Seahorse XF96e Analyzer (Agilent Technologies: Santa Clara, CA, USA) according to the standard Cell Mito Stress Test protocol. Mitochondrial respiration was sequentially challenged with 1.5 µM oligomycin (ATP synthase inhibitor), 1.0 µM FCCP (uncoupler), and 0.5 µM rotenone/antimycin A (respiratory complex inhibitors). Basal respiration was defined as the OCR before any treatment. ATP production–linked respiration was calculated as the decrease in OCR following oligomycin treatment, reflecting oxygen consumption coupled to ATP synthesis. Maximal respiration was defined as the peak OCR observed after FCCP treatment. All the defined mitochondrial OCR values were corrected by subtracting the non‐mitochondrial respiration rate, which was measured after cotreatment with rotenone and antimycin A.

### 4.19 All‐Atom Molecular‐ Dynamics‐ of TIMM13 and TIMM13‐pY73

The 3D structure of human TIMM13 were obtained from AlphaFold Protein Structure Database [79]. The structure of Y73‐phosphorylated TIMM13 (TIMM13‐pY73) was generated using AlphaFold 3 by incorporating the phosphotyrosine modification at Y73 into the human TIMM13 sequence [80]. The model with the highest average per‐residue confidence scores (pLDDT) was selected for further simulation. All‐atom molecular dynamics (MD) simulations were performed on the reduced forms of both TIMM13 and TIMM13‐pY73. The two native disulfide bonds, Cys50–Cys65 and Cys46–Cys69, were reduced to yield four thiol groups to mimic the pre‐phosphorylation, reduced state prior to modification of TIMM13 by GALK1 in the presence of ATP. Each model was solvated in a periodic box of TIP3P water containing 100 mM NaCl. Simulations were carried out in the isothermal–isobaric ensemble at 310 K and 1 bar using GROMACS with the CHARMM36 all‐atom additive protein force field [81]. Temperature was controlled with the velocity‐rescaling thermostat at 310 K, and pressure with the Parrinello–Rahman barostat at 1 bar. Long‐range electrostatics were treated with the particle‐mesh Ewald method (PME) using a 1 Å grid spacing. van der Waals (vdW) interactions were switched smoothly from 10 to 12 Å (cutoff 12 Å). Bonds involving hydrogens were constrained with LINCS, allowing a 2 fs time step. Each system was equilibrated for 100 ns, followed by a 100 ns production run used for analysis; coordinates were saved every 20 ps.

### 4.20 Modeling of the GALK1/TIMM13 Complex and Steered MD

The GALK1 structure was taken from PDB ID 1WUU [24]. The bound AMPPNP–Mg^2^^+^ was converted to MgATP by replacing AMPPNP with ATP while retaining the Mg^2^^+^ cofactor, and D‐galactose was removed. The GALK1/TIMM13 complex was generated by protein–protein docking using ZDOCK, with GALK1 (receptor) set to the prepared 1WUU model and TIMM13 (ligand) set to the reduced form obtained after a 50‐ns MD pre‐equilibration (WT‐TIMM13‐SH) [82]. Rotational sampling used an angular step size of 6°, and the interaction search was constrained toward ATP and TIMM13 Y73 within GALK1. The best‐scoring poses were subsequently minimized and equilibrated with the same MD protocol as above. For the free‐energy profile, steered MD (SMD) simulations were performed along a reaction coordinate *d* defined as the distance between the γ‐phosphorus of ATP and the phenolic oxygen of TIMM13 Y73. A harmonic pulling potential was applied with force constant *k* = 500 pN/Å and pulling speed v = 0.02 Å/ps, using a 2 fs integration time step at 310 K and 1 bar. Non‐equilibrium work values from repeated SMD trajectories were combined via Jarzynski's equality to estimate PMF as a function of *d* [83].

### 4.21 Analysis of the In‐Line Attack Geometry

To characterize the phosphotransfer geometry of the GALK1‐TIMM13 complex, a near‐contact conformer of the ZDOCK/SMD‐derived GALK1–TIMM13‐MgATP complex was subjected to distance‐conditioned MD. A harmonic umbrella restraint was applied between the phenolic oxygen of TIMM13 Y73 and the ATP γ‐phosphorus, whereas no angular restraint was imposed. The system was initially equilibrated for 0.5 ns in the NVT ensemble with a restraint centered at 0.38 nm and a force constant of 500 kJ mol^−1^ nm^−2^, followed by 2 ns of NPT equilibration with a force constant of 1000 kJ mol^−1^ nm^−2^. A 20‐ns production simulation was then performed at 310 K and 1 bar using the same distance restraint. Coordinates were saved every 10 ps. The in‐line attack angle was defined as ∠(O_Y73_–P_γ_–O_βγ_), where O_βγ_ is the ATP β‐γ bridging oxygen, and its distribution was calculated over the production trajectory.

### 4.22 Assessment of TIMM13 Affinity to Zinc Ion

HEK293T cells transiently expressing F/S‐TIMM13 WT or Y73F were lysed in a lysis buffer (50 mM Tris‐HCl pH 7.4, 150 mM NaCl, 1% NP‐40, 10% glycerol) supplemented with EDTA‐free protease inhibitor and phosphatase inhibitor cocktails. Lysates were centrifuged at 12 000 × g for 30 min at 4°C. The supernatants were subjected to sequential elution from immobilized zinc affinity chromatography [84, 85, 86]. The samples were incubated with Zn‐NTA beads for 4 h at 4°C with gentle rotation. After the beads were three times washed in an isotonic buffer (50 mM HEPES pH 7.4, 150 mM NaCl), zinc‐bound proteins were eluted sequentially by step‐wise addition of imidazole (finally 10, 30, 60, and 100 mM). The eluted fractions from each step were resolved by SDS‐PAGE, and the elution profiles of F/S‐TIMM13 WT and Y73F were analyzed by immunoblotting using an anti‐FLAG antibody.

### 4.23 Fluorescence Kinetics to Evaluate TIMM13 Oxidative Folding

F/S‐TIMM13 variants, which were expressed in HEK293T cells, were bound to streptavidin beads (Millipore; S1638) and eluted with biotin (Sigma‐Aldrich; B4501). To generate reference emission spectra for the fully oxidized and fully reduced states at 295 nm excitation wavelength, the protein samples were pre‐incubated with either 2 mM GSSG or 2 mM TCEP, respectively, for 30 min at 25°C. The acquired spectra were max‐normalized, and the precise peak emission maxima for the reduced and oxidized states were calculated using cubic B‐spline interpolation of the raw data to establish the monitoring parameters for subsequent real‐time kinetic assays. To quantify the oxidative folding kinetics of TIMM13 protein, the purified proteins were fully reduced by incubation with 2 mM TCEP for 90 min at room temperature. To remove the reducing agent, buffer exchange was immediately performed using a 3 kDa‐cutoff centrifugal filter (Millipore; UFC5003), the samples were diluted in a HEPES (50 mM, pH 7.4) buffer with 150 mM NaCl. Oxidative folding kinetics of 10 µM TIMM13 were monitored by recording tryptophan fluorescence using a fluorescence spectrophotometer (TECAN) at room temperature. For zinc inhibition assays, the samples were pre‐incubated with 40 µM ZnCl_2_ or 2 mM EDTA (zinc‐free condition) for 2 min prior to the initiation of folding. TIMM13 oxidation was initiated by the addition of 2 mM oxidized glutathione (GSSG). Fluorescence emission was monitored at 350 nm with an excitation wavelength of 295 nm [87, 88]. To confirm that the monitored signal derives from Trp77, F/S‐TIMM13‐W77A was analyzed under the same conditions. Kinetic traces were recorded for 45 min immediately following GSSG treatment. To determine kinetic parameters, time‐course fluorescence data were analyzed by non‐linear regression using GraphPad Prism software. The kinetic traces were fitted to a one‐phase association model. The zinc‐inhibited fraction of oxidative folding was quantified by calculating the reduction in the fluorescence intensity in the presence of ZnCl_2_ relative to the EDTA‐treated control [30].

### 4.24 Immunofluorescence

Cells were cultured on coverslips and incubated with MitoTracker Red CMXRos (Invitrogen: M7512) for 30 min to label mitochondria. Subsequently, cells were fixed with 4% paraformaldehyde, permeabilized with 0.1% Triton X‐100, and blocked with a 3% **BSA** in PBS containing 0.1% Tween‐20. The cells were sequentially incubated with an anti‐HIS(6) or anti‐FLAG antibody and an Alexa Fluor 488‐conjugated secondary antibody (Invitrogen: A‐11001) in PBS containing 0.1% Tween‐20. To compare the distributions of total and Y73‐phosphorylated TIMM13, cells were co‐incubated with the anti‐TIMM13 and anti‐TIMM13‐pY73 antibodies, followed by Alexa Fluor 647‐conjugated chicken anti‐rabbit IgG (Thermo Fisher Scientific: A‐21443) and Alexa Fluor 488‐conjugated goat anti‐human IgG (Invitrogen: A‐11013). After nuclear staining with DAPI (Sigma‐Aldrich: D9542), the coverslips were mounted using FluorSave Reagent (Millipore: 345789). Fluorescence images were acquired using a Zeiss **LSM** 800 confocal microscope. Colocalization images and calculation of the Manders’ Colocalization Coefficients for the analyzed images were performed using a combination of the Coloc **2** plugins in ImageJ 3.10v (National Institutes of Health, USA) [89]. M1, defined as the fraction of the antibody signal overlapping MitoTracker, was calculated separately for total TIMM13 and for TIMM13‐pY73.

### 4.25 Fractionation of Soluble and Insoluble Proteins

HEK293T cells were lysed in the **TNE** buffer containing 50 mM Tris–HCl (pH7.4), 150 mM NaCl, 1 mM EDTA, 1% (v/v) Triton X‐100, and a protease inhibitor cocktail. After the lysates were centrifuged at 15 000 **× **g for 10 min at 4°C, the supernatant and pellet were collected as the soluble and insoluble fractions. The pellets were twice washed with the TNE buffer, resuspended with 2% SDS, and sonicated briefly. After centrifugation at 15 000 **× **g for 10 min at room temperature, the supernatants were used as insoluble fractions [90].

### 4.26 Filtration‐Based Extraction of Aggregated Protein Fractions

To specifically detect high‐molecular‐weight aggregates, HEK293T cells were lysed in the TNE buffer containing 50 mM Tris–HCl (pH7.4), 150 mM NaCl, 1 mM EDTA, 1% (v/v) Triton X‐100, and a protease inhibitor cocktail and subjected to ultrafiltration. The lysate was loaded onto a 200 kDa molecular weight cutoff filter (ADVANTEC USY‐20: Tokyo, Japan) and filtered according to the manufacturer's protocol. The flow‐through, containing proteins and complexes smaller than 200 kDa, was collected. The high‐molecular‐weight aggregates retained on the filter were then recovered by solubilizing them in SDS buffer.

### 4.27 Statistics

All statistical analyses were performed using Microsoft Excel 2016 (Microsoft Corp., Redmond, WA, USA) or GraphPad Prism 7.0 (GraphPad Software, La Jolla, CA, USA). Data are presented as the means ± standard deviation (SD) with the number of biological replicates indicated in the figure legends. Statistical comparisons between two groups were performed using an unpaired, two‐tailed Student's *t*‐test. *p* value of less than 0.05 was considered statistically significant.

## Author Contributions

J.W.P. and C.W.K. conceived and designed the research. C.W.K. performed the experiments, assessments and analyzed the data. C.W.K. performed bioinformatic analysis. J.W.P. and C.W.K. constructed expression vectors. Y.K., J.P., D.S. performed and analyzed LC‐MS. J.H. performed and analyzed MD. C.W.K. and J.W.P. wrote the manuscript. All authors read and approved the final version.

## Consent

No written consent has been required because this study did not include any data related with patients.

## Conflicts of Interest

The authors declare no competing interests.

## Supporting information




**Supporting File 1**: advs77996‐sup‐0001‐SuppMat.pdf.

**Supporting File 2**: advs77996‐sup‐0002‐S1.xlsx.

**Supporting File 3**: advs77996‐sup‐0003‐S2.xlsx.

**Supporting File 4**: advs77996‐sup‐0004‐S3.xlsx.

**Supporting File 5**: advs77996‐sup‐0005‐S4.docx.

**Supporting File 6**: advs77996‐sup‐0006‐S5.xlsx.

**Supporting File 7**: advs77996‐sup‐0007‐S6.docx.

**Supporting File 8**: advs77996‐sup‐0008‐S7.xlsx.

## Data Availability

The raw mass spectrometry data have been deposited and are available in the ProteomeXchange Consortium via the PRIDE partner repository with the dataset identifier PXD075859. Any additional information and original code required to reanalyze the data reported in this paper is available from the lead contact upon request.
