# Supplementary material for: Galactokinase 1 Positively Regulates Mitochondrial Respiration by Phosphorylating TIMM13 as a Protein Kinase
Source: Adv Sci (Weinh). 2026 Sep 30:e77996. Online ahead of print. doi: 10.1002/advs.77996 (PMC13625921; doi:10.1002/advs.77996)

Galactokinase 1 positively regulates mitochondrial respiration by phosphorylating TIMM13 as a protein kinase

Chang Woo Ko[1], Dongyoon Shin[1,2,*], Junho Park[2,3,*], June Huh[4], Youngsoo Kim[1,2], Jong-Wan Park[1,5,6*]

[1] Department of Biomedical Sciences, College of Medicine, Seoul National University, Seoul, Republic of Korea

[2] Department of Medical Science, School of Medicine, CHA University, Seongnam, Republic of Korea

[3] Department of Medical Science, Research Institute for Basic Medical Science, School of Medicine, CHA University, Seongnam, Republic of Korea

[4] Department of Chemical and Biological Engineering, Korea University, Seoul, Republic of Korea

[5] Department of Pharmacology, College of Medicine, Seoul National University, Seoul, Republic of Korea

[6] Ischemic/Hypoxic Disease Institute, Seoul National Medical Research Center, Seoul National University, Seoul, Republic of Korea

Correspondence: parkjw@snu.ac.kr, jpark@cha.ac.kr, sdymath777@chamc.co.kr

Lead Contact: parkjw@snu.ac.kr

List of Supplementary Materials

Figure S1. GALK1 does not substantially phosphorylate PROSER2. Related to Figure 2.

Figure S2. GALK1 phosphorylates TIMM13 at tyrosine residues. Related to Figure 3.

Figure S3. GALK1 interacts with TIMM13 *in situ* in HEK293A cells. Related to Figure 2.

Figure S4. LC-MS spectra for Y73 phosphorylation of TIMM13 extracted from an *in vitro* kinase assay. Related to Figure 4.

Figure S5. Verification of GALK1-mediated phosphorylation of Y73 in TIMM13. Related to Figures 3 and 4.

Figure S6. Structural overview of the GALK1 active site. Related to Figure 4.

Figure S7. Functional correlation analysis of TIMM13 expression in cancer and normal tissues. Related to Figure 5.

Figure S8. Mitochondrial integrity is maintained depending on the GALK1-TIMM13 axis. Related to Figures 5 and 6.

Figure S9. Y73 phosphorylation of TIMM13 is required for Zn^{2+}-induced prevention of its oxidative folding. Related to Figure 6.

Figure S10. GALK1 is required for mitochondrial translocation of TIMM13 and for its interaction with TIMM8A. Related to Figure 6.

Figure S11. Intracellular distribution and catalytic activity of galactosemia-associated GALK1 variants. Related to Figure 7.

Figure S12. GALK1 is required for mitochondrial integrity. Related to Figure 7.

Figure S13. Original Western blot images corresponding to the main figures.

Figure S14. Original Western blot images corresponding to the supplementary figures.

Table S1. Meta-analysis of correlations between GALK1 expression and ssGSEA pathway enrichment scores. Related to Figure 1.

Table S2. Proteomic identification of potential GALK1-interacting proteins by LC-MS analyses. Related to Figure 2.

Table S3. Identified phosphorylated and unmodified peptides in TIMM13 from in vitro kinase assay experiments. Related to Figure 4.

Table S4. Information on matched fragment ions corresponding to the LC-MS/MS spectra of Figure S4. Related to Figure 4.

Table S5. Identified phosphorylated and unmodified peptides in TIMM13 from HEK293T cells. Related to Figure 4.

Table S6. Information of matched fragment ions related to LC-MS/MS spectra of Figure 4B. Related to Figure 4.

Table S7. Meta-analysis of correlations between TIMM13 expression and ssGSEA pathway enrichment scores. Related to Figure 5.

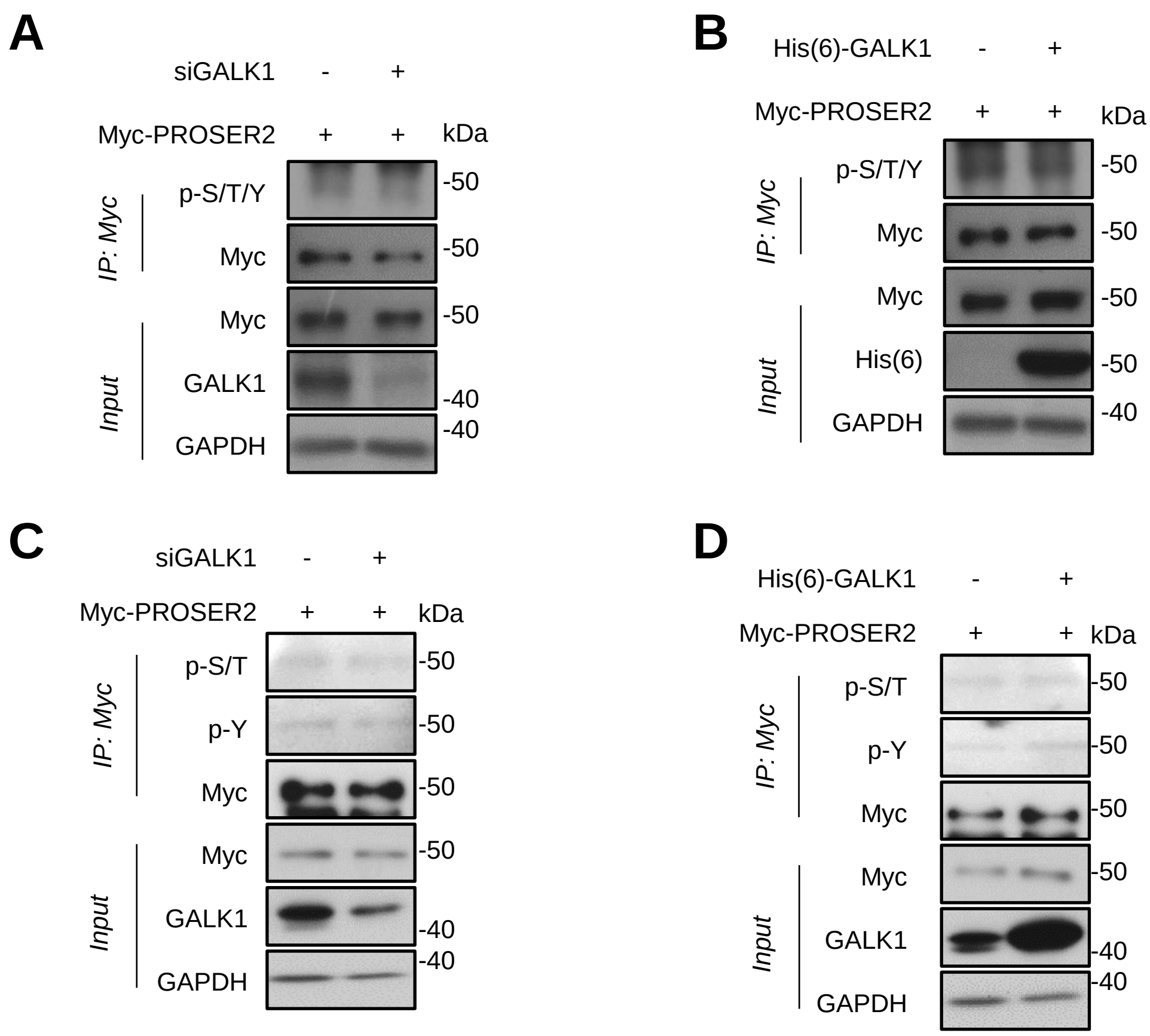

Figure S1. GALK1 does not substantially phosphorylate PROSER2. Related to Figure 2.

(A-B) Phosphorylation of immunoprecipitated myc-PROSER2, detected with a pan-phospho antibody, was unaffected by either GALK1 knockdown (**A**) or His(6)-GALK1 overexpression (**B**) in HEK293T cells.

(C-D) Phosphorylation of immunoprecipitated myc-PROSER2, detected with phospho-Ser/Thr- and phospho-Tyr-specific antibodies, was unaffected by either GALK1 knockdown (**C**) or His(6)-GALK1 overexpression (**D**) in HEK293T cells.

Original blot can be found in Figure S14.

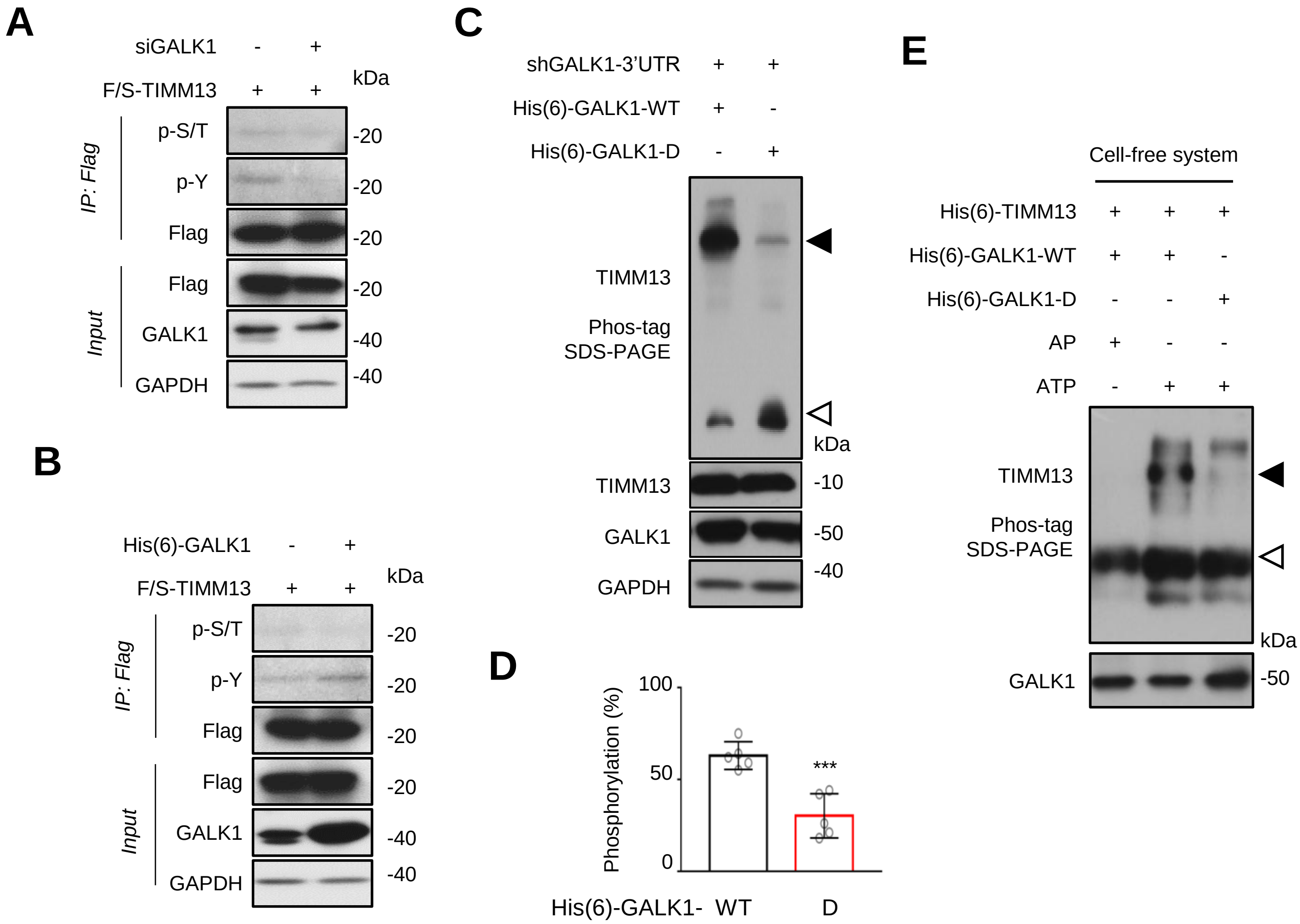

Figure S2. GALK1 phosphorylates TIMM13 at tyrosine residues. Related to Figure 3. (**A-B**) F/S-TIMM13 was immunoprecipitated, electrophoresed, and immunoblotted with phospho-Ser/Thr-specific and phospho-Tyr-specific antibodies. Immunoblot signal was obtained only with the phospho-Tyr antibody, which was reduced by GALK1 knockdown (**A**) but enhanced by GALK1 overexpression (**B**). (**C-D**) HEK293T cells were co-transfected with an shRNA targeting the 3'UTR of *GALK1* mRNA and a plasmid for shRNA-resistant His(6)-GALK1-WT or the kinase-dead mutant His(6)-GALK1-D. The cell lysates were subjected to Mn^{2+}-Phos-tag SDS-PAGE and immunoblotting with anti-TIMM13 antibody. Closed and open arrowheads indicate the phosphorylated and non-phosphorylated species, respectively (**C**). The phosphorylation ratio was calculated as the intensity of the upshifted band relative to the sum of both bands (**D**). **(E)** An *in vitro* kinase assay was performed with recombinant His(6)-TIMM13 and His(6)-GALK1-WT or the kinase-dead mutant His(6)-GALK1-D in the presence or absence of ATP and alkaline phosphatase (AP), as indicated. The reaction mixtures were subjected to Phos-tag SDS-PAGE and immunoblotting with anti-TIMM13 antibody. Closed and open arrowheads indicate the phosphorylated and non-phosphorylated species, respectively. Data are presented as the means ± SD (n = 5). Statistical significance was determined using unpaired two-tailed Student-*t* test. *** denotes $P < 0.001$. Original blot can be found in Figure S14.

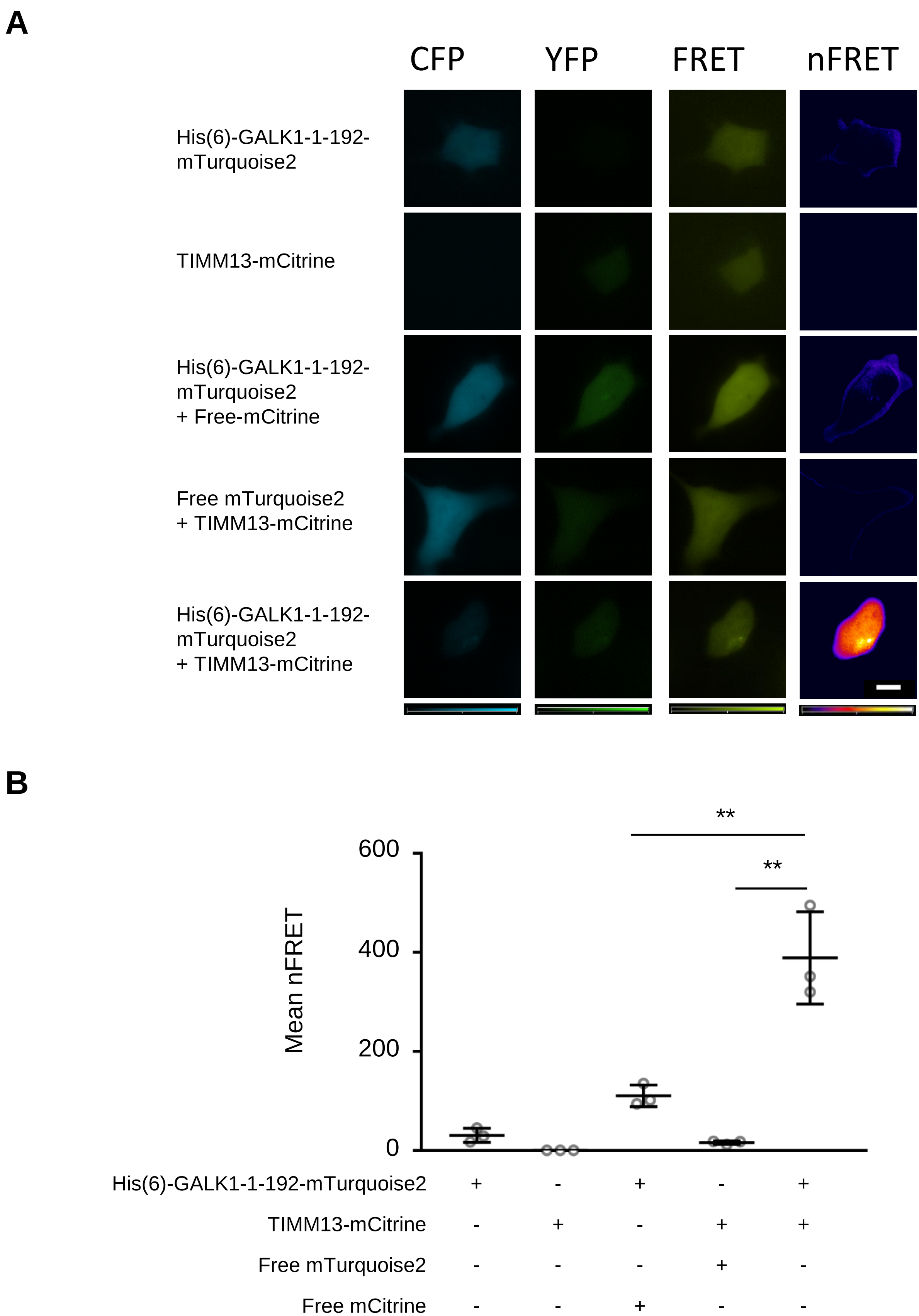

A
CFP
YFP
FRET
nFRET
His(6)-GALK1-1-192-mTurquoise2
TIMM13-mCitrine
His(6)-GALK1-1-192-mTurquoise2 + Free-mCitrine
Free mTurquoise2 + TIMM13-mCitrine
His(6)-GALK1-1-192-mTurquoise2 + TIMM13-mCitrine
B
Mean nFRET
0
200
400
600
**
**
His(6)-GALK1-1-192-mTurquoise2 + - + - +
TIMM13-mCitrine - + - + +
Free mTurquoise2 - - - + -
Free mCitrine - - + - -

Figure S3. GALK1 interacts with TIMM13 *in situ* in HEK293A cells. Related to Figure 2.

(A) Representative nFRET images of HEK293A cells expressing the indicated FRET pairs. Cells were transfected with His(6)-GALK1-1-192-mTurquoise2 (donor) and TIMM13-mCitrine (acceptor), or corresponding negative controls employing free mTurquoise2 or free mCitrine. Images were acquired in CFP (donor), YFP (acceptor), and FRET channels. The calculated normalized FRET (nFRET) signal is displayed in pseudocolor, with the intensity scale bar shown at the bottom. Scale bar, 10 μm.

(B) Quantitative analysis of mean nFRET signals. A significant increase in nFRET efficiency was observed between His(6)-GALK1-1-192-mTurquoise2 and TIMM13-mCitrine compared to negative controls co-expressed with free fluorescent proteins. Data are presented as mean ± SD (n = 3 biological replicates). Statistical significance for comparisons was determined using unpaired two-tailed t test. ** denotes $P < 0.01$ versus the His(6)-GALK1-1-192-mTurquoise2 + TIMM13-mCitrine group.

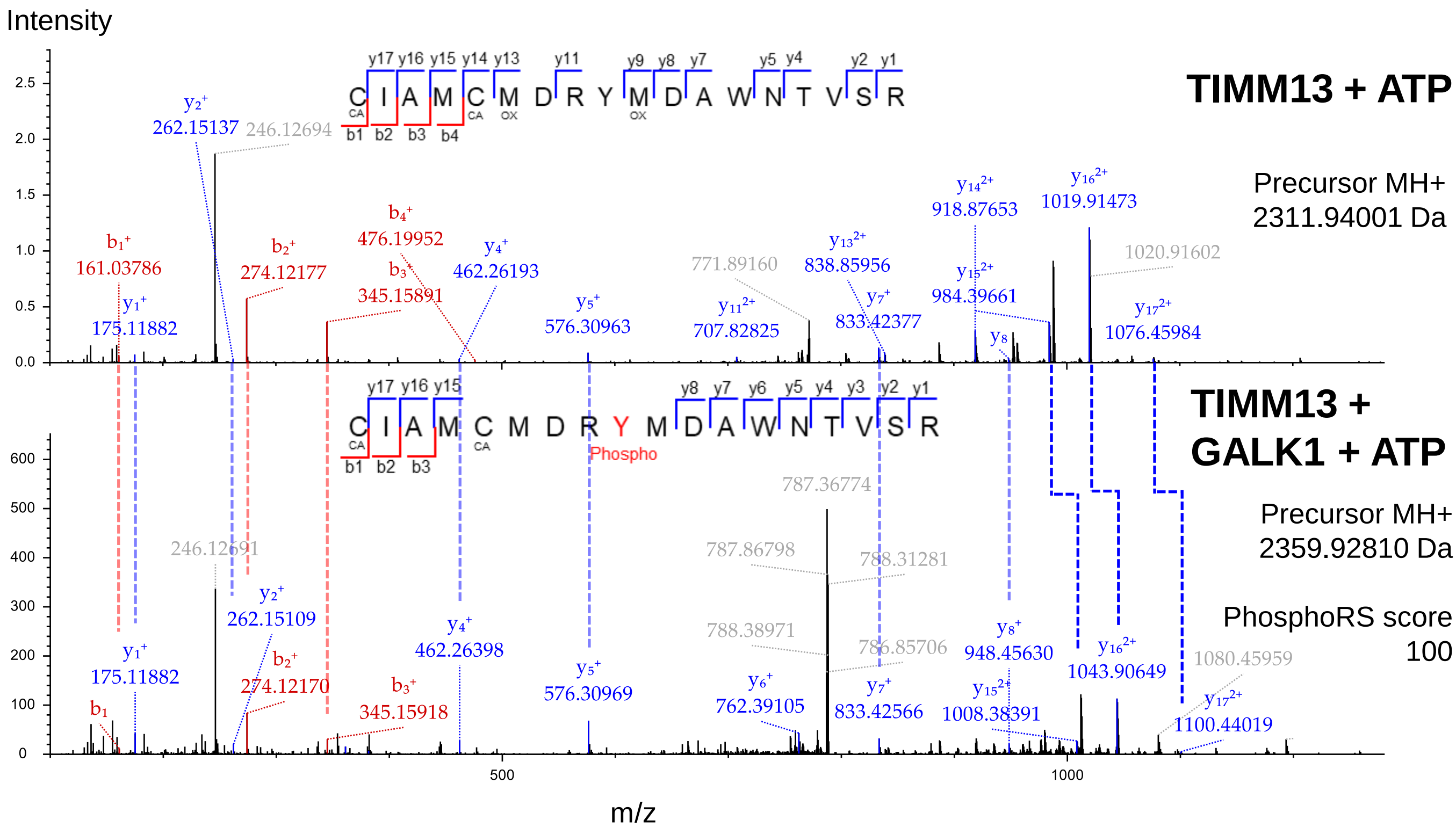

Figure S4. LC-MS spectra for Y73 phosphorylation of TIMM13 extracted from an *in vitro* kinase assay. Related to Figure 4.

In vitro phosphorylation analysis was performed by mixing 6xHis-GALK1, purified from HEK293T cells using a mammalian expression system, and bacterially purified 6xHis–TIMM13 in the presence of ATP. Representative LC-MS/MS spectra of the unmodified peptide (upper panel) and the Y73-phosphorylated peptide (lower panel). All fragment ion matches along the peptide sequence and the corresponding precursor ion information are also shown. The PhosphoRS site-localization score for the phosphorylated peptide is also provided in the bottom panel.

Abbreviations: LC-MS, liquid chromatography–mass spectrometry; CA, carbamidomethylation; OX, oxidation; Phospho, phosphorylation.

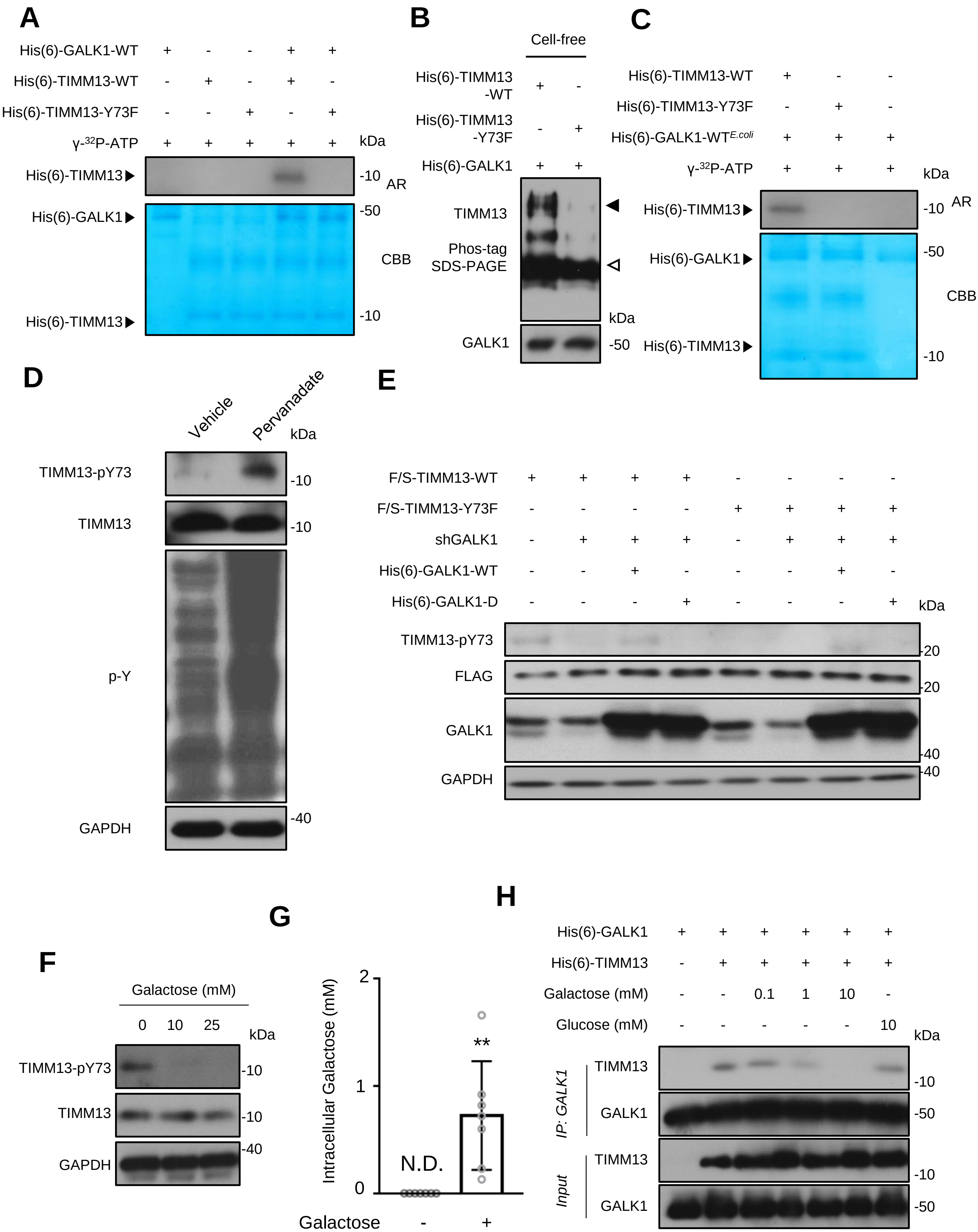
A
His(6)-GALK1-WT + - - + +
His(6)-TIMM13-WT - + - + -
His(6)-TIMM13-Y73F - - + - +
γ-32P-ATP + + + + +
kDa
His(6)-TIMM13
-10
AR
His(6)-GALK1
-50
CBB
His(6)-TIMM13
-10
B
Cell-free
His(6)-TIMM13 -WT + -
His(6)-TIMM13 -Y73F - +
His(6)-GALK1 + +
TIMM13
Phos-tag SDS-PAGE
kDa
GALK1
-50
C
His(6)-TIMM13-WT + - -
His(6)-TIMM13-Y73F - + -
His(6)-GALK1-WTE.coli + + +
γ-32P-ATP + + +
kDa
His(6)-TIMM13
-10
AR
His(6)-GALK1
-50
CBB
His(6)-TIMM13
-10
D
Vehicle
Pervanadate
kDa
TIMM13-pY73
-10
TIMM13
-10
p-Y
GAPDH
-40
E
F/S-TIMM13-WT + + + + - - - -
F/S-TIMM13-Y73F - - - - + + + +
shGALK1 - + + + - + + +
His(6)-GALK1-WT - - + - - - + -
His(6)-GALK1-D - - - + - - - +
kDa
TIMM13-pY73
-20
FLAG
-20
GALK1
-40
GAPDH
-40
F
Galactose (mM)
0 10 25
kDa
TIMM13-pY73
-10
TIMM13
-10
GAPDH
-40
G
Intracellular Galactose (mM)
2
1
0
N.D.
**
Galactose - +
H
His(6)-GALK1 + + + + + +
His(6)-TIMM13 - + + + + +
Galactose (mM) - - 0.1 1 10 -
Glucose (mM) - - - - - 10
kDa
IP: GALK1
TIMM13
-10
GALK1
-50
Input
TIMM13
-10
GALK1
-50

Figure S5. Verification of GALK1-mediated phosphorylation of Y73 in TIMM13. Related to Figures 3 and 4.

(A) Y73 is the sole residue to be phosphorylated by GALK1. The *in vitro* kinase assay was performed with recombinant His(6)-TIMM13-WT or His(6)-TIMM13-Y73F as the substrate for GALK1. **(B)** An *in vitro* kinase assay was performed with recombinant His(6)-TIMM13-WT or His(6)-TIMM13-Y73F as substrate for recombinant His(6)-GALK1 in the presence of ATP. The reaction mixtures were subjected to Phos-tag SDS-PAGE and immunoblotting with anti-TIMM13 antibody. Closed and open arrowheads indicate the phosphorylated and non-phosphorylated species, respectively. **(C)** Recombinant GALK1 alone can phosphorylate TIMM13 *in vitro*. An *in vitro* kinase assay was performed with commercially available human GALK1 expressed in *E. coli* and recombinant His(6)-TIMM13-WT or His(6)-TIMM13-Y73F as substrate, and ^{32}P incorporation was visualized by autoradiography (AR). Coomassie brilliant blue (CBB) staining confirms equal protein loading. **(D)** Verification of tyrosine phosphorylation of TIMM13. HEK293T cells were treated with 100 μM pervanadate or vehicle for 15 min before harvest, and lysates were immunoblotted for Y73-phosphorylated TIMM13, total TIMM13, and global tyrosine phosphorylation (p-Y). **(E)** Verification of GALK1-dependent tyrosine phosphorylation of TIMM13. HEK293T cells expressing F/S-TIMM13-WT or F/S-TIMM13-Y73F were transfected with shGALK1 alone or together with shRNA-resistant His(6)-GALK1-WT or the kinase-dead mutant His(6)-GALK1-D. Y73 phosphorylation was detected with the anti-TIMM13-pY73 antibody, and comparable expression of both TIMM13 proteins was confirmed by anti-FLAG immunoblotting. **(F)** Galactose competes with TIMM13 in the GALK1's kinase reaction. Immunoblotting analysis for TIMM13 phosphorylation in HEK293T cells incubated with or without galactose. Cells were treated with increasing concentrations of galactose (0, 10, and 25 mM) as indicated for 24 hr. **(G)** Intracellular galactose levels. After HEK293T cells were incubated with or without 10 mM galactose for 24 h, the intracellular galactose was quantified in deproteinized lysates and normalized to the packed cell volume. N.D., not detected. Data are presented as the means ± SD (n = 7). Statistical significance was determined using unpaired two-tailed *t* test. ** denotes $P < 0.01$. **(H)** Galactose competes with TIMM13 for binding to GALK1 in a cell-free system. Purified His(6)-GALK1 was preincubated with galactose (0.1, 1, or 10 mM) or glucose (10 mM) before the addition of purified His(6)-TIMM13, and the complex was recovered with an anti-GALK1 antibody. Original blot can be found in Figure S14.

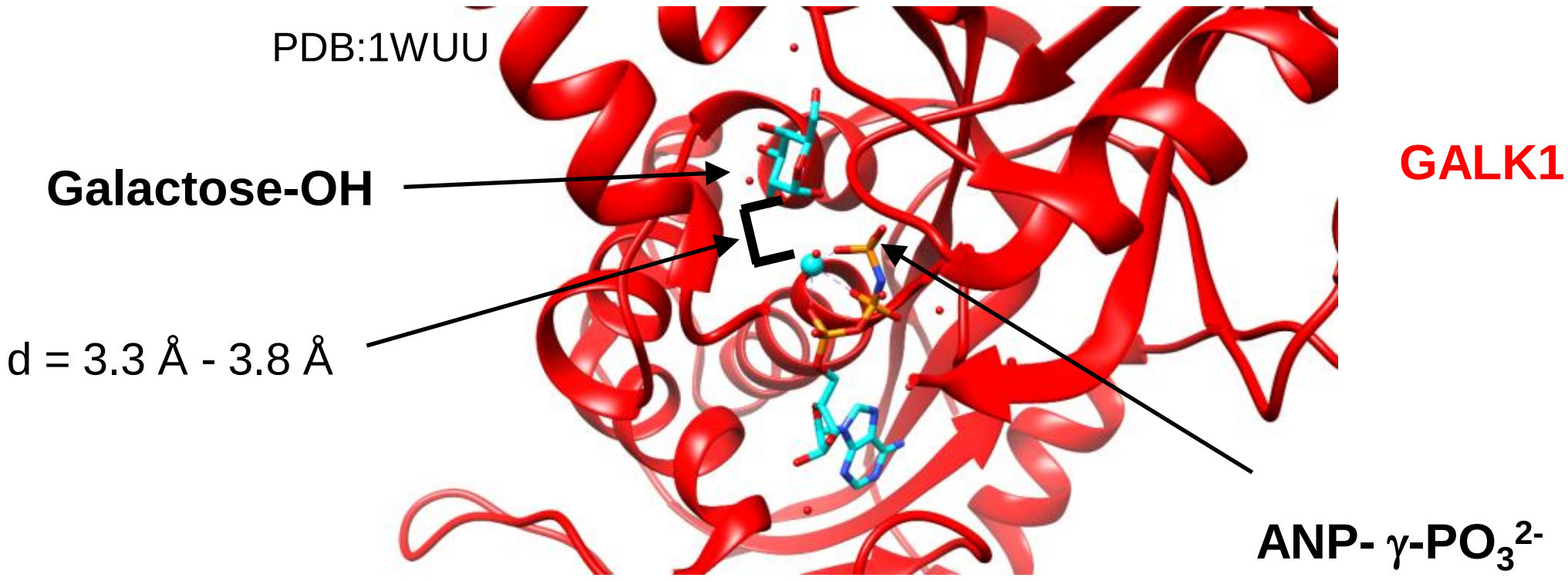

Figure S6. Structural overview of the GALK1 active site. Related to Figure 4.

The ribbon diagram (red) represents the structure of human GALK1, derived from PDB entry 1WUU. The structure was resolved in complex with its substrate, alpha-D-galactose analog alpha-D-galactopyranose (PDB ID: GLA), and the non-hydrolyzable ATP analog AMPPNP (PDB ID: ANP). Both ligands are displayed as stick models (cyan) to indicate their positions within the enzyme's binding pocket. Mg is shown as a ball (cyan).

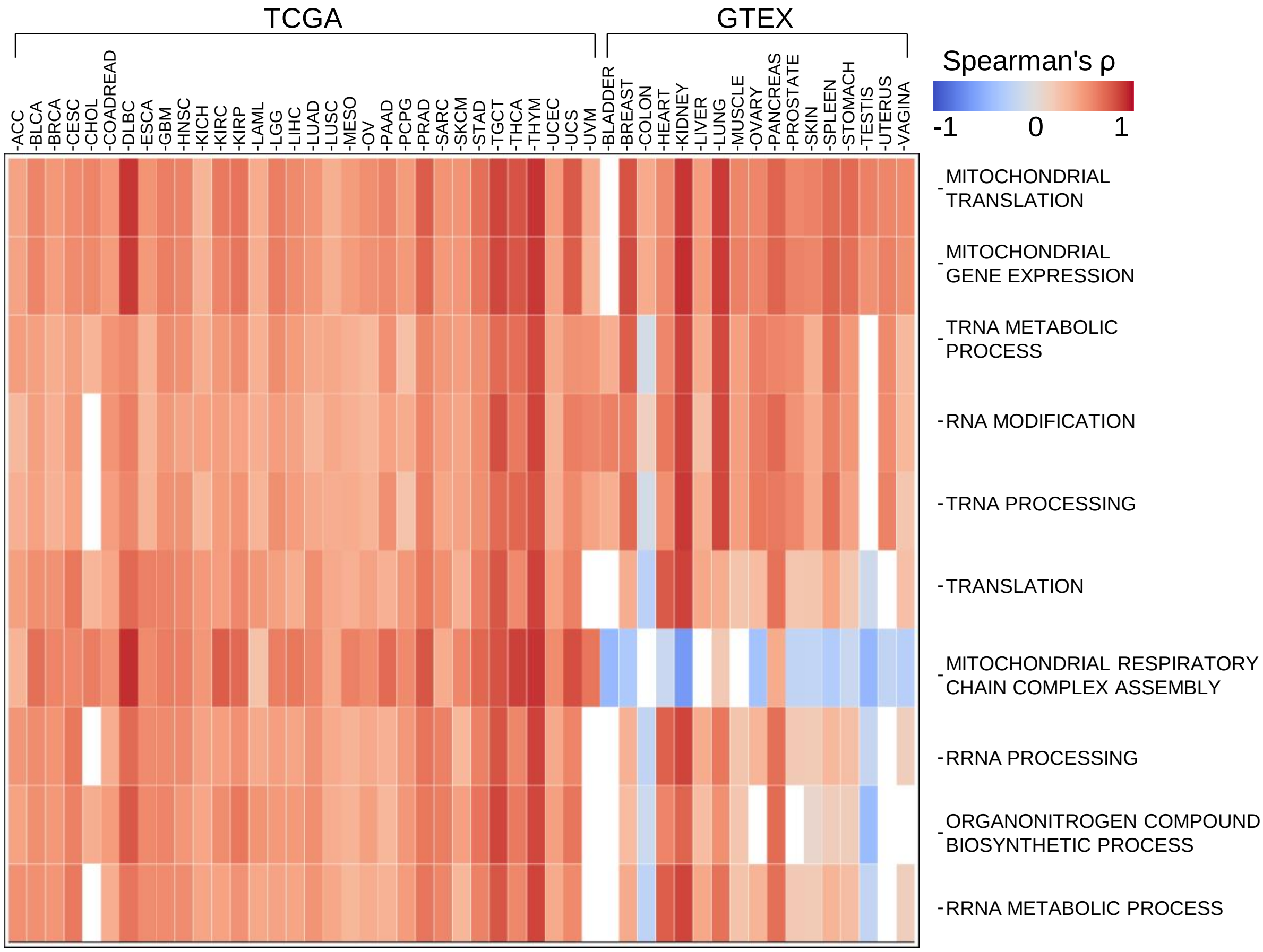

Figure S7. Functional correlation analysis of TIMM13 expression in cancer and normal tissues. Related to Figure 5.

Correlation heatmap of TIMM13 expression with ssGSEA score of Gene Ontology (GO) biological processes across TCGA and GTEx datasets. The color scale represents the Spearman's rank correlation coefficient (ρ), ranging from red (positive correlation, +1) to blue (negative correlation, -1). Non-significant correlations ($p > 0.05$) are masked in white.

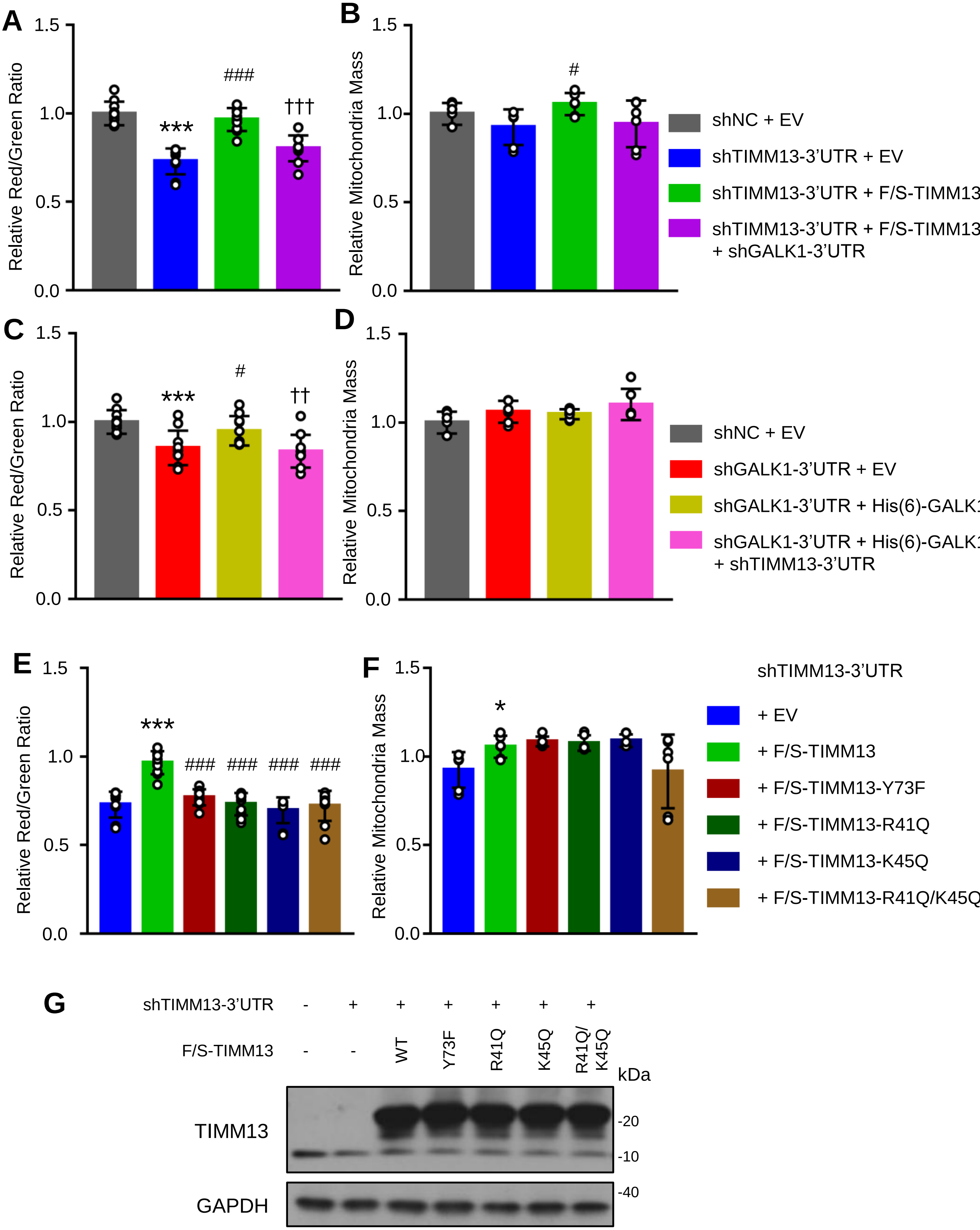

A
B
C
D
E
F
G
Relative Red/Green Ratio
Relative Mitochondria Mass
1.5
1.0
0.5
0.0
***
###
†††
#
††
*
shNC + EV
shTIMM13-3'UTR + EV
shTIMM13-3'UTR + F/S-TIMM13
shTIMM13-3'UTR + F/S-TIMM13 + shGALK1-3'UTR
shGALK1-3'UTR + EV
shGALK1-3'UTR + His(6)-GALK1
shGALK1-3'UTR + His(6)-GALK1 + shTIMM13-3'UTR
shTIMM13-3'UTR
+ EV
+ F/S-TIMM13
+ F/S-TIMM13-Y73F
+ F/S-TIMM13-R41Q
+ F/S-TIMM13-K45Q
+ F/S-TIMM13-R41Q/K45Q
F/S-TIMM13
WT
Y73F
R41Q
K45Q
R41Q/ K45Q
kDa
TIMM13
GAPDH
-20
-10
-40

Figure S8. Mitochondrial integrity is maintained depending on the GALK1-TIMM13 axis. Related to Figures 5 and 6.

(A-B) Quantification of (**A**; n = 10 per group) mitochondrial membrane potential, measured by JC-1 assay (Relative Red/Green Ratio), and relative mitochondrial mass, measured by MitoTracker Green staining (**B**; n = 6 per group), in HEK293A cells transfected with control (shNC + EV), shTIMM13-3'UTR + EV, shTIMM13-3'UTR + F/S-TIMM13 (rescue), or shTIMM13-3'UTR + F/S-TIMM13 + shGALK1-3'UTR. Asterisks(*) indicate comparison to the shNC + EV control group, hash symbols(#) indicate comparison to the shTIMM13-3'UTR + EV knockdown group and daggers(†) indicate comparison to the shTIMM13-3'UTR + F/S-TIMM13 rescue group.

(C-D) Quantification of (**C**; n = 10 per group) mitochondrial membrane potential, measured by JC-1 assay (Relative Red/Green Ratio), and relative mitochondrial mass, measured by MitoTracker Green staining (**D**; n = 6 per group), in HEK293A cells transfected with control (shNC + EV), shGALK1-3'UTR + EV, shGALK1-3'UTR + His(6)-GALK1 (rescue), or shGALK1-3'UTR + His(6)-GALK1 + shTIMM13-3'UTR. Asterisks(*) indicate comparison to the shNC + EV control group, hash symbols(#) indicate comparison to the shGALK1-3'UTR + EV knockdown group and daggers(†) indicate comparison to the shGALK1-3'UTR + His(6)-GALK1 rescue group.

(E-F) Quantification of (**E**; n = 10 per group) mitochondrial membrane potential, measured by JC-1 assay (Relative Red/Green Ratio), and relative mitochondrial mass, measured by MitoTracker Green staining (**F**; n = 6 per group), in HEK293A cells transfected with shTIMM13-3’UTR and co-expressing empty vector (EV), shRNA-resistant F/S-TIMM13, or the indicated shRNA-resistant F/S-TIMM13 point mutants (Y73F, R41Q, K45Q, R41Q/K45Q). Asterisks(*) indicate comparison to the shTIMM13-3'UTR + EV knockdown group, hash symbols(#) indicate comparison to the shTIMM13-3'UTR + F/S-TIMM13 rescue group.

Statistical significance for comparisons was determined using unpaired two-tailed t test. All data are presented as mean ± SD. $^{*}P$, $^{\#}P$, $^{\dagger}P < 0.05$, $^{**}P$, $^{\#\#}P$, $^{\dagger\dagger}P < 0.01$, $^{***}P$, $^{\#\#\#}P$, $^{\dagger\dagger\dagger}P < 0.001$.

(G) Immunoblot analysis of HEK293A cells co-transfected with an shRNA targeting the 3′ untranslated region (3′UTR) of endogenous TIMM13 and shRNA-resistant, F/S-tagged TIMM13 constructs (WT or indicated mutants). The lower band corresponds to endogenous TIMM13, while the upper bands represent the ectopically expressed TIMM13 variants resistant to knockdown. GAPDH serves as a loading control. Original blot can be found in Figure S14.

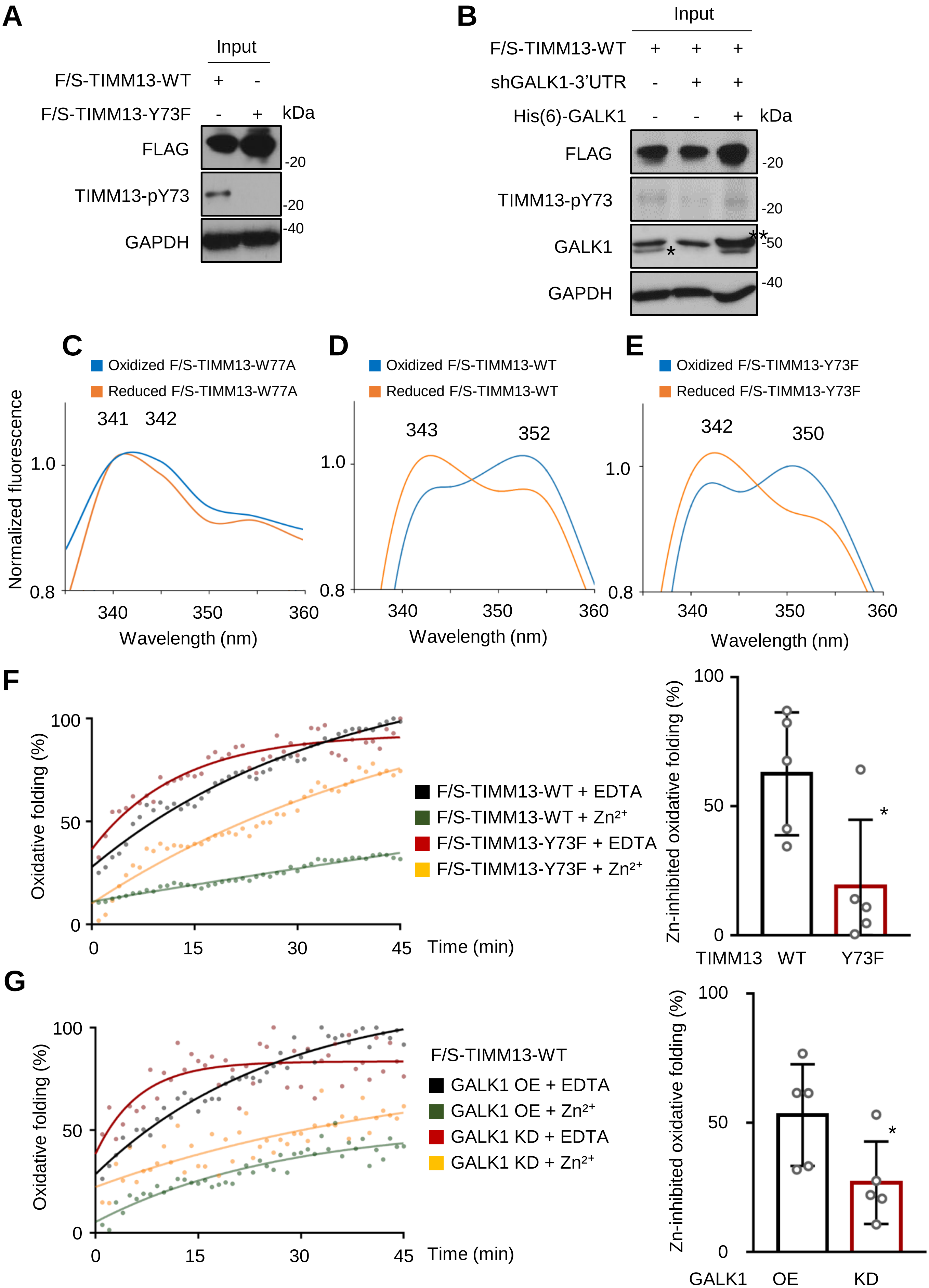

A
Input
F/S-TIMM13-WT + -
F/S-TIMM13-Y73F - + kDa
FLAG -20
TIMM13-pY73 -20
GAPDH -40
B
Input
F/S-TIMM13-WT + + +
shGALK1-3'UTR - + +
His(6)-GALK1 - - + kDa
FLAG -20
TIMM13-pY73 -20
GALK1 * ** -50
GAPDH -40
C
Oxidized F/S-TIMM13-W77A
Reduced F/S-TIMM13-W77A
341 342
Normalized fluorescence
Wavelength (nm)
D
Oxidized F/S-TIMM13-WT
Reduced F/S-TIMM13-WT
343 352
Wavelength (nm)
E
Oxidized F/S-TIMM13-Y73F
Reduced F/S-TIMM13-Y73F
342 350
Wavelength (nm)
F
Oxidative folding (%)
Time (min)
F/S-TIMM13-WT + EDTA
F/S-TIMM13-WT + Zn²⁺
F/S-TIMM13-Y73F + EDTA
F/S-TIMM13-Y73F + Zn²⁺
Zn-inhibited oxidative folding (%)
TIMM13 WT Y73F
*
G
Oxidative folding (%)
Time (min)
F/S-TIMM13-WT
GALK1 OE + EDTA
GALK1 OE + Zn²⁺
GALK1 KD + EDTA
GALK1 KD + Zn²⁺
Zn-inhibited oxidative folding (%)
GALK1 OE KD
*

Figure S9. Y73 phosphorylation of TIMM13 is required for Zn^{2+}-induced prevention of its oxidative folding. Related to Figure 6.

(A-B) Immunoblot analysis of total cell lysates used as inputs for the SIMAC assays in Figure 6G (**A**) and Figure 6H (**B**). * and ** indicate the bands of GALK1 and His(6)-GALK1, respectively. Original blot can be found in Figure S14.

(C-E) Intrinsic tryptophan fluorescence emission spectra of W77A-mutated TIMM13 (C), wild-type TIMM13 (D) and Y73F-mutated TIMM13 (E) proteins, all of which were excited at 295 nm. Proteins were fully oxidized with 2 mM GSSG (blue) or fully reduced with 2 mM TCEP (orange). Spectra were max-normalized, with peak maxima determined via cubic B-spline interpolation of the raw data.

(F-G) Time course for oxidative folding of TIMM13. Fluorescence emission was monitored at 350 nm with an excitation wavelength of 295 nm. Oxidative folding of reduced WT or Y73F TIMM13 (**F**), and WT TIMM13 derived from GALK1-overexpressing (OE) or GALK1-knockdown (KD) cells (**G**), was monitored in the presence of EDTA or 40 μM $ZnCl_2$. Scatter points represent the mean and lines indicate one-phase association model fitting. Bar graphs quantify the Zn^{2+}-inhibited fraction of TIMM13 oxidative folding at 45 min (n = 5). Statistical significance for comparisons was calculated by Student's t test. All data are presented as the means ± SD. * denotes $P < 0.05$.

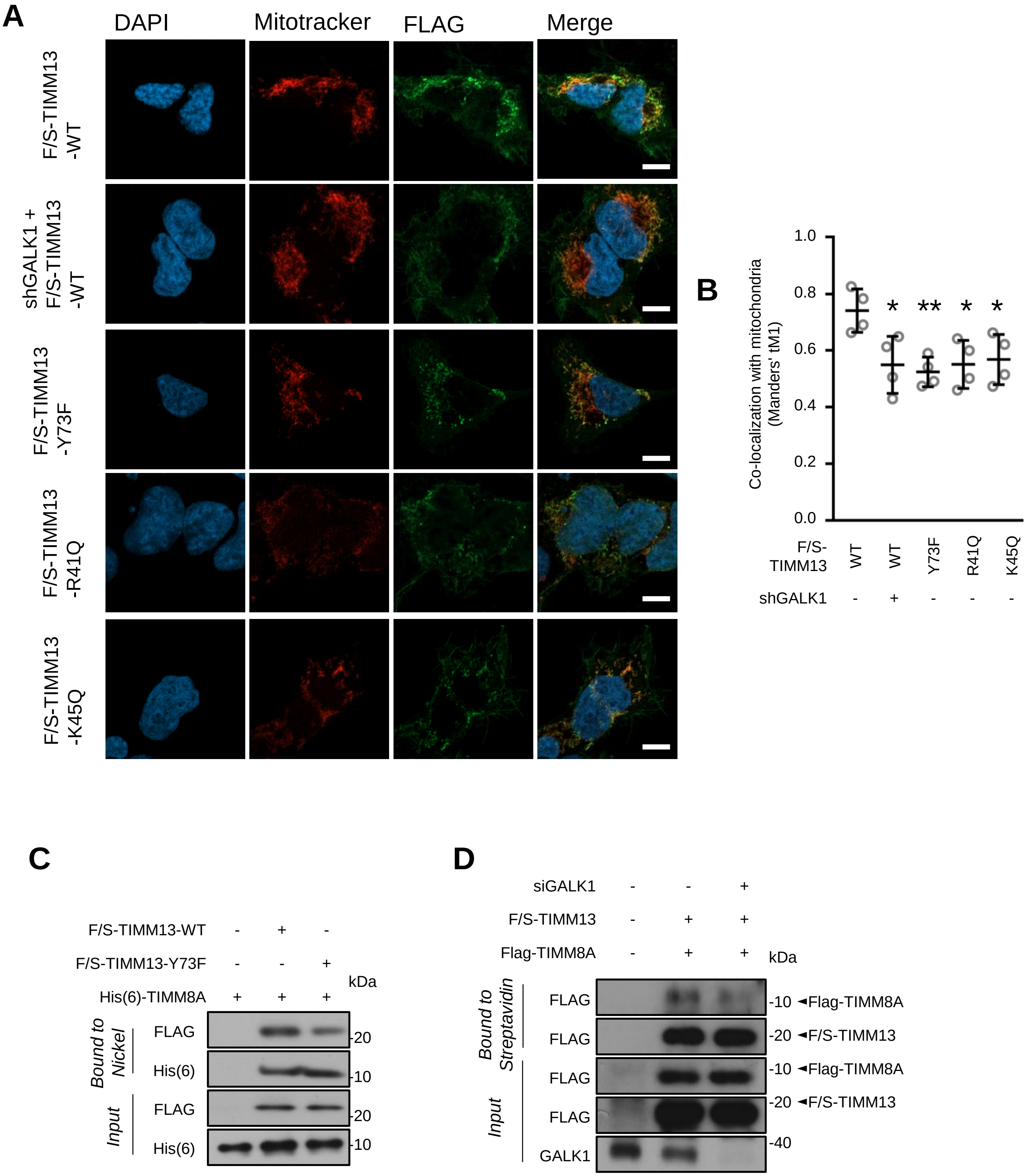

A
DAPI
Mitotracker
FLAG
Merge
F/S-TIMM13 -WT
shGALK1 + F/S-TIMM13 -WT
F/S-TIMM13 -Y73F
F/S-TIMM13 -R41Q
F/S-TIMM13 -K45Q
B
Co-localization with mitochondria (Manders' tM1)
1.0
0.8
0.6
0.4
0.2
0.0
*
**
*
*
F/S-TIMM13
WT
WT
Y73F
R41Q
K45Q
shGALK1
-
+
-
-
-
C
F/S-TIMM13-WT
F/S-TIMM13-Y73F
His(6)-TIMM8A
kDa
Bound to Nickel
Input
FLAG
His(6)
20
10
D
siGALK1
F/S-TIMM13
Flag-TIMM8A
kDa
Bound to Streptavidin
Input
FLAG
GALK1
-10 ◄Flag-TIMM8A
-20 ◄F/S-TIMM13
-40

Figure S10. GALK1 is required for mitochondrial translocation of TIMM13 and for its interaction with TIMM8A. Related to Figure 6.

(A) Representative confocal immunofluorescence images of HEK293A cells expressing Flag-tagged wild-type (WT) or mutant TIMM13 constructs. Cells were transfected with the indicated F/S-TIMM13 variants in the presence or absence of shRNA targeting GALK1 (shGALK1). TIMM13 was detected using an anti-Flag antibody (green), mitochondria were labeled with MitoTracker (red), and nuclei were stained with DAPI (blue). Scale bars, 10 μm.

(B) Quantitative assessment of TIMM13 mitochondrial localization using Manders' colocalization coefficient (tM1). tM1 represents the fraction of the FLAG-TIMM13 signal colocalizing with the mitochondrial marker above Costes' automatic threshold. Data are presented as mean ± SD (n = 4 biological replicates). Statistical significance for comparisons was calculated by Student's t test. * denotes $P < 0.05$, ** denotes $P < 0.01$ versus the F/S-TIMM13-WT group.

(C, D) GALK1-mediated Y73 phosphorylation of TIMM13 is required for the TIMM13 association with TIMM8A. (**C**) His(6)-TIMM8A was co-expressed with F/S-TIMM13-WT or F/S-TIMM13-Y73F in HEK293T cells and subjected to nickel-affinity chromatography of His(6)-TIMM8A, followed by immunoblotting with anti-FLAG antibody. (**D**) HEK293T cells expressing F/S-TIMM13 and Flag-TIMM8A were transfected with GALK1-targeting siRNA. F/S-TIMM13 was pulled down with streptavidin-affinity beads. GALK1 depletion was confirmed in the input. Original blot can be found in Figure S14.

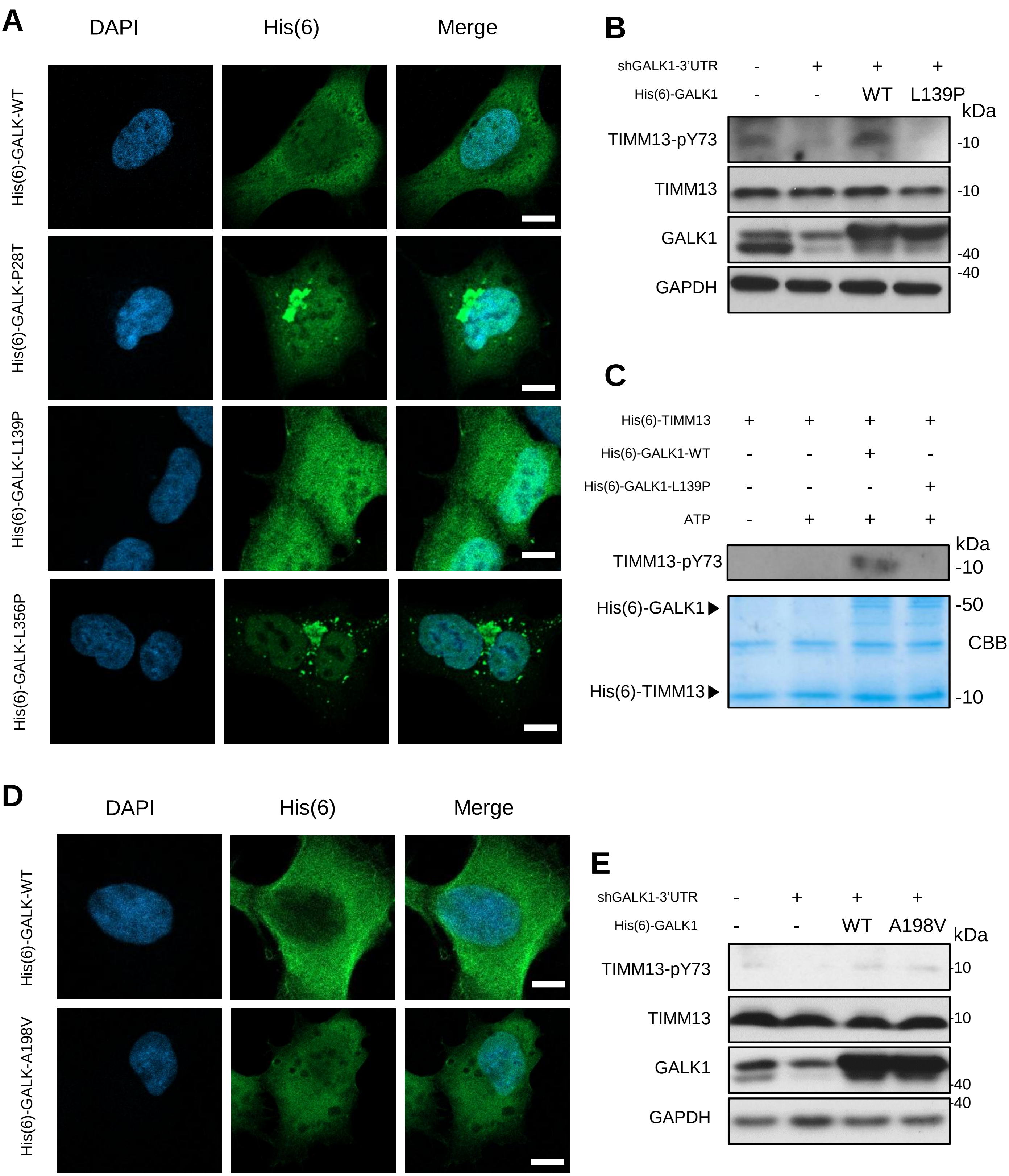
A
DAPI
His(6)
Merge
His(6)-GALK-WT
His(6)-GALK-P28T
His(6)-GALK-L139P
His(6)-GALK-L356P
B
shGALK1-3'UTR - + + +
His(6)-GALK1 - - WT L139P
kDa
TIMM13-pY73
TIMM13
GALK1
GAPDH
-10
-10
-40
-40
C
His(6)-TIMM13 + + + +
His(6)-GALK1-WT - - + -
His(6)-GALK1-L139P - - - +
ATP - + + +
TIMM13-pY73
kDa
-10
His(6)-GALK1
His(6)-TIMM13
-50
CBB
-10
D
DAPI
His(6)
Merge
His(6)-GALK-WT
His(6)-GALK-A198V
E
shGALK1-3'UTR - + + +
His(6)-GALK1 - - WT A198V
kDa
TIMM13-pY73
TIMM13
GALK1
GAPDH
-10
-10
-40
-40

Figure S11. Intracellular distribution and catalytic activity of galactosemia-associated GALK1 variants. Related to Figure 7.

(A) Confocal microscopy images of HEK293A cells transiently expressing His(6)-tagged wild-type (WT) GALK or the pathogenic variants P28T, L139P and L356P. GALK proteins were visualized using an anti-His(6) antibody (green), and nuclei were counterstained with DAPI (blue). Scale bars, 10 μm.

(B) Immunoblot analysis of TIMM13 phosphorylation in HEK293T cells reconstituted with wild-type (WT) or mutant GALK1. Endogenous GALK1 was depleted using an shRNA targeting the 3'UTR, followed by the expression of shRNA-resistant His(6)-GALK1-WT or the Type 2 galactosemia-associated pathogenic mutant, His(6)-GALK1-L139P. Phosphorylation of endogenous TIMM13 at Y73 was assessed using a specific anti-TIMM13-pY73 antibody. GAPDH served as a loading control.

(C) In vitro kinase assay examining the direct phosphorylation of TIMM13 by GALK1 variants. Recombinant His(6)-TIMM13 was incubated with purified His(6)-GALK1-WT or His(6)-GALK1-L139P in the presence of ATP. Phosphorylation signals were detected by immunoblotting with the anti-TIMM13-pY73 antibody. Total protein loading for GALK1 and TIMM13 was visualized by Coomassie Brilliant Blue (CBB) staining.

(D) Confocal microscopy images of HEK293A cells expressing His(6)-GALK1-WT or the high-frequency variant His(6)-GALK1-A198V, imaged as in (A). Scale bars, 10 μm.

(E) Immunoblot analysis of TIMM13 phosphorylation in HEK293T cells reconstituted with His(6)-GALK1-WT or His(6)-GALK1-A198V after depletion of endogenous GALK1 with an shRNA targeting the 3'UTR. Phosphorylation of endogenous TIMM13 at Y73 was assessed using the anti-TIMM13-pY73 antibody, and GAPDH served as a loading control.

Original blot can be found in Figure S14.

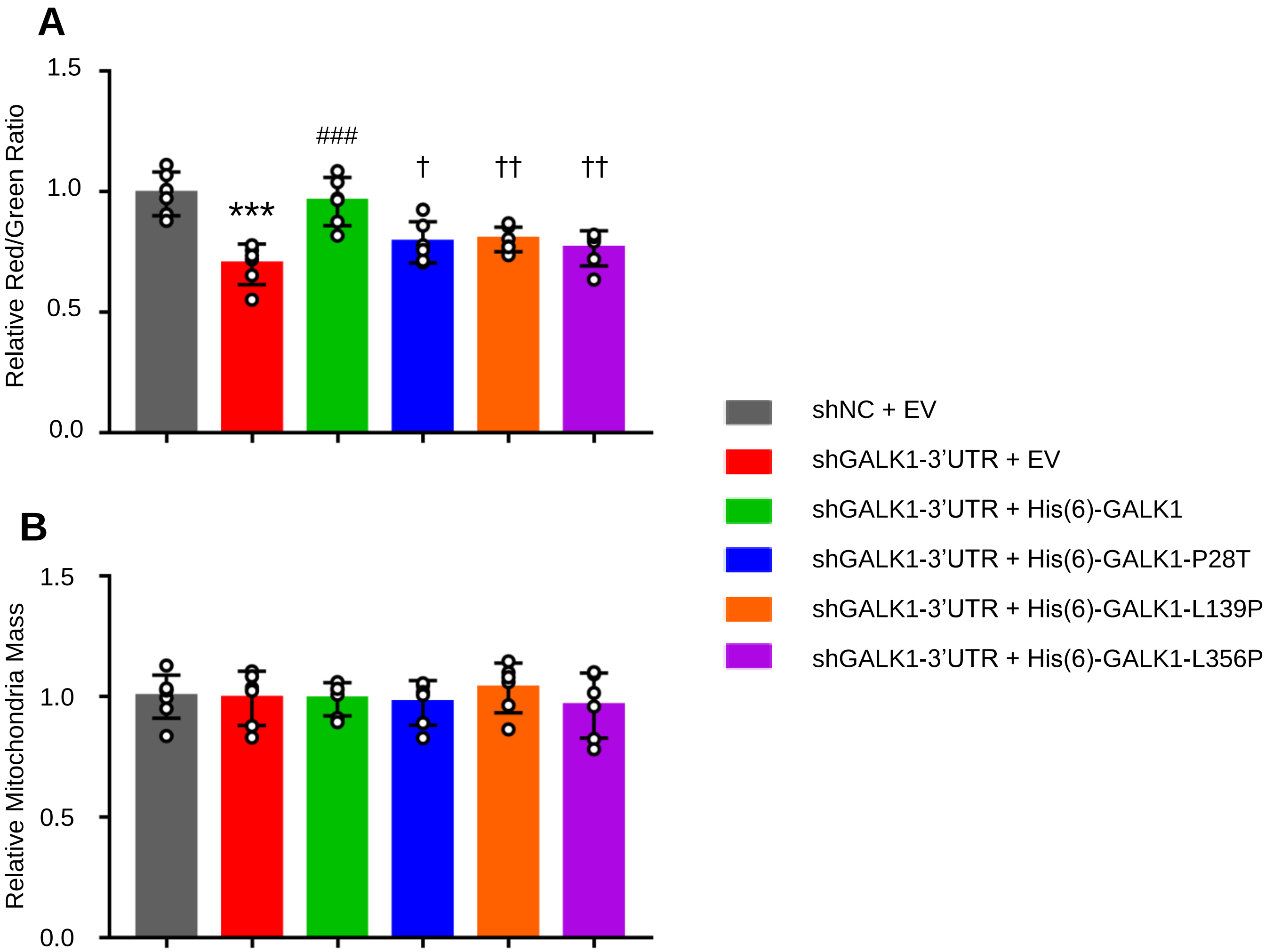

Figure S12. GALK1 is required for mitochondrial integrity. Related to Figure 7.

(A-B) Mitochondrial membrane potential was measured using JC-1 assay (Relative Red/Green Ratio) (**A**). Relative mitochondrial mass was measured by MitoTracker Green staining (**B**). Hep3B cells were transfected with shNC + EV, shGALK1-3'UTR + EV, shGALK1-3'UTR + His(6)-GALK1 (rescue), shGALK1-3'UTR + His(6)-GALK1-P28T, shGALK1-3'UTR + His(6)-GALK1-L139P or shGALK1-3'UTR + His(6)-GALK1-L356P. Asterisks(*) indicate the comparison to the shNC + EV group, hash symbols(#) to the shGALK1-3'UTR + EV knockdown group, and daggers(†) to the shGALK1-3'UTR + His(6)-GALK1 rescue group. Statistical significance for comparisons was calculated by Student’s t test. All data are presented as the means ± SD (n = 6). †, $P < 0.05$; ††, $P < 0.01$; *** and $^{\#\#\#}$, $P < 0.001$.

Figure S13. Original Western blot images corresponding to the main figures.

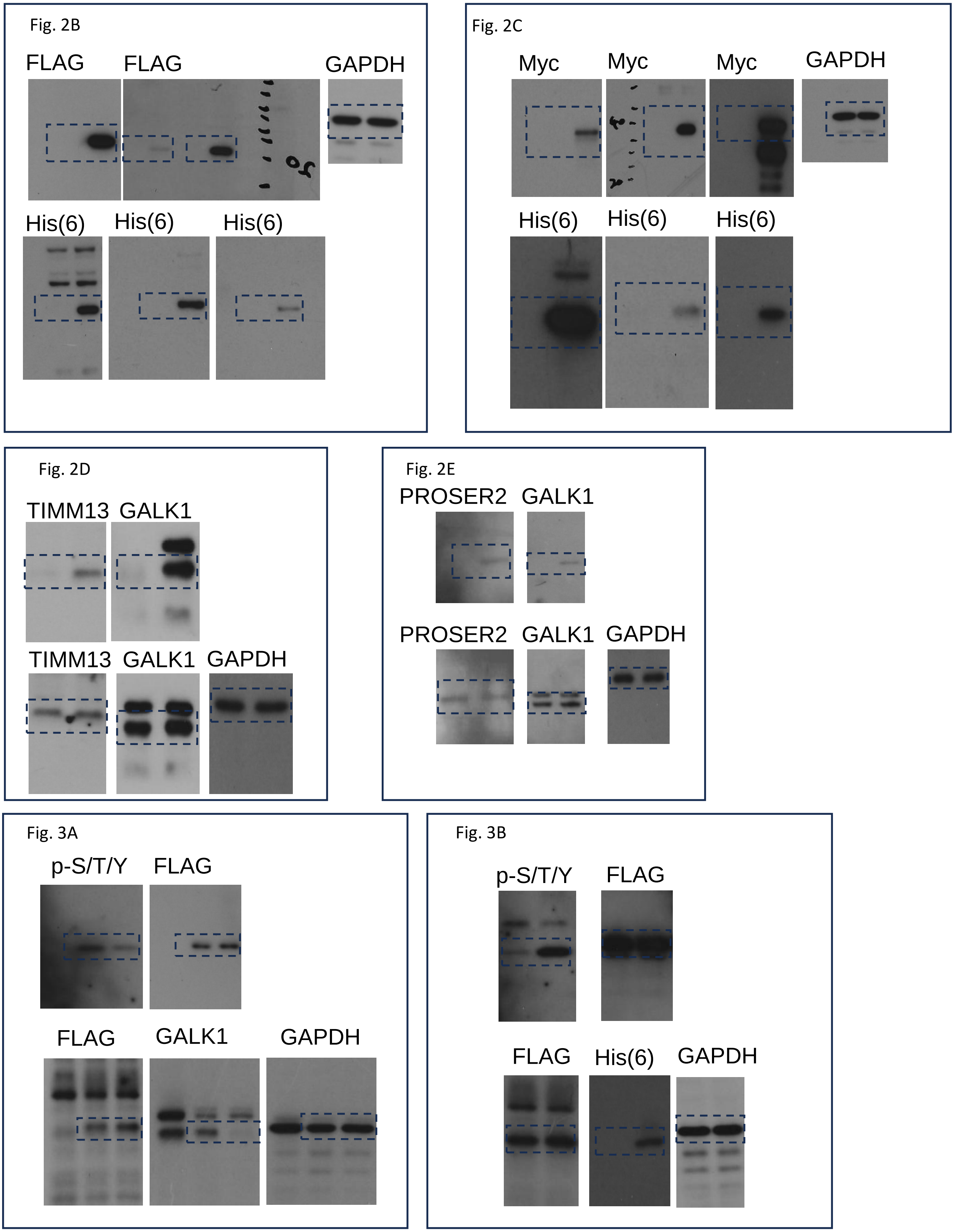

Figure S13. Original Western blot images corresponding to the main figures.

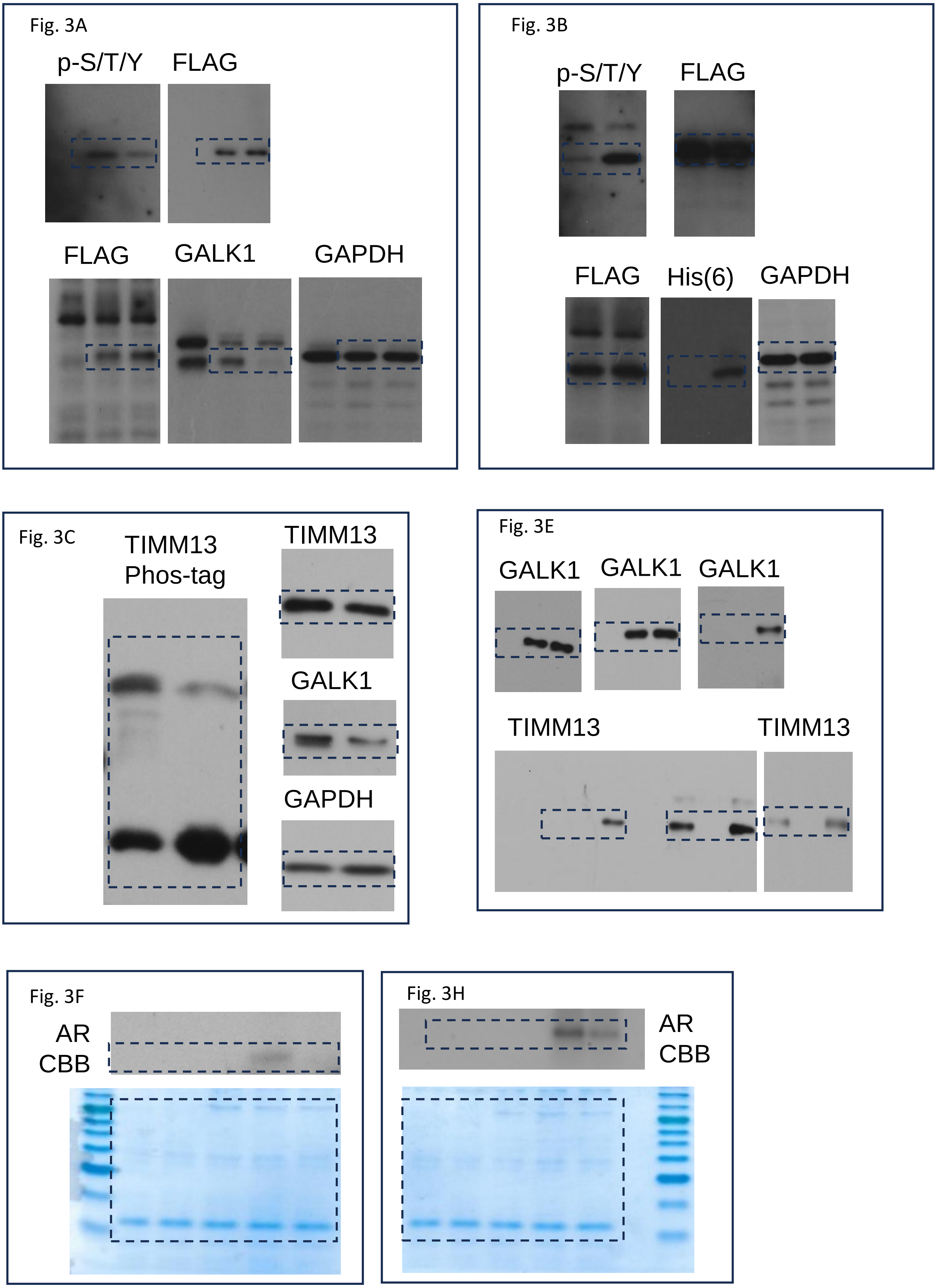

Figure S13. Original Western blot images corresponding to the main figures.

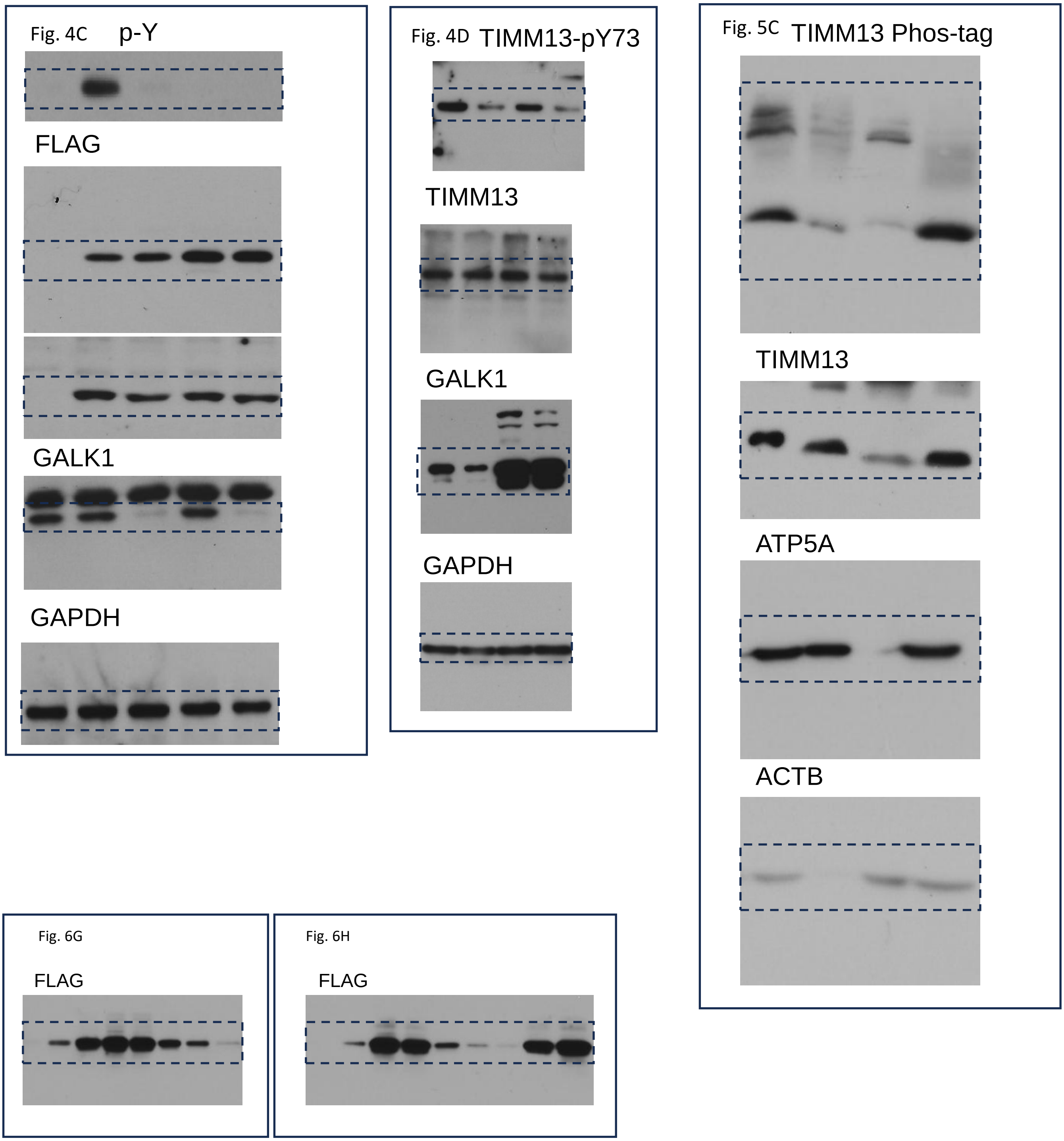

Figure S13. Original Western blot images corresponding to the main figures.

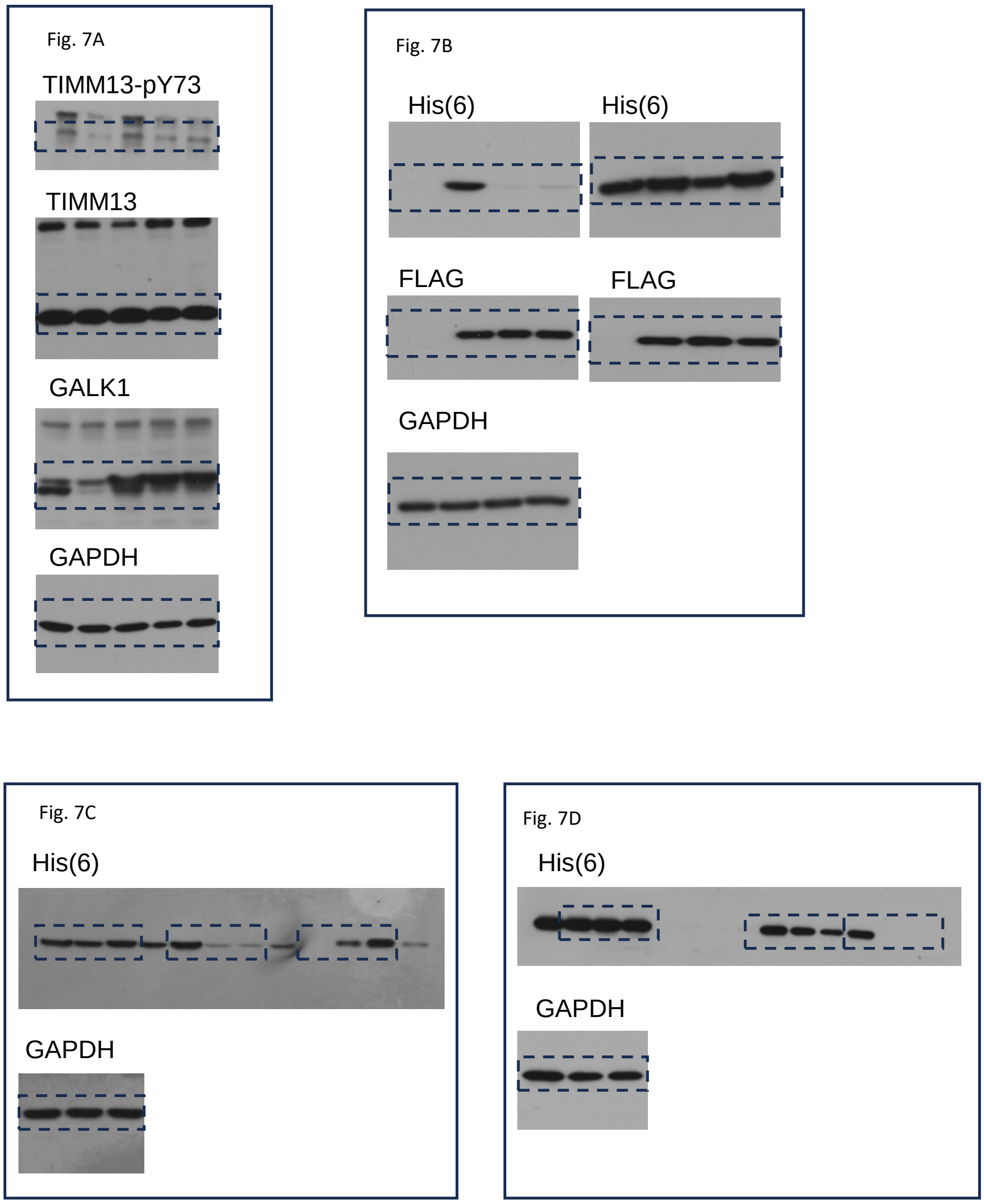

Figure S14. Original Western blot images corresponding to the supplementary figures.

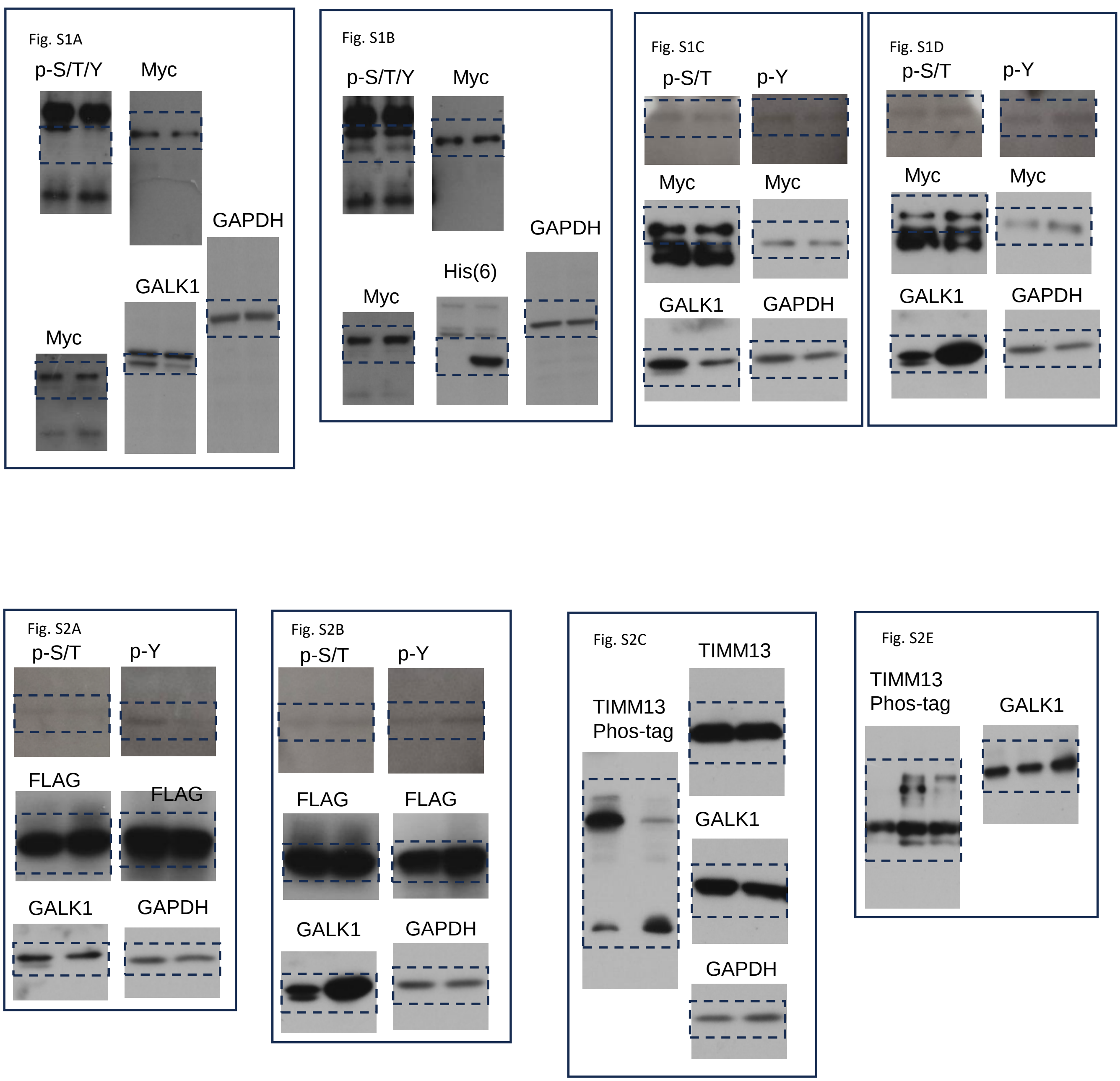

Figure S14. Original Western blot images corresponding to the supplementary figures.

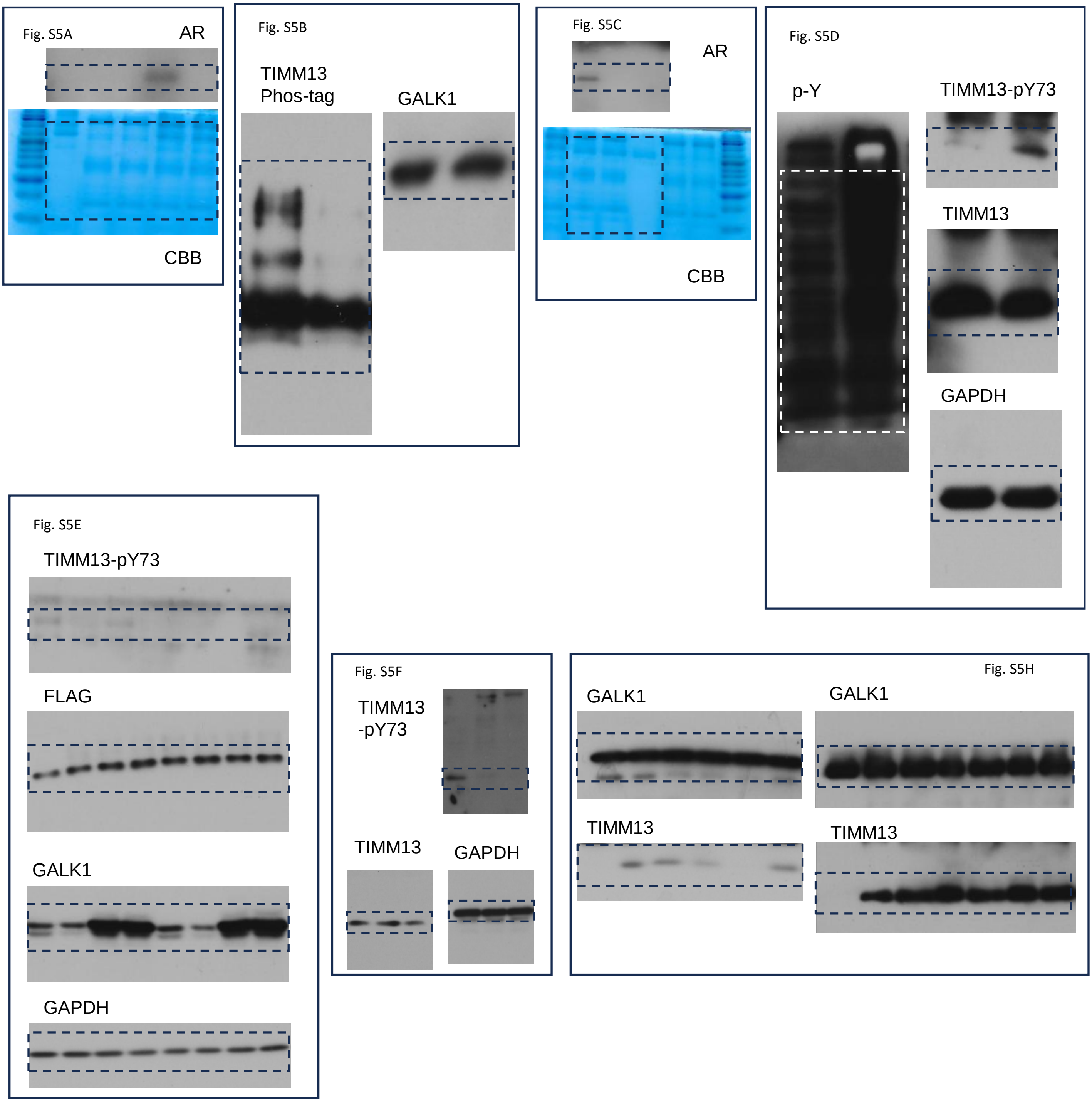

Figure S14. Original Western blot images corresponding to the supplementary figures.

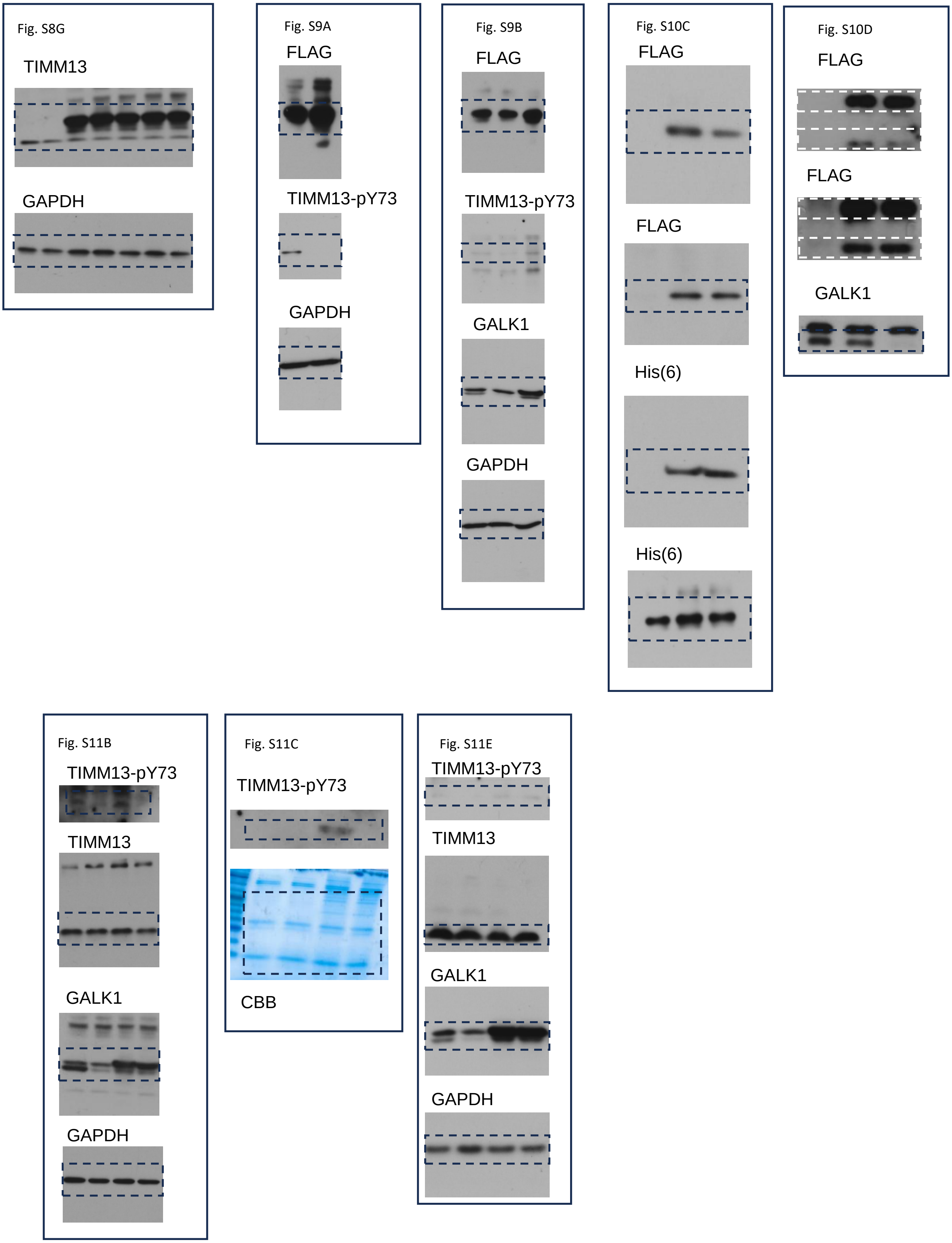

Supplement: Supplementary file 1 [file ADVS-9999-e77996-s005.pdf]
