# Supplementary material for: Galactokinase 1 Positively Regulates Mitochondrial Respiration by Phosphorylating TIMM13 as a Protein Kinase
Source: Adv Sci (Weinh). 2026 Sep 30:e77996. Online ahead of print. doi: 10.1002/advs.77996 (PMC13625921; doi:10.1002/advs.77996)
Supplement: Supplementary file 2 [file ADVS-9999-e77996-s001.docx]

**Table S6. Information of matched fragment ions related to LC-MS/MS spectra of Figure 4a. a** Fragment ions detected in LC-MS/MS spectra of CIAMCMDRYMDAWNTVSR in un-phosphorylated. **b** Fragment ions detected in MS/MS spectra of CIAMCMDRYMDAWNTVSR in phosphorylated. Matched b ions colored in red and y ions in blue.

**a**

| #1 | b^+^ | b^2+^ | b^3+^ | Seq | y^+^ | y^2+^ | y^3+^ | #2 |
| --- | --- | --- | --- | --- | --- | --- | --- | --- |
| 1 | 161.03793 | 81.02260 | 54.35083 | C-Carbamidomethyl |  |  |  | 18 |
| 2 | 274.12199 | 137.56463 | 92.04551 | I | 2135.91295 | 1068.46011 | 712.64250 | 17 |
| 3 | 345.15910 | 173.08319 | 115.72455 | A | 2022.82888 | 1011.91808 | 674.94781 | 16 |
| 4 | 476.19959 | 238.60343 | 159.40471 | M | 1951.79177 | 976.39952 | 651.26877 | 15 |
| 5 | 636.23024 | 318.61876 | 212.74826 | C-Carbamidomethyl | 1820.75128 | 910.87928 | 607.58861 | 14 |
| 6 | 767.27072 | 384.13900 | 256.42842 | M | 1660.72064 | 830.86396 | 554.24506 | 13 |
| 7 | 882.29766 | 441.65247 | 294.77074 | D | 1529.68015 | 765.34371 | 510.56490 | 12 |
| 8 | 1038.39877 | 519.70303 | 346.80444 | R | 1414.65321 | 707.83024 | 472.22259 | 11 |
| 9 | 1201.46210 | 601.23469 | 401.15889 | Y | 1258.55210 | 629.77969 | 420.18888 | 10 |
| 10 | 1348.49750 | 674.75239 | 450.17069 | M-Oxidation | 1095.48877 | 548.24802 | 365.83444 | 9 |
| 11 | 1463.52445 | 732.26586 | 488.51300 | D | 948.45337 | 474.73032 | 316.82264 | 8 |
| 12 | 1534.56156 | 767.78442 | 512.19204 | A | 833.42643 | 417.21685 | 278.48033 | 7 |
| 13 | 1720.64087 | 860.82407 | 574.21848 | W | 762.38931 | 381.69829 | 254.80129 | 6 |
| 14 | 1834.68380 | 917.84554 | 612.23278 | N | 576.31000 | 288.65864 | 192.77485 | 5 |
| 15 | 1935.73148 | 968.36938 | 645.91534 | T | 462.26707 | 231.63717 | 154.76054 | 4 |
| 16 | 2034.79989 | 1017.90358 | 678.93815 | V | 361.21939 | 181.11334 | 121.07798 | 3 |
| 17 | 2121.83192 | 1061.41960 | 707.94882 | S | 262.15098 | 131.57913 | 88.05518 | 2 |
| 18 |  |  |  | R | 175.11895 | 88.06311 | 59.04450 | 1 |

**b**

| #1 | b^+^ | b^2+^ | b^3+^ | Seq | y^+^ | y^2+^ | y^3+^ | #2 |
| --- | --- | --- | --- | --- | --- | --- | --- | --- |
| 1 | 161.03793 | 81.02260 | 54.35083 | C-Carbamidomethyl |  |  |  | 18 |
| 2 | 274.12199 | 137.56463 | 92.04551 | I | 2215.87928 | 1108.44328 | 739.29794 | 17 |
| 3 | 345.15910 | 173.08319 | 115.72455 | A | 2102.79521 | 1051.90125 | 701.60326 | 16 |
| 4 | 492.19450 | 246.60089 | 164.73635 | M-Oxidation | 2031.75810 | 1016.38269 | 677.92422 | 15 |
| 5 | 652.22515 | 326.61621 | 218.07990 | C-Carbamidomethyl | 1884.72270 | 942.86499 | 628.91242 | 14 |
| 6 | 783.26564 | 392.13646 | 261.76006 | M | 1724.69205 | 862.84966 | 575.56887 | 13 |
| 7 | 898.29258 | 449.64993 | 300.10238 | D | 1593.65157 | 797.32942 | 531.88871 | 12 |
| 8 | 1054.39369 | 527.70048 | 352.13608 | R | 1478.62462 | 739.81595 | 493.54639 | 11 |
| 9 | 1297.42335 | 649.21531 | 433.14597 | Y-Phospho | 1322.52351 | 661.76540 | 441.51269 | 10 |
| 10 | 1428.46383 | 714.73555 | 476.82613 | M | 1079.49385 | 540.25057 | 360.50280 | 9 |
| 11 | 1543.49078 | 772.24903 | 515.16844 | D | 948.45337 | 474.73032 | 316.82264 | 8 |
| 12 | 1614.52789 | 807.76758 | 538.84748 | A | 833.42643 | 417.21685 | 278.48033 | 7 |
| 13 | 1800.60720 | 900.80724 | 600.87392 | W | 762.38931 | 381.69829 | 254.80129 | 6 |
| 14 | 1914.65013 | 957.82870 | 638.88823 | N | 576.31000 | 288.65864 | 192.77485 | 5 |
| 15 | 2015.69781 | 1008.35254 | 672.57079 | T | 462.26707 | 231.63717 | 154.76054 | 4 |
| 16 | 2114.76622 | 1057.88675 | 705.59359 | V | 361.21939 | 181.11334 | 121.07798 | 3 |
| 17 | 2201.79825 | 1101.40276 | 734.60427 | S | 262.15098 | 131.57913 | 88.05518 | 2 |
| 18 |  |  |  | R | 175.11895 | 88.06311 | 59.04450 | 1 |
