# Supplementary material for: Galactokinase 1 Positively Regulates Mitochondrial Respiration by Phosphorylating TIMM13 as a Protein Kinase
Source: Adv Sci (Weinh). 2026 Sep 30:e77996. Online ahead of print. doi: 10.1002/advs.77996 (PMC13625921; doi:10.1002/advs.77996)
Supplement: Supplementary file 3 [file ADVS-9999-e77996-s007.docx]

**Table S4. Information on matched fragment ions corresponding to the LC-MS/MS spectra of Figure S3. a** Fragment ions detected in LC-MS/MS spectra of CIAMCMDRYMDAWNTVSR in un-phosphorylated. **b** Fragment ions detected in MS/MS spectra of CIAMCMDRYMDAWNTVSR in phosphorylated. Matched b ions colored in red and y ions in blue. `

**a**

| #1 | b^+^ | b^2+^ | b^3+^ | Seq | y^+^ | y^2+^ | y^3+^ | #2 |
| --- | --- | --- | --- | --- | --- | --- | --- | --- |
| 1 | 161.03793 | 81.02260 | 54.35083 | C-Carbamidomethyl |  |  |  |  |
| 2 | 274.12199 | 137.56463 | 92.04551 | I | 2151.90786 | 1076.45757 | 717.97414 | 17 |
| 3 | 345.15910 | 173.08319 | 115.72455 | A | 2038.82380 | 1019.91554 | 680.27945 | 16 |
| 4 | 476.19959 | 238.60343 | 159.40471 | M | 1967.78668 | 984.39698 | 656.60041 | 15 |
| 5 | 636.23024 | 318.61876 | 212.74826 | C-Carbamidomethyl | 1836.74620 | 918.87674 | 612.92025 | 14 |
| 6 | 783.26564 | 392.13646 | 261.76006 | M-Oxidation | 1676.71555 | 838.86141 | 559.57670 | 13 |
| 7 | 898.29258 | 449.64993 | 300.10238 | D | 1529.68015 | 765.34371 | 510.56490 | 12 |
| 8 | 1054.39369 | 527.70048 | 352.13608 | R | 1414.65321 | 707.83024 | 472.22259 | 11 |
| 9 | 1217.45702 | 609.23215 | 406.49052 | Y | 1258.55210 | 629.77969 | 420.18888 | 10 |
| 10 | 1364.49242 | 682.74985 | 455.50232 | M-Oxidation | 1095.48877 | 548.24802 | 365.83444 | 9 |
| 11 | 1479.51936 | 740.26332 | 493.84464 | D | 948.45337 | 474.73032 | 316.82264 | 8 |
| 12 | 1550.55647 | 775.78188 | 517.52368 | A | 833.42643 | 417.21685 | 278.48033 | 7 |
| 13 | 1736.63579 | 868.82153 | 579.55011 | W | 762.38931 | 381.69829 | 254.80129 | 6 |
| 14 | 1850.67871 | 925.84300 | 617.56442 | N | 576.31000 | 288.65864 | 192.77485 | 5 |
| 15 | 1951.72639 | 976.36683 | 651.24698 | T | 462.26707 | 231.63717 | 154.76054 | 4 |
| 6 | 2050.79481 | 1025.90104 | 684.26979 | V | 361.21939 | 181.11334 | 121.07798 | 3 |
| 17 | 2137.82683 | 1069.41706 | 713.28046 | S | 262.15098 | 131.57913 | 88.05518 | 2 |
| 18 |  |  |  | R | 175.11895 | 88.06311 | 59.04450 | 1 |

**b**

| #1 | b^+^ | b^2+^ | b^3+^ | Seq | y^+^ | y^2+^ | y^3+^ | #2 |
| --- | --- | --- | --- | --- | --- | --- | --- | --- |
| 1 | 161.03793 | 81.02260 | 54.35083 | C-Carbamidomethyl |  |  |  | 18 |
| 2 | 274.12199 | 137.56463 | 92.04551 | I | 2199.88436 | 1100.44582 | 733.96631 | 17 |
| 3 | 345.15910 | 173.08319 | 115.72455 | A | 2086.80030 | 1043.90379 | 696.27162 | 16 |
| 4 | 476.19959 | 238.60343 | 159.40471 | M | 2015.76319 | 1008.38523 | 672.59258 | 15 |
| 5 | 636.23024 | 318.61876 | 212.74826 | C-Carbamidomethyl | 1884.72270 | 942.86499 | 628.91242 | 14 |
| 6 | 767.27072 | 384.13900 | 256.42842 | M | 1724.69205 | 862.84966 | 575.56887 | 13 |
| 7 | 882.29766 | 441.65247 | 294.77074 | D | 1593.65157 | 797.32942 | 531.88871 | 12 |
| 8 | 1038.39877 | 519.70303 | 346.80444 | R | 1478.62462 | 739.81595 | 493.54639 | 11 |
| 9 | 1281.42843 | 641.21786 | 427.81433 | Y-Phospho | 1322.52351 | 661.76540 | 441.51269 | 10 |
| 10 | 1412.46892 | 706.73810 | 471.49449 | M | 1079.49385 | 540.25057 | 360.50280 | 9 |
| 11 | 1527.49586 | 764.25157 | 509.83680 | D | 948.45337 | 474.73032 | 316.82264 | 8 |
| 12 | 1598.53298 | 799.77013 | 533.51584 | A | 833.42643 | 417.21685 | 278.48033 | 7 |
| 13 | 1784.61229 | 892.80978 | 595.54228 | W | 762.38931 | 381.69829 | 254.80129 | 6 |
| 14 | 1898.65522 | 949.83125 | 633.55659 | N | 576.31000 | 288.65864 | 192.77485 | 5 |
| 15 | 1999.70289 | 1000.35509 | 667.23915 | T | 462.26707 | 231.63717 | 154.76054 | 4 |
| 16 | 2098.77131 | 1049.88929 | 700.26195 | V | 361.21939 | 181.11334 | 121.07798 | 3 |
| 17 | 2185.80334 | 1093.40531 | 729.27263 | S | 262.15098 | 131.57913 | 88.05518 | 2 |
| 18 |  |  |  | R | 175.11895 | 88.06311 | 59.04450 | 1 |
